# Validation of the Chinese version of the Contrast Avoidance Questionnaires

**DOI:** 10.3389/fpsyg.2026.1643139

**Published:** 2026-03-13

**Authors:** Jieting Zhang, Sandra J. Llera, Mingcong Tang, Haiyu Zhong, Michelle G. Newman

**Affiliations:** 1 College of Psychology, Shenzhen University, Shenzhen, China; 2 Department of Psychology, Towson University, Towson, MD, United States; 3 Department of Psychological and Brain Sciences, Boston University, Boston, MA, United States; 4 Department of Psychology, The Pennsylvania State University, University Park, PA, United States

**Keywords:** assessment, Chinese sample, contrast avoidance questionnaires, generalized anxiety disorder, validity

## Abstract

**Objective:**

The current study aimed to validate the Chinese version of Contrast Avoidance Questionnaires (CAQs), developed based on the Contrast Avoidance Model.

**Method:**

In Study 1, we translated the CAQs into Chinese and modified them after back-translation. We then used two separate Chinese samples (*N* = 350 and 309, respectively) to explore and validate the factor structure of both CAQ measures. In Study 2, we used a third sample of Chinese college students (*N* = 388) to examine reliability and construct validity.

**Results:**

The original 3 and 2-factor structures for CAQ-Worry and CAQ-General Emotion, respectively, were replicated with acceptable reliability. Compared to the original version, both subscales had acceptable convergent, discriminant, and predictive validity for GAD symptomatology.

**Conclusion:**

Validation of the CAQs in Chinese allows for future studies of cultural differences in avoiding emotional contrasts and their relationship with mental health problems.

## 1 Introduction

Accumulating literature (e.g., Baik and Newman, 2023; Crouch et al., 2017; Llera and Newman, 2010, 2014, 2017, 2023; Newman et al., 2013, 2019, 2022, 2023) supports the Contrast Avoidance Model (CAM) of generalized anxiety disorder (GAD) proposed by Newman and Llera (2011), suggesting a central role of avoiding negative emotional contrasts in predicting GAD and its related symptoms (e.g., worry). From a functional perspective, the Contrast Avoidance (CA) model suggests that the persistence of dysfunctional and repetitive negative thinking (e.g., worry, rumination) increases and sustains negative emotion to avoid negative emotional contrasts, which has been supported by experimental (Jamil and Llera, 2021; Kim and Newman, 2022; Llera and Newman, 2010, 2014) and naturalistic (Baik and Newman, 2023; Baik and Newman, 2025; Newman et al., 2019; Newman et al., 2022; Vîslă et al., 2021) research studies.

Based on the theoretical and empirical literature on the CAM, Llera and Newman (2017) developed the Contrast Avoidance Questionnaires (CAQs). They were designed to assess the main tenets of the CAM experienced by individuals with GAD: (1) being threatened by sharp increases in negative emotion, (2) dampening mood via worry or other means to create and sustain negative emotion to avoid negative emotional contrasts, and (3) discomfort with sustained resting positive states, but not transient positive states (Llera and Newman, 2017). The CAQs contain two separate questionnaires. The CAQ-Worry (CAQ-W) emphasizes the role of worry to dampen mood as a means to avoid a negative emotional contrast and consists of three factors: Worry to Avoid Negative Emotional Shifts (F1), Worry Creates and Sustains Negative Emotion (F2), and Worry to Create a Positive Emotional Contrast (F3). The CAQ-General Emotion (CAQ-GE) reflects general mood dampening not tied to a specific method (i.e., without mentioning worry) to increase and sustain negative emotion as well as beliefs and behaviors to avoid a negative emotional contrast. It contains two factors: Creating and Sustaining Negative Emotion to Avoid Negative Contrasts (F1), and Discomfort with Emotional Shifts (F2). Generally, in the questionnaires, CA encompasses multiple facets: it refers to behaviors aimed at engaging in mood-dampening strategies to reduce or avoid negative contrast experiences and increase the probability of positive contrast experiences (i.e., emotional processes), as well as the belief that such strategies are helpful for coping with negative events.

In the initial development study among US undergraduate samples (Llera and Newman, 2017), exploratory factor analyses (EFA) and confirmatory factor analyses (CFA) supported a 3-factor structure of 30 items for the CAQ-W, and a 2-factor structure of 25 items for the CAQ-GE. The final measures were shown to have sound psychometric properties in terms of internal and retest reliability, convergent and divergent validity, as well as potential utility in predicting symptoms of GAD (Llera and Newman, 2017). More recent studies have expanded support for the predictive validity of the CAQs. For instance, both the CAQ-W and CAQ-GE and their combined score were significant predictors of GAD, even controlling for other well-established constructs in the GAD literature (i.e., intolerance of uncertainty and negative problem orientation; Llera and Newman, 2023). The CAQ-GE was also found to be a transdiagnostic predictor of probable social anxiety disorder and major depressive disorder (Newman et al., 2023), panic disorder symptoms in a diagnosed clinical sample (Gerdan, 2025), and was associated with bipolar spectrum disorders (Kim et al., 2024) and posttraumatic stress symptoms (Fite et al., 2025). Further both the CAQ-GE and CAQ-W were able to accurately detect probable OCD (Swisher and Newman, 2025).

Psychometric studies of the CAQs are also accumulating among US community adults (Rogers et al., 2023; White et al., 2021). Furthermore, the CAQs have been translated into Persian (Javaherirenani et al., 2021; Javaherirenani et al., 2025; Rashtbari et al., 2023), Turkish (Cömertoğlu Yalçın et al., 2025), and Japanese versions (Matsumoto et al., 2026). These studies replicated the 2-factor structure for the CAQ-GE, but some of them found inconsistent factor structures for the CAQ-W. For instance, White et al. (2021) found a similar model fit in the CFA for both the 2 and 3-factor models of the CAQ-W, but suggested the 2-factor model to be more parsimonious. Rashtbari et al. (2023) found that a 3-factor solution for the Iranian version of CAQ-W yielded several items with non-significant loadings or cross-loadings. In these studies, worry to avoid negative shifts and create positive contrast (i.e., F1 and F3 per the original version by Llera and Newman, 2017) were merged into a new single factor of worry to manage contrast. However, outcomes were not consistent across all studies, as Rogers et al. (2023) noted that the correlated 3-factor model fit better than the correlated 2-factor model in CFA.

Thus far, the CAQs have not been adapted to the Chinese culture, which emphasizes maintaining calm via contra-hedonic emotion regulation (decreasing positive emotions and increasing negative emotions) and relies more on avoidance and suppression (Song et al., 2024), as compared to Western cultures (Miyamoto et al., 2014). Therefore, it may be particularly important to investigate whether the CAQs could generalize to the Chinese population, especially as a means to indicate symptoms related to anxiety and emotional dysregulation. This would enhance understanding of the functional role of CA in anxiety and emotional regulation within the Chinese cultural context, where contra-hedonic emotion regulation is emphasized. It may further help identify individuals who engage in maladaptive worry or emotion regulation strategies and inform intervention targets adapted for this population. Given that little is known about the factor structure, reliability, and validity of CAQs for the Chinese population, the current study aimed to examine the psychometric properties of a newly developed Chinese version of the CAQs. Study 1 was conducted to explore and verify the underlying factor structure of the CAQ-W and CAQ-GE among the Chinese population. Study 2 aimed to examine the internal consistency, retest reliability, and construct validity (i.e., convergent and divergent validity) for the two measures.

## 2 Study 1

### 2.1 Methods

#### 2.1.1 Participants

A sample size of 300 was targeted for both EFA and CFA, which prior simulations with more complex five-factor structures have shown to be sufficient, and was therefore deemed more than adequate for the present three-factor (or simpler) model (Mundfrom et al., 2005; Myers et al., 2011; Reio and Shuck, 2014). Participants were recruited by a group of trained postgraduate students and were instructed to fill out online questionnaires by computer or mobile phone. No additional inclusion/exclusion criteria were applied beyond being a Chinese college student and providing informed consent. Participants received 10 Chinese Yuan (CNY) as compensation for their time. Participation was voluntary and they could withdraw at any time without negative consequences. Group 1 collected in Wave 1 comprised 350 Chinese college students (35.71% males, *M*_age_ = 20.13, *SD* = 1.34) and was used for the EFA. For the subsequent CFA, 309 Chinese college students (Group 2, 44% males, *M*_age_ = 20.87, *SD* = 1.78) were recruited. Study 1 and 2 were approved by the Ethics Committee of the College of Psychology, University (approval number: PN-202200049).

#### 2.1.2 Measures

*Contrast Avoidance Questionnaires (CAQs)*. The original 25 item CAQ-GE, and 30 item CAQ-W developed by Llera and Newman (2017) were translated into Chinese by a researcher and masters-degree student in Psychology with fluent English skills. All items were back-translated into English by a bilingual speaker who was blinded to the original version. Inconsistencies between the back-translated items and the original ones were fully discussed by the translators and the developers of the original CAQs until a consensus was reached. Prior to the large-scale administration, a pilot test was conducted to examine participants’ comprehension of the translated items, and minor modifications were made to improve clarity. As with the original CAQs, the Chinese version entailed two subscales: CAQ-Worry (CAQ-W; 30-items), and CAQ-General Emotion (CAQ-GE; 25-items). These were rated on a 5-point Likert scale ranging from 1 (not at all true) to 5 (absolutely true). Sum scores were created for the respective subscales, with higher scores indicating higher levels of contrast avoidance.

#### 2.1.3 Analytic approach

All analyses and data processing in this study were performed using R (version 4.4.1; R Core Team, 2024), with analyses implemented through relevant R packages or other statistical software as needed. The EFA was run separately on each CAQ measure by Psych package (Revelle, 2025). We applied oblique rotation (Oblimin) based on the assumption of factor intercorrelations, used a polychoric correlation matrix to treat the 5-point Likert items as ordinal variables, and employed the minimum residual (MINRES) extraction because it does not require the assumption of multivariate normality. A parallel analysis (Hayton et al., 2004; Horn, 1965), Velicer’s MAP (Velicer, 1976), the Hull method (Lorenzo-Seva et al., 2011) scree test, and eigenvalues were used to determine the accurate number of factors to be extracted. After factors were identified, we retained items that had loadings above 0.40 onto one component while with a loading difference of at least 0.20 on all other components (Matsunaga, 2010). Finally, to verify the factor structure, we conducted CFA in Mplus 8.3 via MplusAutomation (Hallquist and Wiley, 2018), using the robust diagonally weighted least squares estimator (WLSMV), which treats items as categorical. We further ran a CFA using the maximum likelihood estimator with the robust standard errors estimator (MLR) as a sensitivity analysis, in which items were treated as continuous. *Post hoc* modification indices (MIs) were not applied to refine the CFA model.

### 2.2 Results

#### 2.2.1 Exploratory factor analysis

For both measures, most Kaiser-Meyer-Olkin (KMO) (Shrestha, 2021) statistics of sample adequacy were above 0.9, except for items 28 and 29 from the W subscale, and items 1, 14, 19 and 22 from the GE subscale, which ranged from 0.82 to 0.88; Bartlett’s test of sphericity was significant (CAQ-W: χ^2^ [435] = 8842.59, *p* < 0.001; CAQ-GE: χ^2^ [300] = 6996.89, *p* < 0.001), indicating that the sample was suitable for factor analysis.

*CAQ-W.* The initial EFA revealed three factors with eigenvalues > 1.0; results of parallel analyses, Velicer’s MAP and Hull method (based on CAF, CFI and RMSEA), and scree plot (see Supplementary Figure A1) consistently supported a 3-factor model, which was consistent with the factor structure in the original study by Llera and Newman (2017). Hence, we re-ran the EFA constrained to three factors, and dropped items that did not meet screening rules. Specifically, Item 26 (“When I worry I can hold my emotions in a steady state”) had a low factor loading (< 0.40), and Items 22 (“I would rather worry and be surprised if something good happens, than feel good and be distressed if something bad happens”) and 11 (“If I worry, I feel so much better when things turn out okay than if I had not been worried”) cross-loaded on F1 and F3. Thus, these three items were deleted. Item 25 (“I find worrying most rewarding when something good happens in the end”) was removed in the second EFA, due to cross-loadings with F1 and F3. Additionally, Item 30 (“It is better to have worried first and then get the best outcome than to expect the best all along”), which was originally developed for F3, consistently loaded more strongly on F1 than on F3 across all EFAs, including the EFA conducted after the aforementioned items were removed. Given that F1 already contained 13 items, Item 30 was removed. All remaining items met screening rules in the final EFA. The final CAQ-W retained 25 items loading onto three factors. Factor loadings and communalities of each item are presented in Table 1. F1 (Worry to Avoid Negative Emotional Shifts; 13 items) showed a moderately positive correlation with F2 (Worry Creates and Sustains Negative Emotion; 9 items; *r* = 0.27) and F3 (Worry to Create Positive Contrast; 3 items; *r* = 0.36). F2 was moderately positively correlated with F3 (*r* = 0.40). No correlations exceeded *r* = 0.70, suggesting no problems with multicollinearity (Kline, 2005).

**Table 1:** Exploratory factor analysis for the CAQ-W.

CAQ-W items		Factor			
		1	2	3	*h* ^2^
F1: worry to avoid negative emotional shifts					
17	我对事情感到担忧，因为允许自己感到快乐最终会容易让自己感觉糟糕。 [I worry about things because allowing myself to feel happy leaves me vulnerable to feeling terrible in the end.]	**0.86**	0.13	−0.2	0.71
1	因为坏事随时都可能发生，所以我觉得担忧会让我更舒服。 [Because bad things could happen at any time, I find it more comfortable to be worried.]	**0.82**	−0.01	−0.05	0.64
20	我觉得比起放松的时候，我在担忧的时候更能控制局面。 [I feel like I have more control over the situation when I’m worried than when I am relaxed.]	**0.81**	−0.1	−0.06	0.6
15	我担忧是为了防止自己的情绪突然变糟糕。 [I worry to prevent my emotions from dropping suddenly.]	**0.79**	0.02	0.07	0.67
16	当我担忧时，我感觉我的情绪就不那么脆弱了。 [I feel less emotionally vulnerable when I worry.]	**0.79**	−0.13	0.03	0.6
27	有时候我更喜欢担忧，因为当我感觉良好时，我发现自己会等待坏事发生。 [A part of me prefers to be worried, because when I feel good, I find myself waiting for the other shoe to drop.]	**0.79**	0.02	0	0.63
24	我宁愿担忧而不是感到乐观，因为我知道负面事件会随时带走我的快乐。 [I prefer to worry rather than feel optimistic, because I know that at any moment a negative event could take my happiness away.]	**0.78**	0.07	0.02	0.66
2	我觉得我在担忧时更能控制自己的情绪。 [When I’m worrying, I feel like I’m more in control of my emotions.]	**0.77**	−0.18	−0.01	0.55
19	与其让外部事件左右自己的情绪起伏，我宁愿感到担忧以控制自己的情绪。 [I worry to control my own emotions rather than have external events control my ups and downs.]	**0.77**	0.03	0.05	0.64
10	我担忧是为了防止情绪的突然变化。 [I worry to guard against sudden shifts in my mood.]	**0.76**	0.1	0.02	0.63
9	我宁愿让自己担忧以保持稳定的情绪状态，也不愿让自己高兴后又心情变差。 [I worry to maintain a steady emotional state, rather than let myself be happy and later feel bad.]	**0.67**	0.07	0.17	0.61
12	忧虑比感觉良好后又被消极的事情弄得措手不及要好。 [Worrying is better than feeling good and then being thrown off by a negative event.]	**0.67**	0.07	0.18	0.62
23	如果我感到担忧，我就能在我需要的时候随时做好有坏情绪到来的心理准备。 [If I worry, I can stay emotionally prepared for as long as I need to.]	**0.67**	0.04	0.21	0.62
F2: worry creates and sustains negative emotion					
29	担忧会增加我的负面情绪。 [Worrying increases my bad feelings.]	−0.06	**0.88**	−0.14	0.68
28	担忧对我来说是不愉快的体验。 [Worrying is an unpleasant experience for me.]	−0.07	**0.86**	−0.2	0.61
8	担忧会使我感觉很糟糕。 [Worrying makes me feel terrible.]	−0.03	**0.82**	0.07	0.71
13	在我忧虑时，我会一直感到紧张与不安。 [When I worry, I constantly feel on edge.]	0.16	**0.76**	0.07	0.73
21	当我担忧时，我最终会变得精神紧张。 [When I worry, I end up feeling worked up.]	0.13	**0.75**	−0.04	0.61
18	在我开始担忧时，我会感到更加焦虑不安。 [I feel more agitated when I start worrying.]	0.22	**0.74**	0.03	0.7
7	当我担忧时，我感到有压力。 [When I am worrying, I feel stressed.]	0.04	**0.73**	0.23	0.74
5	忧虑使我心情不好。 [Worry keeps me in a bad mood.]	−0.11	**0.72**	0.28	0.7
4	担忧会增加我的焦虑。 [Worrying increases my anxiety.]	−0.05	**0.67**	0.26	0.64
F3: worry to create positive contrast					
6	如果我担心最坏的结果，而当结果是好的话，我会更加感激。 [If I worry about the worst outcome, I will appreciate it more when it turns out okay.]	0	0.15	**0.78**	0.73
3	当我担忧一件事会失败但最终却成功时，我更能享受它的成功。 [I enjoy success the most when I worried about failure.]	0.22	−0.04	**0.61**	0.49
14	如果我事先为结果担忧，我会更感激好事发生。 [I am more appreciative of the good things that come if I worried about the outcome beforehand.]	0.3	0.16	**0.54**	0.62

Numbers in bold represent factor loading above 0.4. h^2^ represents communalities.

*CAQ-GE.* The initial EFA demonstrated three factors with eigenvalues > 1.0, Velicer’s MAP also suggested three factors, whereas parallel analysis suggested four factors, and results from both the Hull method (based on CAF, CFI and RMSEA) and scree plot (see Supplementary Figure A2) indicated two factors. We therefore re-ran the EFA constrained to two, three, and four factors. The additional factors beyond the two-factor solution were not supported, as they did not yield sufficient numbers of items meeting the loading criteria. Specifically, in the three-factor solution, Items 2, 4, and 13 exhibited cross-loadings on Factors 1 and 3, leaving only one item (Item 1) with a salient loading on the third factor. In the four-factor solution, Items 4, 8, and 13 showed cross-loadings on Factors 1 and 3, leaving only two items (Items 1 and 2) on the third factor, and no items demonstrated adequate loadings on the fourth factor. Accordingly, the two-factor solution was retained, as all items had distinctively high loadings on their respective factors, with one exception. Item 13 (“When I have already been in a bad mood, it has been easier to endure bad news”) was deleted for low factor loading (< 0.40). The second EFA demonstrated that all remaining items met screening rules, which indicated a stable factor structure. The final CAQ-GE retained 24 items loading onto two factors. Factor loadings and the communalities of each item are presented in Table 2. F1 (Creating and Sustaining Negative Emotion to Avoid Negative Contrasts; 17 items) was moderately positively correlated with F2 (Discomfort with Emotional Shifts; 7 items; *r* = 0.543).

**Table 2:** Exploratory factor analysis for the CAQ-GE.

CAQ-GE items		Factor		
		1	2	*h* ^2^
F1: creating and sustaining negative emotion to avoid negative contrasts				
18	我保持着消极的心境，因为这让我在坏事发生时更容易应对。 [I maintain a negative mood because it makes it easier to cope when bad things happen.]	**0.84**	−0.04	0.68
5	因为坏事随时可能发生，所以提前处于一个沮丧的心情会令我感到更舒服。 [Because bad things could happen at any time, it’s more comfortable to already be in a gloomy mood.]	**0.82**	0.04	0.71
7	我更倾向于保持悲观，这样当好事发生时我就会感到惊喜。 [I prefer to have a pessimistic outlook, so that I can be pleasantly surprised if something good happens.]	**0.82**	0.02	0.69
8	我倾向于预测事情会失败，因为我不喜欢对那些万一会落空的事情怀有期待。 [I tend to predict failure because I do not like to look forward to something in case it does not happen.]	**0.81**	0.04	0.69
17	我不期待好事的发生，这样一切都会让人感到惊喜。 [I do not anticipate that anything good will happen so that everything will feel like a pleasant surprise.]	**0.81**	−0.11	0.58
20	我会关注消极的方面，因为这样至少我知道不会有太多让我感觉更糟的事情发生。 [I focus on the negative because at least I know not much can happen that could make me feel worse.]	**0.79**	0.03	0.65
9	若留意到自己高兴，我常常会立马提醒自己所有可能发生的坏事。 [If I notice I’m feeling happy, I tend to immediately remind myself of all the bad things that could happen.]	**0.78**	−0.08	0.55
2	我倾向于期待最坏的结果，这样我就不会在情绪上感到措手不及。 [I tend to expect the worst outcome so that I am not emotionally caught off guard.]	**0.77**	−0.09	0.52
12	我宁愿现在感觉不好，以免之后需要忍受失去快乐的感觉。 [I prefer to feel bad now so I do not have to endure losing my happiness later.]	**0.77**	0.06	0.65
24	我试图把注意力放在可能发生的坏事上，因为这能防止我感到情绪上的脆弱。 [I try to stay focused on the bad things that could happen, because it prevents me from feeling emotionally vulnerable.]	**0.77**	0.05	0.65
10	我从不抱太大希望，这样我就不会失望。 [I never get my hopes up so that I am not disappointed.]	**0.76**	−0.08	0.52
25	我有时宁愿现在就感觉糟糕，而不是等着看事情发展至最终的结果。 [Sometimes I would rather just feel bad now, instead of having to wait and see how things are going to turn out.]	**0.76**	0.04	0.62
16	在放松或平静的时候，我会关注负面的事情，以免让我的心情因坏事的发生而突然变化。 [When I am relaxed or calm, I focus on the negative as a way to avoid a sudden shift in my mood if something bad happens.]	**0.72**	0	0.52
4	我宁愿现在感觉糟糕，因为这样我起码不会在坏事发生时体验像过山车式起伏波动的情绪。 [I would rather feel bad now, because at least I will not experience an emotional rollercoaster if terrible things happen.]	**0.71**	0.07	0.57
22	允许自己感到快乐最终会容易让自己感觉糟糕。 [Allowing myself to feel happy leaves me vulnerable to feeling terrible in the end.]	**0.62**	0.14	0.49
21	我宁愿感到情绪低落，也不愿经历起起落落的生活。 [I would rather feel down than have to go through life experiencing ups and downs.]	**0.6**	0.15	0.49
1	我关注事情消极的方面，因为万一糟糕的事情发生，我想在心理上有所准备。 [I focus on the negative because I want to be emotionally prepared in case something terrible happens.]	**0.53**	0.08	0.34
F2: discomfort with emotional shifts				
19	情绪的起伏会让我感到不适。 [When my emotions go up and down, it makes me uncomfortable.]	−0.11	**0.95**	0.8
11	突如其来的坏情绪让我感到十分措手不及。 [It really throws me off when I suddenly feel very bad.]	−0.01	**0.8**	0.63
23	强烈的情绪波动对我来说特别不舒服。 [Strongly fluctuating emotions are particularly unpleasant for me.]	0.03	**0.77**	0.61
15	情绪的波动会让我感到失控。 [When my emotions fluctuate it makes me feel out of control.]	0.09	**0.76**	0.67
6	情绪急剧向负面转变会令我感到特别不安。 [I am particularly uneasy with sharp shifts in my negative emotion.]	0.11	**0.7**	0.58
3	我对自己的情绪变化感到不安。 [I feel uneasy with emotional changes.]	0.24	**0.56**	0.52
14	我不喜欢自己的情绪受到外部事件的控制而大起大落。 [I do not like it when external events control my ups and downs.]	0.09	**0.51**	0.32

Numbers in bald represent factor loading above 0.4. h^2^ represents communalities.

Details including the KMOs and 95% bootstrap confidence intervals of the factor loadings for each item under the aforementioned factor solutions are presented in Supplementary Tables A1–A8.

#### 2.2.2 Confirmatory factor analysis

*CAQ-W.* To verify the factor structure for the CAQ-W, we compared the 3-factor 25 item model, as determined by EFA, with rival 1- and 2-factor models (i.e., combining all factors for the 1-factor model, and combining F1 and F3 to create a single factor reflecting worry to manage contrast for the 2-factor model). In addition, the original 3-factor model per Llera and Newman (2017), in which none of the original items were deleted, was also included for comparison. The CFA generally supported a 3-factor model with 25 items that fit better than a 1-factor model and slightly better than 2-factor model, and was comparable with the original 3-factor model (see Table 3). Overall, most indices for this model indicated an acceptable model fit (χ^2^ = 863.32, *df* = 272, χ^2^/*df* = 3.17, CFI = 0.95, TLI = 0.95, SRMR = 0.07, RMSEA = 0.08 [90% CI = 0.08–0.09]). For this model, standardized factor loadings ranged from 0.69 to 0.86 (all *p*s < 0.001). Thus, the 3-factor 25 item model was retained for subsequent analyses in Study 2.

**Table 3:** Comparison of measure model.

Measure	Model	S-Bχ^2^	*df*	SRMR	RMSEA	CFI	TLI
CAQ-W	1-factor	3177.08	275	0.19	0.18 (0.18, 0.19)	0.78	0.76
	2-factor	1047.63	274	0.08	0.1 (0.09, 0.1)	0.94	0.94
	3-factor	863.32	272	0.07	0.08 (0.08, 0.09)	0.95	0.95
	3-factor (without deleting items)	1304.45	402	0.07	0.09 (0.08, 0.09)	0.94	0.94
CAQ-GE	1-factor	1470.64	252	0.08	0.12 (0.12, 0.13)	0.92	0.91
	2-factor	729.23	251	0.05	0.08 (0.07, 0.09)	0.97	0.97
	2-factor (without deleting items)	755.42	274	0.05	0.07 (0.07, 0.08)	0.97	0.97

S-Bχ^2^, Satorra-Bentler χ^2^; df, degrees of freedom; SRMR, standardized root mean square residual; RMSEA, root mean square error of approximation; CFI, comparative fit index; TLI, Tucker-Lewis index. Boldface represents the model with the best overall fit.

*CAQ-GE.* The CFA indicated the 2-factor model with 24 items fit better than a 1-factor model, and comparable with the 2-factor without deleting any items (Table 3). Key fit indices for this model were acceptable (χ^2^ = 729.23, *df* = 251, χ^2^/*df* = 2.91, CFI = 0.97, TLI = 0.97, SRMR = 0.05, RMSEA = 0.08 [90% CI = 0.07–0.09]). Standardized factor loadings ranged from 0.54 to 0.87 (all *p*s < 0.001) for the 2-factor model with 24 items. Thus, this model was retained for subsequent analyses in Study 2.

Sensitivity analysis using an MLR estimator consistently suggested a 3-factor model with 25 items for the W scale, and 2-factor model with 24 items for the GE scale (see Supplementary Table C1), which demonstrated acceptable model fits and sufficient factor loadings. The parameters for CFA are presented in the Supplementary Material B (for WLSMV) and Supplementary Material C (for MLR). Details about the final version of the two scales (instructions, response format, and scoring) are presented in Supplementary Material D.

### 2.3 Brief discussion

The current findings from an EFA and CFA based on separate samples supported the original factor structures proposed by Llera and Newman (2017), as opposed to the revised factor structures proposed by other authors (e.g., White et al., 2021). Specifically, the CAQ-W retained 25 items constituting three latent factors: Worry to Avoid Negative Emotional Shifts (F1), Worry to Create and Sustain Negative Emotion (F2), and Worry to Create a Positive Emotional Contrast (F3), after excluding items 11, 22, 25, 26, and 30. Whereas the original items in F1 and F3, respectively, emphasize worry’s role in avoiding negative emotional changes and generating positive contrasts, these items do not clearly articulate such functions. Instead, item 26 seems to reflect worry’s function of maintaining emotional stability, and the other four items originally from F3 aligned more with a preference or belief in the value of worrying ahead of a positive outcome. Moreover, the limited adaptability of these items may also relate to common cultural beliefs in China, where worry is often regarded as an effective coping strategy in life. For instance, traditional Chinese sayings encourage people to “worry deeply and think further” (忧深思远), to “be cautious of potential dangers even in safe situations” (居安思危), and to “worry before others and enjoy after others” (先天下之忧而忧，后天下之乐而乐) (Xie, 2020). The tendency of Chinese respondents to interpret worry as a preventive and responsible coping strategy for crises or negative events may have blurred the distinction between F3 and F1, particularly weakening the discriminability of F3 items.

The CAQ-GE retained 24 items representing two latent factors: Generating Negative Emotion to Avoid Negative Contrasts (F1) and Discomfort with Emotional Shifts (F2), after excluding item 13. Other items in this dimension clearly reflected the motivation to maintain negative emotions, whereas item 13 mainly described the outcome of already being in a negative mood (i.e., finding it easier to endure bad news), making the motivational aspect less explicit and rendering this item less consistent with its intended dimension.

## 3 Study 2

### 3.1 Methods

#### 3.1.1 Participants

For Study 2, 388 Chinese college students (Group 3, 40.21% males, *M*_age_ = 20.11, *SD* = 1.11) were recruited in Wave 3, which was used to examine reliability and construct validity. A subset of participants (*N* = 134, 38.1% male, *M*_age_ = 20.104, *SD* = 1.020) in Group 3 completed the CAQ questionnaires a week later, to estimate retest reliability. The recruitment procedures and ethical safeguards were identical to those used in Study 1.

#### 3.1.2 Measures

The modified Chinese-language CAQ measures (i.e., 25-item CAQ-W; 24-item CAQ-GE), as developed in Study 1, were used in this study.

*Generalized Anxiety Disorder (GAD-7).* The GAD-7 (Spitzer et al., 2006) with 7 items assessed participants’ anxiety symptoms over the past 2 weeks. He et al. (2010) translated the GAD-7 into Chinese, and found that a cut-off score of 10 could identify Chinese adults with GAD with good sensitivity (86.2%) and specificity (95.5%). Prior literature has indicated the Chinese version had fair to good reliability and validity (e.g., Zhang et al., 2021). This scale is scored on a 4-point Likert scale ranging from 0 (never) to 3 (almost every day). A total score was created by summing all items. Internal consistency for the total score in the current sample was excellent (Cronbach’s *α* = 0.911).

*Penn State Worry Questionnaire (PSWQ).* Trait worry was assessed with the Chinese version (Sha et al., 2006) of the PSWQ (Meyer et al., 1990), which had fair to good reliability and validity in the Chinese samples (Zhong et al., 2009). This scale consists of 16 items classified by two subscales: Engagement of worry and absence of worry, scored on a 5-point Likert scale ranges from 1 (not at all typical of me) to 5 (very typical of me). Items were averaged to create a total score, with higher scores indicating higher levels of trait worry. In the current sample, Cronbach’s *α* was 0.885 for the total scale (0.64 for worry absence, 0.93 for worry engagement, respectively).

*Perceptions of Threats Questionnaire (PTEQ).* The PTEQ is a 72 items scale developed by McCubbin and Sampson (2006) and was included to assess participants’ beliefs about the threat posed by each of seven basic emotions (i.e., sadness, happiness, anger, fear, disgust, guilt, and lust) and strong emotions in general. The original scale was translated into Chinese by the authors. The current study focused on six of the basic emotions (excluding ‘lust’) and strong emotion, which were rated on a 5-point scale ranging from 1 (not at all) to 5 (definitely). Cronbach’s α was 0.816 for sadness, 0.68 for happiness, 0.82 for anger, fear, and disgust, 0.81 for guilt, and 0.84 for strong emotion. A CFA also supported that each subscale fit well in terms of CFI (0.89–0.91) and TLI (0.82 ~ 0.88), except for the Happiness subscale (CFI = 0.80 and TLI = 0.73).

*Sensation Seeking Scale (SSS)*. The trait of seeking intensity and novelty in sensory experiences was assessed with the SSS for Chinese undergraduates (Zhao, 2004). The SSS includes 36 items with 18 items for each subscale (i.e., Thrill and Adventure Seeking, and Disinhibition). It is scored on a Likert scale ranging from 1 (Do not want to do) to 3 (Want to do, do it if you have the chance). The Chinese version of the SSS had adequate reliability and validity (Zhao, 2004). An average score was derived for sensation seeking, with higher values indicating higher levels of the trait of seeking intensity and novelty. For the current study, the measure demonstrated excellent internal consistency reliability (Cronbach’s α = 0.93, 0.93, and 0.88 for the total scale, Thrill and Adventure Seeking, and Disinhibition subscales, respectively).

#### 3.1.3 Analytic approach

Firstly, Cronbach α was estimated to evaluate the internal consistency reliability for total scales and each subscale of the two CAQ measures with a threshold of 0.70, 0.80, and 0.90 indicating adequate, good, and excellent relatability, respectively (Youngstrom et al., 2017). Using the semTools package (Jorgensen et al., 2025), Omega total (*ω*) was estimated for total and subscale scores, and hierarchical omega (ω_h-cat_) was estimated for the total scales based on a bifactor model (Flora, 2020) if the model resulted in adequate fit (e.g., CFI > 0.95, RMSEA < 0.08). The average inter-item correlation (AIIC) was evaluated, and a range of 0.15–0.50 was recommended as it indicates a balance between desirable commonality and the avoidance of redundancies between items; item-total correlations were also examined for each item, with values below 0.30 considered unacceptable (Stefana et al., 2024). Then, bivariable correlations and intraclass correlation coefficients (ICC [2,1]) were used to evaluate retest reliability, with values 0.40–0.59, 0.60–0.74, and ≥0.75, respectively, considered fair, good and excellent for correlation (Cicchetti, 1994), and values < 0.50, 0.50–0.75, 0.75–0.90, and > 0.90, respectively, indicated poor, moderate, good, and excellent for ICC (Koo and Li, 2016). Secondly, to estimate construct validity we ran correlation analyses between CAQ scales and all convergent and discriminant measures, and used Steiger’s *Z* tests (Hoerger, 2013; Steiger, 1980) to determine distinctions between convergent and discriminant correlations. Thirdly, we created High and Low-symptom subgroups from within our sample (see below) and used independent-sample *t*-tests to estimate whether the CAQ measure total score and individual subscale scores in the High-symptom group were significantly higher than the Low-symptom group. Receiver operating characteristic (ROC) analyses were then conducted with the pROC package (Robin et al., 2011) to evaluate the ability of each CAQ measure to predict GAD symptomatology accurately. The area under the ROC curve (AUC) was calculated as an index of overall discrimination ability, with confidence intervals obtained using DeLong et al.’s (1988) nonparametric method. To explore the optimal cut point, we used the Youden index (J), which identifies the threshold that maximizes the combined sensitivity and specificity (Youden, 1950).

We created High- and Low-symptom subgroups according to recommendations by He et al. (2010) using the GAD-7, as well as scores on the PSWQ. Individuals with GAD-7 total scores ≥ 10 and PSWQ total scores greater than one standard deviation above the overall sample mean (*M =* 50.38, *SD* = 10.90) were included in the High-symptom group (*N* = 37; GAD-7: *M* = 14.649, *SD* = 3.327; PSWQ: *M* = 69.324, *SD* = 4.773). The PSWQ total score for this group was comparable to those with clinically diagnosed GAD found in other samples (*M* = 67.16, SD = 9.16; *Z* = 2.474, *p* < 0.01; Startup and Erickson, 2006). Participants were included in the Low-symptom group (*N* = 61) if they scored below 10 on the GAD-7 (*M* = 2.328, *SD* = 2.343), and one standard deviation below the overall sample mean on the PSWQ (Low-symptom group: *M* = 34.639, *SD* = 3.578). The PSWQ total score in the Low-symptom group was significantly lower than the score for those without clinically diagnosed GAD (*M* = 37.5, *SD* = 9.16, *Z* = −6.239, *p* < 0.001; Startup and Erickson, 2006). To balance the sample size between the two groups for *t* test, 37 Low-symptom participants were selected randomly among the Low-symptom group (*N* = 61) as a comparison sample. The demographic variables (i.e., sex, age, education, major, and ethnicity) did not differ significantly between the two groups (*t* (72) = 0.93, *p* = 0.35 for age; χ^2^(1) = 2.38, *p* = 0.123 for education; χ^2^(2) = 1.61, *p* = 0.45 for major; χ^2^(1) = 0.14, *p* = 0.71 for ethnicity and χ^2^(1) = 2.69, *p* = 0.10) for sex, which suggests lack of significant difference between the two groups.

### 3.2 Results

#### 3.2.1 Reliability

The CAQ-W demonstrated excellent internal consistency reliability for the total scale and F1 and F2 subscales (Cronbach’s *α* = 0.93, 0.93 and 0.94, Omega total *ω*s = 0.96, 0.93 and 0.94, respectively), and good for F3 (α = 0.81, ω = 0.82). As the bifactor model with three factors did not converge, ω_h-cat_ was unavailable for the W subscale. The AIICs were 0.59, 0.68 and 0.66, respectively for F1, F2, and F3. Item-total correlations ranged from 0.54 to 0.82 across the three subscales. For the CAQ-GE, the internal consistency reliability was also excellent for the total scale and F1 (αs = 0.93 for both, ωs = 0.95 and 0.94, respectively), and good for F2 (α = 0.88, ω = 0.88). ω_h-cat_ were 0.14, 0.78 and 0.87, respectively, for the general factor, F1 and F2, which indicated the variance was largely attributable to the specific factors rather than the general factor. The AIICs were 0.55 and 0.56, respectively for F1 and 2. Item-total correlations ranged from 0.44 to 0.80 across the two subscales (see Supplementary Tables E1, E2 for more details).

Correlation coefficients indicated generally good to excellent retest reliability on the CAQ-W for the total score, as well as F1 and F2 subscores (*r*s = 0.76, 0.77, 0.74, respectively, *p*s < 0.001); while ICCs indicated moderate to good retest reliability (ICCs = 0.76, 95% CI [0.68, 0.82], 0.77 [0.69, 0.83], 0.74 [0.65, 0.81], respectively). However, the CAQ-W F3 subscale showed fair (*r* = 0.54, *p* < 0.001) to moderate (ICC = 0.51 [0.35, 0.64]) retest reliability. The retest reliability of the CAQ-GE total score and its subscales was excellent or good (*r*s = 0.85, 0.86, and 0.76, *p*s < 0.001; ICCs = 0.85 [0.79, 0.89], 0.86 [0.80, 0.90] and 0.76 [0.68, 0.83]). Overall, the results suggested both CAQ measures and their subscales were repeatable within a sample over time, except for weaker stability for CAQ-W F3.

#### 3.2.2 Construct validity

Table 4 shows that, within both CAQ-W and CAQ-GE scales, all the subscales were moderately correlated with one another (range: 0.28 to 0.48, *p*s < 0.001), and strongly associated with the total scale (range: 0.70 to 0.95, *p*s < 0.001). The CAQ scales and subscales mostly showed moderate correlations with convergent construct measures (GAD-7, PSWQ, and the PTEQ negative and strong emotion subscales), with some weaker (e.g., *r* = 0.26 between W-F1 and PTEQ-fear) or stronger (e.g., *r* = 0.61 between W-F2 and PSWQ) associations observed. Additionally, these scales overall demonstrated lower correlations with divergent construct measures (SSS and PTEQ-Happy).

**Table 4:** Convergent and discriminant validity of CAQ scales and subscales.

	1	2	3	4	5	6	7	8	9	10	11	12	13	14	15	16	17
CAQ measure																	
1. CAQ_W_Total	1^***^																
2. CAQ_W_F1	0.84^***^	1^***^															
3. CAQ_W_F2	0.74^***^	0.28^***^	1^***^														
4. CAQ_W_F3	0.7^***^	0.47^***^	0.48^***^	1^***^													
5. CAQ_GE_Total	0.81^***^	0.78^***^	0.49^***^	0.47^***^	1^***^												
6. CAQ_GE_F1	0.71^***^	0.8^***^	0.3^***^	0.39^***^	0.95^***^	1^***^											
7. CAQ_GE_F2	0.69^***^	0.44^***^	0.7^***^	0.46^***^	0.73^***^	0.47^***^	1^***^										
Convergent measures																	
*GAD symptoms*																	
8. GAD-7	0.51^***^	0.38^***^	0.47^***^	0.26^***^	0.5^***^	0.43^***^	0.47^***^	1									
9. PSWQ	0.55^***^	0.31^***^	0.61^***^	0.39^***^	0.49^***^	0.37^***^	0.57^***^	0.66^***^	1								
*Perceived threat of negative emotions*																	
10. PTEQ_Sad	0.43^***^	0.29^***^	0.44^***^	0.24^***^	0.4^***^	0.33^***^	0.39^***^	0.45^***^	0.45^***^	1^***^							
11. PTEQ_Guilty	0.48^***^	0.29^***^	0.5^***^	0.29^***^	0.42^***^	0.32^***^	0.45^***^	0.45^***^	0.43^***^	0.84^***^	1^***^						
12. PTEQ_Fear	0.42^***^	**0**.26^***^	0.44^***^	0.29^***^	0.35^***^	0.27^***^	0.38^***^	0.39^***^	0.39^***^	0.76^***^	0.77^***^	1^***^					
13. PTEQ_Angry	0.44^***^	0.31^***^	0.4^***^	0.31^***^	0.38^***^	0.29^***^	0.43^***^	0.38^***^	0.36^***^	0.68^***^	0.69^***^	0.78^***^	1^***^				
14. PTEQ_Disgust	0.43^***^	0.29^***^	0.41^***^	0.29^***^	0.38^***^	0.3^***^	0.41^***^	0.41^***^	0.38^***^	0.73^***^	0.73^***^	0.79^***^	0.84^***^	1^***^			
15. PTEQ_Strong	0.47^***^	0.33^***^	0.43^***^	0.33^***^	0.41^***^	0.33^***^	0.43^***^	0.34^***^	0.36^***^	0.6^***^	0.62^***^	0.66^***^	0.78^***^	0.74^***^	1^***^		
Divergent measure																	
16. PTEQ_Happy	0.29^***^	0.37^***^	0.12^*^	0.05	0.29^***^	0.31^***^	0.15^**^	0.33^***^	0.12^*^	0.48^***^	0.46^***^	0.38^***^	0.35^***^	0.41^***^	0.32^***^	1^***^	
17. SSS	0.11^*^	0.14^**^	0.05	−0.04	0.2^***^	0.19^***^	0.15^**^	0.18^***^	0.04	0.14^**^	0.15^**^	0.14^**^	0.18^***^	0.17^***^	0.16^**^	0.17^***^	1^***^

^*^p < 0.05. ^**^p < 0.01. ^***^p < 0.001.

Table 5 presents Steiger’s *Z* comparisons between correlations with convergent and discriminant measures. In these tests, both CAQ measures and their subscales were overall significantly more positively correlated with convergent measures than with measures of sensation seeking and perceived threat of happiness (*Z* = 2.17~8.97). However, there were some exceptions. For instance, the CAQ-W F1 and CAQ-GE F1 subscales did not demonstrate significantly higher correlations with most convergent measures as compared to the PTEQ-Happy subscale (*Z* = −2.1~1.01). They also did not demonstrate higher correlations with PTEQ-Fear versus sensation seeking (*Z* = 1.74 and 1.27, respectively). Finally, the CAQ-GE Total did not correlate more highly with PTEQ-Fear versus PTEQ-Happy (*Z* = 1.08).

**Table 5:** Discriminant validity of CAQs: comparing convergent measures with divergent measures, reporting Steiger’s Z scores (ZH).

Convergent scale	Divergent scale	CAQ-W _Total	CAQ-W_F1	CAQ-W_F2	CAQ-W_F3	CAQ-GE_Total	CAQ-GE_F1	CAQ-GE_F2
GAD-7	PTEQ-Happy	4.12^***^	0.2	6.42^***^	3.74^***^	4.04^***^	2.26^*^	5.83^***^
	SSS	6.74^***^	3.8^***^	6.89^***^	4.71^***^	5.14^***^	3.97^***^	5.24^***^
PSWQ	PTEQ-Happy	4.45^***^	−0.99	8.29^***^	5.35^***^	3.45^***^	1.01	7.14^***^
	SSS	7.07^***^	2.41^*^	8.97^***^	6.37^***^	4.69^***^	2.76^**^	6.82^***^
PTEQ-Sad	PTEQ-Happy	2.95^**^	−1.55	6.45^***^	3.83^***^	2.24^*^	0.49	4.9^***^
	SSS	5.25^***^	2.32^*^	6.11^***^	4.29^***^	3.2^**^	2.26^*^	3.8^***^
PTEQ-Guilt	PTEQ-Happy	3.8^***^	−1.51	7.83^***^	4.72^***^	2.57^*^	0.34	6.16^***^
	SSS	6.04^***^	2.35^*^	7.39^***^	5.1^***^	3.53^***^	2.17^*^	4.93^***^
PTEQ-Fear	PTEQ-Happy	2.49^*^	−2.1^*^	6.11^***^	4.33^***^	1.08	−0.71	4.38^***^
	SSS	5.06^***^	1.74	6.26^***^	4.98^***^	2.36^*^	1.27	3.69^***^
PTEQ-Strong	PTEQ-Happy	3.31^***^	−0.7	5.55^***^	5^***^	2.27^*^	0.39	5.19^***^
	SSS	5.97^***^	2.96^**^	6.07^***^	5.82^***^	3.51^***^	2.26^*^	4.64^***^

^*^p < 0.05. ^**^p < 0.01. ^***^p < 0.001.

The High-symptom group scored significantly higher than the Low-symptom group on both CAQ measures and their subscales, with moderate to strong effect sizes for all comparisons (Cohen’s *d* = 1.04–2.07; see Supplementary Table E3). ROC curve analyses indicated that CAQ measures were strong predictors of GAD symptomatology (see Supplementary Figures E1, E2), with AUC of 0.91 (95% CI [0.86, 0.97]) for CAQ-W and 0.88 (95% CI [0.81, 0.95]) for CAQ-GE. This suggested the probability of a person with high GAD symptoms scoring higher than a person with low GAD symptoms on the CAQ-W and CAQ-GE was 91 and 88%, respectively. For CAQ-W, Youden index (J) indicated the cut-off point ≥2.50 gave the best combination of sensitivity (92%) and specificity (82%) for predicting GAD symptomatology, with 86% of participants correctly classified. For CAQ-GE, the cut score 2.31 provided the best combined results of sensitivity and specificity (78 and 84%) for accurately predicting GAD symptomatology, with 82% of participants correctly classified (see Supplementary Table E4 for details).

### 3.3 Brief discussion

Consistent with the original version of the CAQs, the current results generally supported excellent or good internal consistency reliability as evidenced by Cronbach’s *α* exceeding 0.80 or 0.90, and item-total correlations mostly higher than 0.50. However, the AIICs were slightly above 0.50 for all the subscales, which may have indicated redundancies between items. Total scales and subscales for both the CAQ-W and CAQ-GE generally demonstrated acceptable replicability within a week. The weaker stability for CAQ-W F3 may be due to the fact that half of the original items were removed, inducing increased random measurement error. Therefore, we do not recommend using this subscale independently at this stage, as further research is needed to improve its temporal stability.

Positive correlations of the CAQ scales with GAD symptoms, trait worry, and perceived threat of emotions supported the hypotheses that tendency to fear or avoid negative emotional contrasts was related to important characteristics of GAD, as well as higher GAD symptomatology. These findings indicated good convergent validity of the Chinese version of CAQs. In terms of discriminant validity, the CAQ-W and CAQ-GE generally showed sound divergent trends, as indicated by stronger correlations with convergent constructs than with perceived threat of happiness and thrill-seeking as divergent constructs. However, there were some exceptions, mostly for F1 in both subscales (relating to avoiding negative contrasts) which did not demonstrate significantly stronger correlations with convergent constructs as compared to divergent constructs (see General Discussion).

All CAQ scales and subscales reached substantially higher scores in High-symptom compared to Low-symptom participants, with large effect sizes. Both the CAQ-W and CAQ-GE scales significantly discriminated between individuals with high and low GAD symptoms, with a moderately large percentage of participants being correctly identified in their respective groups. Moreover, the CAQ scales demonstrated only slightly lower but still acceptable sensitivity and specificity, compared to the original version. One possible reason may have been that the current study used the GAD-7 as one means of creating GAD High and Low-symptom groups, whereas the original study used the GAD-Q-IV (Newman et al., 2002), which is more focused on the DSM criteria for GAD. These results generally replicated the original study (Llera and Newman, 2017) indicating the predictive potential of CAQ for GAD symptomatology in a Chinese sample.

## 4 General discussion

The aims of the current study were to (a) develop the Chinese version of the CAQs, by exploratory and confirmatory investigation in the factor structure, and (b) examine their reliability by internal consistency reliability and retest reliability, as well as validity by testing their convergent and discriminant validity and predictive ability for GAD symptomatology.

Consistent with the hypotheses, the current study provided initial evidence that the CAQs in the Chinese language and culture had a similar factor structure to that of the original version (i.e., three factors for CAQ-W and two factors for CAQ-GE; Llera and Newman, 2017), with generally good reliability. Notably, other psychometric tests of the CAQs (e.g., among North American community participants Rogers et al., 2023; White et al., 2021 and Iranian college students Rashtbari et al., 2023) suggested a 2-factor structure for the CAQ-W, combining F1 and F3 into one factor as a broad measure of emotional contrast. However, model comparison by CFA in the current study supported the original theoretical framework of the CAQs, in that avoiding negative emotional shifts and creating positive contrast were seen as two separate dimensions of worry in GAD. This is supported by existing research on the CAM. Experimental (Jamil and Llera, 2021; Kim and Newman, 2019, 2022, 2023; Llera and Newman, 2010, 2014) and naturalistic momentary assessment (Baik and Newman, 2023; Baik and Newman, 2025; Newman et al., 2019; Newman et al., 2022; Swisher and Newman, 2026; Vîslă et al., 2021) studies showed that individuals with GAD attempted to avoid negative emotional contrasts (i.e., sudden increases of negative emotions or decreases of positive emotions) by worrying to increase and sustain their anxiety. Research has also shown that most worry topics tend to turn out better than expected (LaFreniere and Newman, 2020). This also increases the likelihood of positive emotional contrasts (i.e., decrease of negative emotion or elevation of positive emotion; Baik and Newman, 2025; Kim and Newman, 2023; Newman et al., 2022), which further reinforces worry (Baik and Newman, 2023; Baik and Newman, 2025; Kim and Newman, 2023; Newman et al., 2022). In sum, research on the CAM suggests that negative and positive emotional contrasts may represent two different drives that motivate worry: the former related to defensive systems of avoiding danger or adversity, and the latter related to appetitive motives for the desired emotion experience.

Similar to the original version of the CAQs (Llera and Newman, 2017), the two subscales of the Chinese version had good construct validity, as well as acceptable predictivity of the CAQ-W and CAQ-GE for GAD symptoms as indicated by combined sensitivity and specificity in the best cut-off of the CAQs. However, the discriminant validity of some subscales appeared less promising. In particular, F1 scales of both CAQ-W and GE did not consistently correlate more highly with measures related to anxiety and fear of negative emotions versus discriminant measures (perceived threat of happiness and sensation-seeking). Whereas this may have indicated somewhat weakened evidence of discriminant validity for these subscales, it may also be due to the scales used for this comparison. Although we chose to use the PTEQ-Happy subscale as a divergent measure, the CAM actually suggests heterogeneity in terms of perceived threat of happiness for those with high GAD symptomatology. Transient happiness (e.g., relief when a feared outcome does not occur) may not be feared or avoided (e.g., Baik and Newman, 2025; Kim and Newman, 2023); however, sustained happiness may be experienced as more threatening (see Baik and Newman, 2023). However, the PTEQ does not indicate a duration of each target emotion. Thus, it is possible that participants may have responded to this measure in an inconsistent manner. In addition, the PTEQ was translated by the authors for this study but has not yet been validated in China, which may also have caused bias in its correlations with CAQ and the convergent measures.

Besides the potential psychometric limitation of the PTEQ scale, it is possible that the weaker index of discriminant validity may be explained by cultural differences in hedonic emotion regulation between western and eastern populations: Easterners tend to have less motivation for hedonic emotion when compared to Westerners (Deng and Ding, 2019; Hampton and Varnum, 2018; Miyamoto et al., 2014). Specifically, dialectical and “Zhongyong” thinking styles, as well as Yin Yang philosophy, are deeply cultivated and highly prevalent in China. These thinking styles state that both negativity and positivity have good and bad sides or even coexist (Deng et al., 2016), such as extreme joy begets sorrow (“乐极生悲” in Chinese). Hence, avoiding negative contrast and being wary of extreme happiness coexist among the Chinese population as a kind of coping strategy, thus potentially explaining why fear of positive emotion did not consistently function as a discriminant measure. Future studies should explore how these strategies play a role in GAD symptomatology under normal and abnormal levels of worry (Rashtbari and Saed, 2020) and their cultural differences.

The current study was limited by online convenience samples among college students, the results of which may not have been representative enough to generalize to the overall population or clinically diagnosed patients. The diagnostic groups were created solely based on self-report questionnaires, without validation through structured clinical interviews. Reliance on this less validated classification with small subsamples limits the practical significance and generalizability of the CAQs. Hence, findings warrant replication in larger samples of patients with a formal clinical diagnosis. Further, given the cultural normativity of some aspects of contrast avoidance in China, these measures should be tested within Chinese clinical samples to determine how the factors manifest among those with pathological GAD symptoms. Additionally, average inter-item correlations exceeded 0.50 for all factors, suggesting potential redundancy at the item level. Future research should consider systematic item reduction using larger and more diverse samples, based on item response theory or information-based indices, to identify items that contribute unique information while maintaining content coverage. Nevertheless, such item reduction strategies may not be suitable for CAQ-W F3, which contains only three items. Finally, future studies may focus on measurement invariance of CAQs between Western and Eastern populations and their differential associations with various kinds of anxiety (e.g., relaxation-induced anxiety).

Overall, there are many potential directions for research on the CAM in a Chinese population. Accumulating literature suggests CAQs are associated with interpersonal problems. In a sample of clients with GAD symptoms, interpersonal problems (i.e., cold-distant and socially inhibited) predicted using worry to avoid NECs and create PECs, and this in turn predicted greater GAD symptoms (Shafiei et al., 2022). Further, among individuals with GAD symptoms, interpersonal problems (e.g., affiliative, submissive, cold) predicted increased worry through engaging in interpersonal behaviors to avoid emotional contrasts (Erickson et al., 2023). These findings indicate that the CAM reflects not only anxiety symptoms but may also apply to relational difficulties. Thus, future studies should examine the associations between the CAQs and interpersonal dysfunction and provide better understanding of the clinical significance of CAQs in this domain. In addition, considering that repetitive negative thinking is a common feature across various emotional problems (e.g., Moulds and McEvoy, 2025), and given that CA may also be implicated in other disorders (e.g., posttraumatic stress; Fite et al., 2025; panic disorder; Gerdan, 2025; bipolar spectrum disorder; Kim et al., 2024; major depressive disorder and social anxiety disorder; Newman et al., 2023; obsessive-compulsive disorder; Swisher and Newman, 2025), future research could further evaluate the broader validity of the CAM by examining the associations of CAQ scores not only with GAD but also with other psychological disorders.

The benefit of the current study in developing and finding preliminarily validation of the Chinese version of CAQs is that it paves the way for future studies of the CAM in this population. The CAQs may have practical applications in clinical and research contexts, including screening for individuals with elevated risk of GAD, identifying intervention targets, and informing culturally adapted treatment strategies, particularly if future studies in clinical samples validate its diagnostic utility.

## Data Availability

The raw data supporting the conclusions of this article will be made available by the authors, without undue reservation.
