# Supplementary material for: Validation of the Chinese version of the Contrast Avoidance Questionnaires
Source: Front Psychol. 2026 Mar 13;17:1643139. doi: 10.3389/fpsyg.2026.1643139 (PMC13021620; doi:10.3389/fpsyg.2026.1643139)
Supplement: Supplementary file 1 [file Table_1.DOCX]

**Contents of Supplementary Materials**

[Supplementary Material A: Exploratory Factor Analysis 3](#_Toc221317962)

[**Table A1** *The First Exploratory Factor Analysis for the CAQ-W* 3](#_Toc221317963)

[**Table A2** *The Second Exploratory Factor Analysis for the CAQ-W* 4](#_Toc221317964)

[**Table A3** *The Third Exploratory Factor Analysis for the CAQ-W* 5](#_Toc221317965)

[**Table A4** *The Fourth Exploratory Factor Analysis for the CAQ-W* 6](#_Toc221317966)

[**Table A5** *The Two-factor Solution in the First Exploratory Factor Analysis for the CAQ-GE* 7](#_Toc221317967)

[**Table A6** *The Three-factor Solution in the First Exploratory Factor Analysis for the CAQ-GE* 8](#_Toc221317968)

[**Table A7** *The Four-factor Solution in the First Exploratory Factor Analysis for the CAQ-GE* 9](#_Toc221317969)

[**Table A8** *The Two-factor Solution in the Second Exploratory Factor Analysis for the CAQ-GE* 10](#_Toc221317970)

[**Figure A1** *The Scree Plot of the Initial Exploratory Factor Analysis for the CAQ-W* 11](#_Toc221317971)

[**Figure A2** *The Scree Plot of the Initial Exploratory Factor Analysis for the CAQ-GE* 12](#_Toc221317972)

[Supplementary Material B: Confirmatory Factor Analysis 13](#_Toc221317973)

[**Table B1** *The One-factor Model for the CAQ-W with 25 Items* 13](#_Toc221317974)

[**Table B2** *The Two-factor Model for the CAQ-W with 25 Items* 17](#_Toc221317975)

[**Table B3** *The Three-factor Model for the CAQ-W with 25 Items* 21](#_Toc221317976)

[**Table B4** *The Three-factor Model for the CAQ-W with 30 Items* 25](#_Toc221317977)

[**Table B5** *The One-factor Model for the CAQ-GE with 24 Items* 29](#_Toc221317978)

[**Table B6** *The Two-factor Model for the CAQ-GE with 24 Items* 32](#_Toc221317979)

[**Table B7** *The Two-factor Model for the CAQ-GE with 25 Items* 36](#_Toc221317980)

[Supplementary Material C: Sensitivity Analysis of Confirmatory Factor Analysis 40](#_Toc221317981)

[**Table C1** *Comparison of Measure Model* 40](#_Toc221317982)

[**Table C2** *The One-factor Model for the CAQ-W with 25 Items* 41](#_Toc221317983)

[**Table C3** *The Two-factor Model for the CAQ-W with 25 Items* 43](#_Toc221317984)

[**Table C4** *The Three-factor Model for the CAQ-W with 25 Items* 46](#_Toc221317985)

[**Table C5** *The Three-factor Model for the CAQ-W with 30 Items* 49](#_Toc221317986)

[**Table C6** *The One-factor Model for the CAQ-GE with 24 Items* 52](#_Toc221317987)

[**Table C7** *The Two-factor Model for the CAQ-GE with 24 Items* 54](#_Toc221317988)

[**Table C8** *The Two-factor Model for the CAQ-GE with 25 Items* 56](#_Toc221317989)

[Supplementary Material D: Items of CAQ Scale 58](#_Toc221317990)

[Supplementary Material E: Validity Analysis 65](#_Toc221317991)

[**Table E1** *Item Redundancy of the CAQ-W* 65](#_Toc221317992)

[**Table E2** *Item Redundancy of the CAQ-GE* 66](#_Toc221317993)

[**Table E3** *CAQ Scales and Subscales: GAD and Non-Anxious Participant Means, t-scores, and Effect Sizes* 67](#_Toc221317994)

[**Table E4** *Classification Accuracy for CAQ-W and CAQ-GE Cut Scores* 68](#_Toc221317995)

[**Figure E1** *The ROC Curve for the CAQ-W* 69](#_Toc221317996)

[**Figure E2** *The ROC Curve for the CAQ-GE* 70](#_Toc221317997)

**Supplementary Material A: Exploratory Factor Analysis**

**Table A1***The First Exploratory Factor Analysis for the CAQ-W*

|  | Confidence Intervals of Factor Loadings | | | |  | |  | |
| --- | --- | --- | --- | --- | --- | --- | --- | --- |
| Item | F1 | F2 | F3 | Communality | | KMO | |  |
| CAQ_W17 | (0.83, 0.93) | (0.06, 0.19) | (-0.27, -0.13) | 0.71 | | 0.94 | |  |
| CAQ_W1 | (0.76, 0.88) | (-0.08, 0.08) | (-0.14, 0.05) | 0.64 | | 0.94 | |  |
| CAQ_W20 | (0.75, 0.88) | (-0.17, -0.02) | (-0.17, 0.04) | 0.59 | | 0.94 | |  |
| CAQ_W16 | (0.72, 0.85) | (-0.20, -0.04) | (-0.06, 0.12) | 0.6 | | 0.93 | |  |
| CAQ_W27 | (0.72, 0.85) | (-0.05, 0.10) | (-0.08, 0.10) | 0.64 | | 0.95 | |  |
| CAQ_W15 | (0.71, 0.84) | (-0.02, 0.12) | (-0.06, 0.14) | 0.66 | | 0.9 | |  |
| CAQ_W24 | (0.71, 0.84) | (0.02, 0.16) | (-0.09, 0.10) | 0.65 | | 0.95 | |  |
| CAQ_W2 | (0.70, 0.83) | (-0.27, -0.11) | (-0.10, 0.12) | 0.56 | | 0.91 | |  |
| CAQ_W19 | (0.69, 0.82) | (-0.05, 0.10) | (0.00, 0.19) | 0.66 | | 0.95 | |  |
| CAQ_W10 | (0.68, 0.81) | (0.04, 0.18) | (-0.06, 0.12) | 0.64 | | 0.91 | |  |
| CAQ_W9 | (0.58, 0.72) | (0.01, 0.16) | (0.09, 0.28) | 0.61 | | 0.96 | |  |
| CAQ_W12 | (0.56, 0.71) | (0.01, 0.16) | (0.13, 0.32) | 0.63 | | 0.95 | |  |
| CAQ_W23 | (0.56, 0.70) | (-0.03, 0.12) | (0.16, 0.34) | 0.62 | | 0.97 | |  |
| CAQ_W30 | (0.48, 0.64) | (-0.11, 0.05) | (0.22, 0.43) | 0.55 | | 0.94 | |  |
| CAQ_W26 | (0.34, 0.55) | (-0.37, -0.17) | (0.14, 0.40) | 0.33 | | 0.91 | |  |
| CAQ_W22 | (0.34, 0.50) | (0.08, 0.23) | (0.30, 0.52) | 0.61 | | 0.97 | |  |
| CAQ_W29 | (-0.14, 0.02) | (0.79, 0.90) | (-0.20, 0.01) | 0.65 | | 0.84 | |  |
| CAQ_W8 | (-0.10, 0.04) | (0.79, 0.89) | (-0.06, 0.10) | 0.71 | | 0.93 | |  |
| CAQ_W28 | (-0.15, 0.02) | (0.74, 0.86) | (-0.26, -0.02) | 0.56 | | 0.82 | |  |
| CAQ_W13 | (0.09, 0.22) | (0.74, 0.85) | (-0.06, 0.10) | 0.74 | | 0.95 | |  |
| CAQ_W21 | (0.06, 0.21) | (0.71, 0.84) | (-0.17, 0.02) | 0.62 | | 0.93 | |  |
| CAQ_W7 | (-0.04, 0.09) | (0.70, 0.81) | (0.12, 0.27) | 0.73 | | 0.93 | |  |
| CAQ_W18 | (0.14, 0.28) | (0.69, 0.81) | (-0.06, 0.11) | 0.71 | | 0.95 | |  |
| CAQ_W5 | (-0.21, -0.07) | (0.69, 0.81) | (0.16, 0.34) | 0.7 | | 0.91 | |  |
| CAQ_W4 | (-0.16, 0.00) | (0.63, 0.77) | (0.13, 0.32) | 0.63 | | 0.91 | |  |
| CAQ_W6 | (-0.14, 0.01) | (0.16, 0.29) | (0.63, 0.82) | 0.66 | | 0.9 | |  |
| CAQ_W3 | (0.05, 0.20) | (-0.10, 0.06) | (0.58, 0.79) | 0.55 | | 0.91 | |  |
| CAQ_W14 | (0.15, 0.30) | (0.12, 0.27) | (0.46, 0.69) | 0.64 | | 0.92 | |  |
| CAQ_W25 | (0.23, 0.41) | (-0.04, 0.12) | (0.39, 0.64) | 0.53 | | 0.93 | |  |
| CAQ_W11 | (0.34, 0.50) | (0.05, 0.20) | (0.34, 0.57) | 0.63 | | 0.94 | |  |

**Table A2***The Second Exploratory Factor Analysis for the CAQ-W*

|  | Confidence Intervals of Factor Loadings | | | |  | |  | |
| --- | --- | --- | --- | --- | --- | --- | --- | --- |
| Item | F1 | F2 | F3 | Communality | | KMO | |  |
| CAQ_W17 | (0.82, 0.92) | (0.06, 0.18) | (-0.28, -0.12) | 0.72 | | 0.95 | |  |
| CAQ_W1 | (0.76, 0.87) | (-0.08, 0.07) | (-0.14, 0.04) | 0.64 | | 0.94 | |  |
| CAQ_W20 | (0.75, 0.86) | (-0.17, -0.02) | (-0.15, 0.04) | 0.59 | | 0.94 | |  |
| CAQ_W16 | (0.73, 0.84) | (-0.20, -0.04) | (-0.07, 0.12) | 0.6 | | 0.92 | |  |
| CAQ_W27 | (0.73, 0.85) | (-0.05, 0.09) | (-0.08, 0.09) | 0.64 | | 0.95 | |  |
| CAQ_W15 | (0.73, 0.84) | (-0.04, 0.12) | (-0.05, 0.14) | 0.67 | | 0.89 | |  |
| CAQ_W24 | (0.72, 0.84) | (0.01, 0.16) | (-0.08, 0.10) | 0.65 | | 0.95 | |  |
| CAQ_W2 | (0.70, 0.83) | (-0.27, -0.11) | (-0.09, 0.11) | 0.55 | | 0.9 | |  |
| CAQ_W10 | (0.69, 0.81) | (0.03, 0.18) | (-0.07, 0.11) | 0.64 | | 0.9 | |  |
| CAQ_W19 | (0.70, 0.82) | (-0.04, 0.10) | (-0.00, 0.16) | 0.65 | | 0.95 | |  |
| CAQ_W9 | (0.60, 0.73) | (0.01, 0.16) | (0.07, 0.26) | 0.6 | | 0.95 | |  |
| CAQ_W12 | (0.59, 0.72) | (0.01, 0.16) | (0.12, 0.29) | 0.63 | | 0.95 | |  |
| CAQ_W23 | (0.58, 0.71) | (-0.03, 0.12) | (0.15, 0.33) | 0.62 | | 0.96 | |  |
| CAQ_W30 | (0.50, 0.66) | (-0.12, 0.04) | (0.21, 0.42) | 0.56 | | 0.94 | |  |
| CAQ_W29 | (-0.13, 0.01) | (0.81, 0.91) | (-0.21, -0.00) | 0.66 | | 0.86 | |  |
| CAQ_W8 | (-0.09, 0.03) | (0.79, 0.89) | (-0.05, 0.11) | 0.72 | | 0.93 | |  |
| CAQ_W28 | (-0.15, 0.01) | (0.76, 0.88) | (-0.28, -0.05) | 0.58 | | 0.82 | |  |
| CAQ_W13 | (0.10, 0.22) | (0.73, 0.84) | (-0.05, 0.11) | 0.73 | | 0.94 | |  |
| CAQ_W21 | (0.06, 0.20) | (0.70, 0.82) | (-0.14, 0.04) | 0.61 | | 0.93 | |  |
| CAQ_W5 | (-0.19, -0.05) | (0.69, 0.81) | (0.16, 0.32) | 0.7 | | 0.9 | |  |
| CAQ_W7 | (-0.03, 0.09) | (0.69, 0.81) | (0.13, 0.28) | 0.74 | | 0.93 | |  |
| CAQ_W18 | (0.15, 0.28) | (0.69, 0.81) | (-0.07, 0.10) | 0.7 | | 0.95 | |  |
| CAQ_W4 | (-0.13, 0.01) | (0.63, 0.77) | (0.13, 0.32) | 0.63 | | 0.91 | |  |
| CAQ_W6 | (-0.09, 0.05) | (0.15, 0.28) | (0.63, 0.82) | 0.68 | | 0.87 | |  |
| CAQ_W3 | (0.10, 0.25) | (-0.10, 0.06) | (0.55, 0.78) | 0.55 | | 0.91 | |  |
| CAQ_W14 | (0.20, 0.34) | (0.12, 0.26) | (0.45, 0.64) | 0.62 | | 0.94 | |  |
| CAQ_W25 | (0.27, 0.44) | (-0.03, 0.13) | (0.35, 0.60) | 0.51 | | 0.93 | |  |

**Table A3***The Third Exploratory Factor Analysis for the CAQ-W*

|  | Confidence Intervals of Factor Loadings | | | |  | |  | |
| --- | --- | --- | --- | --- | --- | --- | --- | --- |
| Item | F1 | F2 | F3 | Communality | | KMO | |  |
| CAQ_W17 | (0.81, 0.91) | (0.06, 0.19) | (-0.28, -0.12) | 0.71 | | 0.95 | |  |
| CAQ_W1 | (0.77, 0.87) | (-0.08, 0.06) | (-0.14, 0.04) | 0.64 | | 0.95 | |  |
| CAQ_W20 | (0.75, 0.86) | (-0.17, -0.02) | (-0.17, 0.03) | 0.59 | | 0.94 | |  |
| CAQ_W15 | (0.73, 0.84) | (-0.05, 0.10) | (-0.03, 0.16) | 0.67 | | 0.89 | |  |
| CAQ_W16 | (0.73, 0.84) | (-0.20, -0.05) | (-0.07, 0.11) | 0.6 | | 0.92 | |  |
| CAQ_W27 | (0.74, 0.85) | (-0.06, 0.09) | (-0.08, 0.09) | 0.64 | | 0.95 | |  |
| CAQ_W2 | (0.71, 0.84) | (-0.27, -0.10) | (-0.11, 0.09) | 0.55 | | 0.9 | |  |
| CAQ_W19 | (0.72, 0.83) | (-0.04, 0.11) | (-0.03, 0.14) | 0.65 | | 0.95 | |  |
| CAQ_W24 | (0.73, 0.83) | (0.00, 0.15) | (-0.08, 0.10) | 0.65 | | 0.94 | |  |
| CAQ_W10 | (0.70, 0.82) | (0.03, 0.18) | (-0.08, 0.11) | 0.64 | | 0.9 | |  |
| CAQ_W9 | (0.61, 0.74) | (-0.00, 0.15) | (0.07, 0.26) | 0.61 | | 0.95 | |  |
| CAQ_W12 | (0.61, 0.74) | (0.01, 0.16) | (0.09, 0.27) | 0.62 | | 0.95 | |  |
| CAQ_W23 | (0.61, 0.74) | (-0.03, 0.12) | (0.11, 0.29) | 0.61 | | 0.97 | |  |
| CAQ_W30 | (0.54, 0.68) | (-0.11, 0.05) | (0.17, 0.36) | 0.54 | | 0.96 | |  |
| CAQ_W29 | (-0.13, 0.00) | (0.82, 0.92) | (-0.22, -0.03) | 0.67 | | 0.85 | |  |
| CAQ_W28 | (-0.14, 0.00) | (0.79, 0.90) | (-0.30, -0.08) | 0.61 | | 0.82 | |  |
| CAQ_W8 | (-0.10, 0.03) | (0.77, 0.88) | (-0.02, 0.14) | 0.71 | | 0.93 | |  |
| CAQ_W13 | (0.10, 0.22) | (0.71, 0.83) | (-0.03, 0.15) | 0.73 | | 0.94 | |  |
| CAQ_W21 | (0.06, 0.20) | (0.69, 0.82) | (-0.14, 0.06) | 0.61 | | 0.93 | |  |
| CAQ_W18 | (0.15, 0.28) | (0.68, 0.80) | (-0.06, 0.11) | 0.7 | | 0.95 | |  |
| CAQ_W5 | (-0.17, -0.04) | (0.66, 0.80) | (0.18, 0.35) | 0.7 | | 0.9 | |  |
| CAQ_W7 | (-0.02, 0.10) | (0.67, 0.80) | (0.15, 0.30) | 0.74 | | 0.92 | |  |
| CAQ_W4 | (-0.12, 0.02) | (0.61, 0.75) | (0.16, 0.34) | 0.63 | | 0.91 | |  |
| CAQ_W6 | (-0.04, 0.07) | (0.12, 0.24) | (0.66, 0.86) | 0.74 | | 0.86 | |  |
| CAQ_W3 | (0.15, 0.31) | (-0.10, 0.06) | (0.50, 0.73) | 0.51 | | 0.89 | |  |
| CAQ_W14 | (0.24, 0.37) | (0.11, 0.24) | (0.43, 0.62) | 0.62 | | 0.93 | |  |

**Table A4***The Fourth Exploratory Factor Analysis for the CAQ-W*

|  | Confidence Intervals of Factor Loadings | | | |  | |  | |
| --- | --- | --- | --- | --- | --- | --- | --- | --- |
| Item | F1 | F2 | F3 | Communality | | KMO | |  |
| CAQ_W17 | (0.81, 0.90) | (0.06, 0.19) | (-0.27, -0.12) | 0.71 | | 0.94 | |  |
| CAQ_W1 | (0.76, 0.87) | (-0.08, 0.06) | (-0.14, 0.04) | 0.64 | | 0.95 | |  |
| CAQ_W20 | (0.75, 0.86) | (-0.18, -0.02) | (-0.16, 0.04) | 0.6 | | 0.94 | |  |
| CAQ_W15 | (0.73, 0.84) | (-0.06, 0.09) | (-0.02, 0.17) | 0.67 | | 0.89 | |  |
| CAQ_W16 | (0.73, 0.84) | (-0.20, -0.05) | (-0.06, 0.13) | 0.6 | | 0.92 | |  |
| CAQ_W27 | (0.73, 0.84) | (-0.05, 0.10) | (-0.09, 0.09) | 0.63 | | 0.95 | |  |
| CAQ_W24 | (0.73, 0.83) | (-0.00, 0.15) | (-0.07, 0.11) | 0.66 | | 0.94 | |  |
| CAQ_W2 | (0.71, 0.83) | (-0.27, -0.10) | (-0.11, 0.10) | 0.55 | | 0.89 | |  |
| CAQ_W19 | (0.72, 0.83) | (-0.04, 0.11) | (-0.04, 0.14) | 0.64 | | 0.95 | |  |
| CAQ_W10 | (0.70, 0.81) | (0.02, 0.18) | (-0.07, 0.12) | 0.63 | | 0.9 | |  |
| CAQ_W9 | (0.61, 0.74) | (-0.01, 0.15) | (0.08, 0.26) | 0.61 | | 0.95 | |  |
| CAQ_W12 | (0.61, 0.74) | (0.00, 0.15) | (0.09, 0.28) | 0.62 | | 0.95 | |  |
| CAQ_W23 | (0.61, 0.73) | (-0.04, 0.11) | (0.12, 0.30) | 0.62 | | 0.97 | |  |
| CAQ_W29 | (-0.13, 0.01) | (0.83, 0.93) | (-0.23, -0.03) | 0.68 | | 0.85 | |  |
| CAQ_W28 | (-0.14, -0.00) | (0.79, 0.91) | (-0.31, -0.09) | 0.61 | | 0.82 | |  |
| CAQ_W8 | (-0.09, 0.03) | (0.76, 0.88) | (-0.01, 0.15) | 0.71 | | 0.93 | |  |
| CAQ_W13 | (0.10, 0.23) | (0.69, 0.82) | (-0.02, 0.16) | 0.73 | | 0.94 | |  |
| CAQ_W21 | (0.06, 0.20) | (0.68, 0.82) | (-0.13, 0.07) | 0.61 | | 0.93 | |  |
| CAQ_W18 | (0.15, 0.28) | (0.67, 0.80) | (-0.05, 0.12) | 0.7 | | 0.95 | |  |
| CAQ_W7 | (-0.02, 0.10) | (0.66, 0.79) | (0.16, 0.31) | 0.74 | | 0.93 | |  |
| CAQ_W5 | (-0.17, -0.04) | (0.65, 0.79) | (0.19, 0.37) | 0.7 | | 0.91 | |  |
| CAQ_W4 | (-0.12, 0.02) | (0.59, 0.75) | (0.17, 0.37) | 0.64 | | 0.9 | |  |
| CAQ_W6 | (-0.04, 0.07) | (0.10, 0.23) | (0.66, 0.88) | 0.73 | | 0.85 | |  |
| CAQ_W3 | (0.15, 0.30) | (-0.11, 0.05) | (0.49, 0.72) | 0.49 | | 0.89 | |  |
| CAQ_W14 | (0.24, 0.37) | (0.09, 0.24) | (0.43, 0.63) | 0.62 | | 0.93 | |  |

**Table A5***The Two-factor Solution in the First Exploratory Factor Analysis for the CAQ-GE*

|  | Confidence Intervals of Factor Loadings | |  | |  | |
| --- | --- | --- | --- | --- | --- | --- |
| Item | F1 | F2 | Communality | KMO | |  |
| CAQ_GE18 | (0.78, 0.90) | (-0.12, 0.05) | 0.68 | 0.95 | |  |
| CAQ_GE5 | (0.76, 0.88) | (-0.04, 0.12) | 0.71 | 0.91 | |  |
| CAQ_GE7 | (0.76, 0.88) | (-0.06, 0.11) | 0.7 | 0.94 | |  |
| CAQ_GE17 | (0.73, 0.88) | (-0.21, -0.01) | 0.58 | 0.92 | |  |
| CAQ_GE8 | (0.74, 0.86) | (-0.04, 0.13) | 0.69 | 0.95 | |  |
| CAQ_GE20 | (0.72, 0.85) | (-0.05, 0.11) | 0.65 | 0.94 | |  |
| CAQ_GE9 | (0.71, 0.85) | (-0.17, 0.01) | 0.55 | 0.95 | |  |
| CAQ_GE12 | (0.71, 0.84) | (-0.02, 0.14) | 0.66 | 0.95 | |  |
| CAQ_GE24 | (0.71, 0.83) | (-0.02, 0.14) | 0.65 | 0.95 | |  |
| CAQ_GE2 | (0.67, 0.85) | (-0.20, 0.03) | 0.52 | 0.91 | |  |
| CAQ_GE10 | (0.69, 0.83) | (-0.18, 0.01) | 0.52 | 0.96 | |  |
| CAQ_GE25 | (0.68, 0.83) | (-0.05, 0.13) | 0.61 | 0.96 | |  |
| CAQ_GE16 | (0.64, 0.80) | (-0.10, 0.10) | 0.53 | 0.95 | |  |
| CAQ_GE4 | (0.63, 0.79) | (-0.03, 0.17) | 0.57 | 0.91 | |  |
| CAQ_GE22 | (0.52, 0.71) | (0.03, 0.25) | 0.49 | 0.87 | |  |
| CAQ_GE21 | (0.51, 0.70) | (0.04, 0.26) | 0.49 | 0.92 | |  |
| CAQ_GE1 | (0.42, 0.64) | (-0.05, 0.21) | 0.33 | 0.83 | |  |
| CAQ_GE13 | (0.24, 0.49) | (-0.11, 0.15) | 0.14 | 0.91 | |  |
| CAQ_GE19 | (-0.16, -0.03) | (0.90, 0.99) | 0.8 | 0.88 | |  |
| CAQ_GE11 | (-0.07, 0.08) | (0.72, 0.86) | 0.63 | 0.92 | |  |
| CAQ_GE15 | (0.02, 0.17) | (0.69, 0.83) | 0.67 | 0.93 | |  |
| CAQ_GE23 | (-0.05, 0.11) | (0.69, 0.84) | 0.61 | 0.92 | |  |
| CAQ_GE6 | (0.03, 0.20) | (0.60, 0.79) | 0.59 | 0.91 | |  |
| CAQ_GE3 | (0.15, 0.33) | (0.46, 0.66) | 0.51 | 0.95 | |  |
| CAQ_GE14 | (-0.01, 0.21) | (0.39, 0.62) | 0.32 | 0.87 | |  |

**Table A6***The Three-factor Solution in the First Exploratory Factor Analysis for the CAQ-GE*

|  | Confidence Intervals of Factor Loadings | | | |  | |  | |
| --- | --- | --- | --- | --- | --- | --- | --- | --- |
| Item | F1 | F2 | F3 | Communality | | KMO | |  |
| CAQ_GE17 | (0.70, 0.96) | (-0.18, -0.02) | (-0.27, 0.04) | 0.64 | | 0.92 | |  |
| CAQ_GE18 | (0.69, 0.99) | (-0.09, 0.05) | (-0.20, 0.06) | 0.73 | | 0.95 | |  |
| CAQ_GE16 | (0.62, 0.89) | (-0.08, 0.10) | (-0.29, 0.04) | 0.59 | | 0.95 | |  |
| CAQ_GE25 | (0.59, 0.86) | (-0.02, 0.14) | (-0.14, 0.12) | 0.63 | | 0.96 | |  |
| CAQ_GE24 | (0.57, 0.85) | (0.01, 0.16) | (-0.06, 0.16) | 0.65 | | 0.95 | |  |
| CAQ_GE20 | (0.56, 0.83) | (-0.02, 0.14) | (-0.02, 0.26) | 0.64 | | 0.94 | |  |
| CAQ_GE21 | (0.55, 0.81) | (0.06, 0.23) | (-0.42, -0.03) | 0.59 | | 0.92 | |  |
| CAQ_GE22 | (0.55, 0.81) | (0.05, 0.23) | (-0.37, -0.03) | 0.58 | | 0.87 | |  |
| CAQ_GE10 | (0.51, 0.82) | (-0.14, 0.04) | (-0.02, 0.29) | 0.52 | | 0.96 | |  |
| CAQ_GE9 | (0.49, 0.82) | (-0.13, 0.05) | (0.01, 0.38) | 0.55 | | 0.95 | |  |
| CAQ_GE12 | (0.48, 0.80) | (0.02, 0.18) | (0.03, 0.39) | 0.65 | | 0.95 | |  |
| CAQ_GE5 | (0.45, 0.77) | (0.02, 0.17) | (0.16, 0.62) | 0.74 | | 0.91 | |  |
| CAQ_GE7 | (0.45, 0.75) | (0.01, 0.15) | (0.19, 0.63) | 0.74 | | 0.94 | |  |
| CAQ_GE8 | (0.45, 0.72) | (0.03, 0.17) | (0.23, 0.58) | 0.73 | | 0.95 | |  |
| CAQ_GE4 | (0.37, 0.63) | (0.04, 0.21) | (0.20, 0.59) | 0.61 | | 0.91 | |  |
| CAQ_GE2 | (0.36, 0.60) | (-0.11, 0.06) | (0.31, 0.83) | 0.64 | | 0.91 | |  |
| CAQ_GE13 | (0.16, 0.50) | (-0.09, 0.16) | (-0.19, 0.27) | 0.14 | | 0.91 | |  |
| CAQ_GE19 | (-0.09, 0.04) | (0.88, 0.97) | (-0.28, -0.08) | 0.83 | | 0.88 | |  |
| CAQ_GE11 | (-0.10, 0.07) | (0.72, 0.86) | (-0.10, 0.17) | 0.63 | | 0.92 | |  |
| CAQ_GE15 | (0.02, 0.18) | (0.69, 0.83) | (-0.14, 0.06) | 0.67 | | 0.93 | |  |
| CAQ_GE23 | (-0.03, 0.16) | (0.68, 0.83) | (-0.24, 0.05) | 0.62 | | 0.92 | |  |
| CAQ_GE6 | (-0.14, 0.05) | (0.66, 0.81) | (0.15, 0.47) | 0.65 | | 0.91 | |  |
| CAQ_GE3 | (0.03, 0.24) | (0.49, 0.67) | (0.04, 0.34) | 0.53 | | 0.95 | |  |
| CAQ_GE14 | (-0.11, 0.12) | (0.42, 0.62) | (0.00, 0.36) | 0.33 | | 0.87 | |  |
| CAQ_GE1 | (0.12, 0.36) | (0.05, 0.24) | (0.32, 0.87) | 0.49 | | 0.83 | |  |

**Table A7***The Four-factor Solution in the First Exploratory Factor Analysis for the CAQ-GE*

|  | Confidence Intervals of Factor Loadings | | | | |  | |  | |
| --- | --- | --- | --- | --- | --- | --- | --- | --- | --- |
| Item | F1 | F2 | F3 | F4 | Communality | | KMO | |  |
| CAQ_GE18 | (0.61, 1.05) | (-0.06, 0.07) | (-0.33, 0.19) | (-0.09, 0.33) | 0.75 | | 0.95 | |  |
| CAQ_GE17 | (0.58, 1.02) | (-0.16, -0.01) | (-0.16, 0.18) | (-0.36, 0.17) | 0.64 | | 0.92 | |  |
| CAQ_GE16 | (0.51, 0.95) | (-0.07, 0.10) | (-0.34, 0.38) | (-0.61, 0.30) | 0.6 | | 0.95 | |  |
| CAQ_GE21 | (0.48, 0.90) | (0.08, 0.23) | (-0.47, 0.36) | (-0.84, 0.30) | 0.62 | | 0.92 | |  |
| CAQ_GE22 | (0.48, 0.89) | (0.06, 0.23) | (-0.43, 0.31) | (-0.72, 0.30) | 0.59 | | 0.87 | |  |
| CAQ_GE25 | (0.47, 0.88) | (-0.01, 0.15) | (-0.33, 0.59) | (-0.55, 0.47) | 0.64 | | 0.96 | |  |
| CAQ_GE24 | (0.45, 0.85) | (0.02, 0.16) | (-0.26, 0.52) | (-0.26, 0.40) | 0.65 | | 0.95 | |  |
| CAQ_GE10 | (0.40, 0.79) | (-0.12, 0.05) | (-0.50, 0.81) | (-0.40, 0.80) | 0.52 | | 0.96 | |  |
| CAQ_GE9 | (0.41, 0.77) | (-0.11, 0.06) | (-0.63, 0.92) | (-0.32, 0.98) | 0.58 | | 0.95 | |  |
| CAQ_GE12 | (0.42, 0.74) | (0.04, 0.18) | (-0.84, 1.16) | (-0.53, 1.25) | 0.69 | | 0.95 | |  |
| CAQ_GE20 | (0.40, 0.79) | (-0.02, 0.14) | (-0.26, 0.82) | (-0.55, 0.56) | 0.66 | | 0.94 | |  |
| CAQ_GE5 | (0.36, 0.64) | (0.04, 0.17) | (-1.18, 1.82) | (-0.87, 1.90) | 0.8 | | 0.91 | |  |
| CAQ_GE7 | (0.34, 0.61) | (0.02, 0.15) | (-1.09, 1.83) | (-0.87, 1.86) | 0.77 | | 0.94 | |  |
| CAQ_GE8 | (0.28, 0.59) | (0.03, 0.16) | (-0.87, 1.82) | (-1.01, 1.65) | 0.72 | | 0.95 | |  |
| CAQ_GE13 | (0.21, 0.50) | (-0.04, 0.16) | (-0.60, 0.35) | (-0.06, 0.71) | 0.22 | | 0.91 | |  |
| CAQ_GE4 | (0.22, 0.50) | (0.03, 0.20) | (-0.90, 1.82) | (-1.04, 1.63) | 0.6 | | 0.91 | |  |
| CAQ_GE19 | (-0.03, 0.10) | (0.83, 1.01) | (-0.67, 0.43) | (-0.83, 0.45) | 0.83 | | 0.88 | |  |
| CAQ_GE11 | (-0.07, 0.10) | (0.71, 0.88) | (-0.36, 0.26) | (-0.01, 0.35) | 0.66 | | 0.92 | |  |
| CAQ_GE15 | (0.03, 0.21) | (0.66, 0.84) | (-0.22, 0.18) | (-0.24, 0.21) | 0.67 | | 0.93 | |  |
| CAQ_GE23 | (-0.03, 0.18) | (0.66, 0.84) | (-0.30, 0.38) | (-0.67, 0.18) | 0.66 | | 0.92 | |  |
| CAQ_GE6 | (-0.19, -0.00) | (0.64, 0.82) | (-0.85, 1.34) | (-0.69, 1.38) | 0.68 | | 0.91 | |  |
| CAQ_GE3 | (-0.05, 0.19) | (0.46, 0.67) | (-0.38, 0.90) | (-0.53, 0.74) | 0.52 | | 0.95 | |  |
| CAQ_GE14 | (-0.18, 0.09) | (0.39, 0.63) | (-0.52, 0.97) | (-0.67, 0.84) | 0.33 | | 0.87 | |  |
| CAQ_GE1 | (-0.16, 0.09) | (0.03, 0.17) | (-0.84, 2.65) | (-1.75, 2.05) | 0.66 | | 0.83 | |  |
| CAQ_GE2 | (0.08, 0.35) | (-0.13, -0.01) | (-0.86, 2.60) | (-1.70, 2.03) | 0.77 | | 0.91 | |  |

**Table A8***The Two-factor Solution in the Second Exploratory Factor Analysis for the CAQ-GE*

|  | Confidence Intervals of Factor Loadings | |  | |  | |
| --- | --- | --- | --- | --- | --- | --- |
| Item | F1 | F2 | Communality | KMO | |  |
| CAQ_GE18 | (0.78, 0.90) | (-0.12, 0.06) | 0.68 | 0.95 | |  |
| CAQ_GE5 | (0.75, 0.88) | (-0.04, 0.13) | 0.71 | 0.9 | |  |
| CAQ_GE7 | (0.75, 0.88) | (-0.06, 0.12) | 0.69 | 0.94 | |  |
| CAQ_GE8 | (0.74, 0.86) | (-0.04, 0.13) | 0.69 | 0.94 | |  |
| CAQ_GE17 | (0.74, 0.88) | (-0.21, -0.01) | 0.58 | 0.92 | |  |
| CAQ_GE20 | (0.73, 0.85) | (-0.06, 0.11) | 0.65 | 0.94 | |  |
| CAQ_GE9 | (0.71, 0.84) | (-0.17, 0.02) | 0.55 | 0.95 | |  |
| CAQ_GE2 | (0.68, 0.85) | (-0.20, 0.03) | 0.52 | 0.91 | |  |
| CAQ_GE12 | (0.71, 0.83) | (-0.02, 0.14) | 0.65 | 0.95 | |  |
| CAQ_GE24 | (0.71, 0.83) | (-0.03, 0.14) | 0.65 | 0.95 | |  |
| CAQ_GE10 | (0.69, 0.83) | (-0.17, 0.01) | 0.52 | 0.96 | |  |
| CAQ_GE25 | (0.69, 0.83) | (-0.05, 0.13) | 0.62 | 0.96 | |  |
| CAQ_GE16 | (0.64, 0.80) | (-0.10, 0.10) | 0.52 | 0.95 | |  |
| CAQ_GE4 | (0.63, 0.79) | (-0.03, 0.17) | 0.57 | 0.9 | |  |
| CAQ_GE22 | (0.53, 0.71) | (0.03, 0.25) | 0.49 | 0.87 | |  |
| CAQ_GE21 | (0.51, 0.70) | (0.04, 0.26) | 0.49 | 0.92 | |  |
| CAQ_GE1 | (0.42, 0.64) | (-0.05, 0.22) | 0.34 | 0.83 | |  |
| CAQ_GE19 | (-0.16, -0.03) | (0.89, 0.99) | 0.8 | 0.88 | |  |
| CAQ_GE11 | (-0.07, 0.07) | (0.73, 0.86) | 0.63 | 0.92 | |  |
| CAQ_GE23 | (-0.04, 0.12) | (0.69, 0.84) | 0.61 | 0.93 | |  |
| CAQ_GE15 | (0.02, 0.17) | (0.69, 0.83) | 0.67 | 0.93 | |  |
| CAQ_GE6 | (0.03, 0.20) | (0.61, 0.79) | 0.58 | 0.91 | |  |
| CAQ_GE3 | (0.16, 0.34) | (0.46, 0.65) | 0.52 | 0.96 | |  |
| CAQ_GE14 | (-0.02, 0.20) | (0.40, 0.62) | 0.32 | 0.87 | |  |

**Figure A1***The Scree Plot of the Initial Exploratory Factor Analysis for the CAQ-W*

*
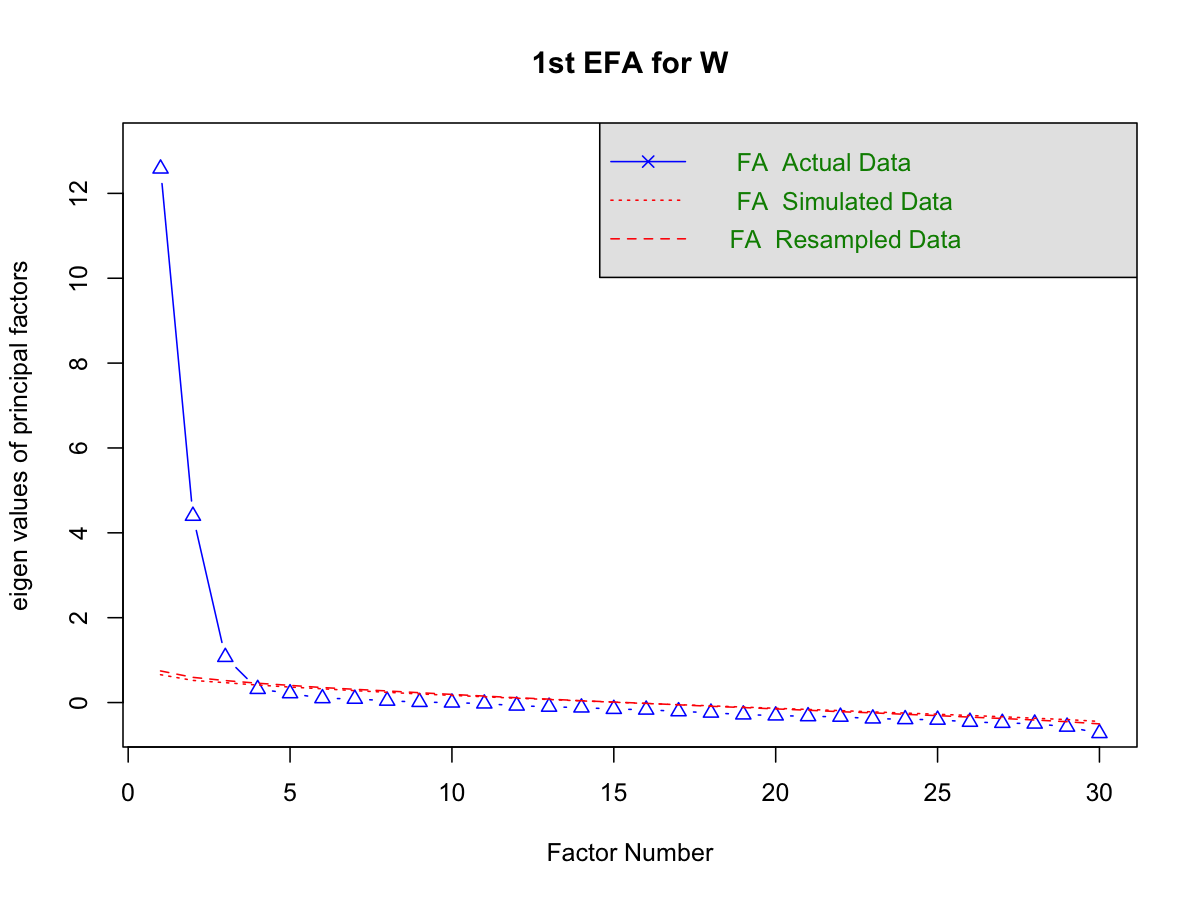
*

**Figure A2***The Scree Plot of the Initial Exploratory Factor Analysis for the CAQ-GE*

*
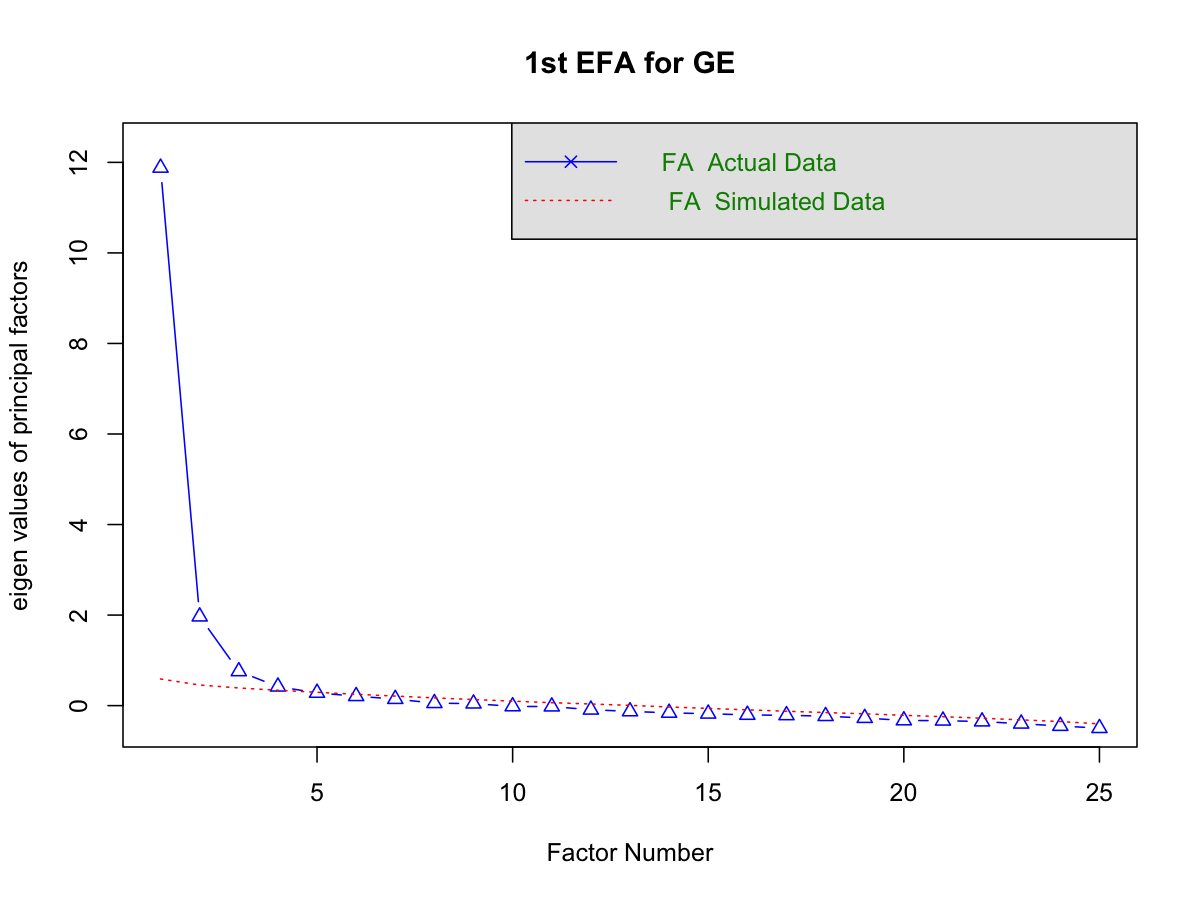
*

**Supplementary Material B: Confirmatory Factor Analysis**

**Table B1***The One-factor Model for the CAQ-W with 25 Items*

| Parameter Type | Item/Factor | Estimate | SE |
| --- | --- | --- | --- |
| F1.BY | W9 | 0.72 | 0.03 |
| F1.BY | W10 | 0.79 | 0.02 |
| F1.BY | W17 | 0.79 | 0.02 |
| F1.BY | W15 | 0.80 | 0.02 |
| F1.BY | W1 | 0.72 | 0.02 |
| F1.BY | W2 | 0.61 | 0.03 |
| F1.BY | W12 | 0.76 | 0.02 |
| F1.BY | W16 | 0.63 | 0.03 |
| F1.BY | W19 | 0.79 | 0.02 |
| F1.BY | W20 | 0.70 | 0.03 |
| F1.BY | W23 | 0.72 | 0.02 |
| F1.BY | W24 | 0.77 | 0.02 |
| F1.BY | W27 | 0.75 | 0.02 |
| F1.BY | W6 | 0.57 | 0.04 |
| F1.BY | W3 | 0.61 | 0.03 |
| F1.BY | W14 | 0.61 | 0.03 |
| F1.BY | W5 | 0.72 | 0.02 |
| F1.BY | W8 | 0.73 | 0.02 |
| F1.BY | W13 | 0.76 | 0.02 |
| F1.BY | W29 | 0.69 | 0.03 |
| F1.BY | W18 | 0.69 | 0.03 |
| F1.BY | W4 | 0.68 | 0.02 |
| F1.BY | W7 | 0.62 | 0.03 |
| F1.BY | W21 | 0.71 | 0.02 |
| F1.BY | W28 | 0.59 | 0.03 |
| Thresholds | W1$1 | -0.59 | 0.08 |
| Thresholds | W1$2 | 0.03 | 0.07 |
| Thresholds | W1$3 | 0.64 | 0.08 |
| Thresholds | W1$4 | 1.95 | 0.15 |
| Thresholds | W2$1 | -0.64 | 0.08 |
| Thresholds | W2$2 | 0.00 | 0.07 |
| Thresholds | W2$3 | 0.90 | 0.08 |
| Thresholds | W2$4 | 1.76 | 0.13 |
| Thresholds | W3$1 | -1.49 | 0.11 |
| Thresholds | W3$2 | -0.91 | 0.08 |
| Thresholds | W3$3 | -0.11 | 0.07 |
| Thresholds | W3$4 | 1.07 | 0.09 |
| Thresholds | W4$1 | -1.63 | 0.12 |
| Thresholds | W4$2 | -0.71 | 0.08 |
| Thresholds | W4$3 | -0.02 | 0.07 |
| Thresholds | W4$4 | 1.19 | 0.09 |
| Thresholds | W5$1 | -1.69 | 0.12 |
| Thresholds | W5$2 | -0.82 | 0.08 |
| Thresholds | W5$3 | -0.03 | 0.07 |
| Thresholds | W5$4 | 1.16 | 0.09 |
| Thresholds | W6$1 | -1.95 | 0.15 |
| Thresholds | W6$2 | -1.03 | 0.09 |
| Thresholds | W6$3 | -0.25 | 0.07 |
| Thresholds | W6$4 | 0.91 | 0.08 |
| Thresholds | W7$1 | -1.80 | 0.14 |
| Thresholds | W7$2 | -0.99 | 0.09 |
| Thresholds | W7$3 | -0.15 | 0.07 |
| Thresholds | W7$4 | 1.28 | 0.10 |
| Thresholds | W8$1 | -1.40 | 0.10 |
| Thresholds | W8$2 | -0.53 | 0.08 |
| Thresholds | W8$3 | 0.15 | 0.07 |
| Thresholds | W8$4 | 1.26 | 0.10 |
| Thresholds | W9$1 | -0.96 | 0.09 |
| Thresholds | W9$2 | -0.27 | 0.07 |
| Thresholds | W9$3 | 0.37 | 0.07 |
| Thresholds | W9$4 | 1.24 | 0.10 |
| Thresholds | W10$1 | -0.85 | 0.08 |
| Thresholds | W10$2 | -0.10 | 0.07 |
| Thresholds | W10$3 | 0.52 | 0.08 |
| Thresholds | W10$4 | 1.47 | 0.11 |
| Thresholds | W12$1 | -1.57 | 0.11 |
| Thresholds | W12$2 | -0.42 | 0.07 |
| Thresholds | W12$3 | 0.35 | 0.07 |
| Thresholds | W12$4 | 1.36 | 0.10 |
| Thresholds | W13$1 | -1.42 | 0.11 |
| Thresholds | W13$2 | -0.50 | 0.08 |
| Thresholds | W13$3 | 0.35 | 0.07 |
| Thresholds | W13$4 | 1.44 | 0.11 |
| Thresholds | W14$1 | -1.73 | 0.13 |
| Thresholds | W14$2 | -0.78 | 0.08 |
| Thresholds | W14$3 | 0.06 | 0.07 |
| Thresholds | W14$4 | 1.32 | 0.10 |
| Thresholds | W15$1 | -1.13 | 0.09 |
| Thresholds | W15$2 | -0.23 | 0.07 |
| Thresholds | W15$3 | 0.40 | 0.07 |
| Thresholds | W15$4 | 1.47 | 0.11 |
| Thresholds | W16$1 | -0.89 | 0.08 |
| Thresholds | W16$2 | -0.11 | 0.07 |
| Thresholds | W16$3 | 0.67 | 0.08 |
| Thresholds | W16$4 | 1.69 | 0.12 |
| Thresholds | W17$1 | -0.86 | 0.08 |
| Thresholds | W17$2 | -0.06 | 0.07 |
| Thresholds | W17$3 | 0.53 | 0.08 |
| Thresholds | W17$4 | 1.76 | 0.13 |
| Thresholds | W18$1 | -1.30 | 0.10 |
| Thresholds | W18$2 | -0.55 | 0.08 |
| Thresholds | W18$3 | 0.37 | 0.07 |
| Thresholds | W18$4 | 1.60 | 0.12 |
| Thresholds | W19$1 | -1.06 | 0.09 |
| Thresholds | W19$2 | -0.23 | 0.07 |
| Thresholds | W19$3 | 0.40 | 0.07 |
| Thresholds | W19$4 | 1.57 | 0.11 |
| Thresholds | W20$1 | -0.84 | 0.08 |
| Thresholds | W20$2 | -0.20 | 0.07 |
| Thresholds | W20$3 | 0.60 | 0.08 |
| Thresholds | W20$4 | 1.63 | 0.12 |
| Thresholds | W21$1 | -1.13 | 0.09 |
| Thresholds | W21$2 | -0.54 | 0.08 |
| Thresholds | W21$3 | 0.33 | 0.07 |
| Thresholds | W21$4 | 1.66 | 0.12 |
| Thresholds | W23$1 | -1.57 | 0.11 |
| Thresholds | W23$2 | -0.55 | 0.08 |
| Thresholds | W23$3 | 0.32 | 0.07 |
| Thresholds | W23$4 | 1.49 | 0.11 |
| Thresholds | W24$1 | -0.71 | 0.08 |
| Thresholds | W24$2 | -0.13 | 0.07 |
| Thresholds | W24$3 | 0.50 | 0.08 |
| Thresholds | W24$4 | 1.73 | 0.13 |
| Thresholds | W27$1 | -0.64 | 0.08 |
| Thresholds | W27$2 | -0.04 | 0.07 |
| Thresholds | W27$3 | 0.59 | 0.08 |
| Thresholds | W27$4 | 1.69 | 0.12 |
| Thresholds | W28$1 | -1.36 | 0.10 |
| Thresholds | W28$2 | -0.45 | 0.07 |
| Thresholds | W28$3 | 0.29 | 0.07 |
| Thresholds | W28$4 | 1.26 | 0.10 |
| Thresholds | W29$1 | -1.54 | 0.11 |
| Thresholds | W29$2 | -0.57 | 0.08 |
| Thresholds | W29$3 | 0.27 | 0.07 |
| Thresholds | W29$4 | 1.23 | 0.10 |

*Note.* F1.BY is the loading on F1.

SE=Standard Error.

**Table B2***The Two-factor Model for the CAQ-W with 25 Items*

| Parameter Type | Item/Factor | Estimate | SE |
| --- | --- | --- | --- |
| F1.BY | W9 | 0.76 | 0.02 |
| F1.BY | W10 | 0.83 | 0.02 |
| F1.BY | W17 | 0.83 | 0.02 |
| F1.BY | W15 | 0.84 | 0.02 |
| F1.BY | W1 | 0.77 | 0.02 |
| F1.BY | W2 | 0.69 | 0.03 |
| F1.BY | W12 | 0.81 | 0.02 |
| F1.BY | W16 | 0.71 | 0.03 |
| F1.BY | W19 | 0.83 | 0.02 |
| F1.BY | W20 | 0.76 | 0.03 |
| F1.BY | W23 | 0.77 | 0.02 |
| F1.BY | W24 | 0.80 | 0.02 |
| F1.BY | W27 | 0.80 | 0.02 |
| F1.BY | W6 | 0.62 | 0.04 |
| F1.BY | W3 | 0.67 | 0.03 |
| F1.BY | W14 | 0.67 | 0.03 |
| F2.BY | W5 | 0.84 | 0.02 |
| F2.BY | W8 | 0.85 | 0.02 |
| F2.BY | W13 | 0.86 | 0.02 |
| F2.BY | W29 | 0.83 | 0.02 |
| F2.BY | W18 | 0.83 | 0.02 |
| F2.BY | W4 | 0.80 | 0.02 |
| F2.BY | W7 | 0.75 | 0.03 |
| F2.BY | W21 | 0.83 | 0.02 |
| F2.BY | W28 | 0.75 | 0.03 |
| F2.WITH | F1 | 0.36 | 0.05 |
| Thresholds | W1$1 | -0.59 | 0.08 |
| Thresholds | W1$2 | 0.03 | 0.07 |
| Thresholds | W1$3 | 0.64 | 0.08 |
| Thresholds | W1$4 | 1.95 | 0.15 |
| Thresholds | W2$1 | -0.64 | 0.08 |
| Thresholds | W2$2 | 0.00 | 0.07 |
| Thresholds | W2$3 | 0.90 | 0.08 |
| Thresholds | W2$4 | 1.76 | 0.13 |
| Thresholds | W3$1 | -1.49 | 0.11 |
| Thresholds | W3$2 | -0.91 | 0.08 |
| Thresholds | W3$3 | -0.11 | 0.07 |
| Thresholds | W3$4 | 1.07 | 0.09 |
| Thresholds | W4$1 | -1.63 | 0.12 |
| Thresholds | W4$2 | -0.71 | 0.08 |
| Thresholds | W4$3 | -0.02 | 0.07 |
| Thresholds | W4$4 | 1.19 | 0.09 |
| Thresholds | W5$1 | -1.69 | 0.12 |
| Thresholds | W5$2 | -0.82 | 0.08 |
| Thresholds | W5$3 | -0.03 | 0.07 |
| Thresholds | W5$4 | 1.16 | 0.09 |
| Thresholds | W6$1 | -1.95 | 0.15 |
| Thresholds | W6$2 | -1.03 | 0.09 |
| Thresholds | W6$3 | -0.25 | 0.07 |
| Thresholds | W6$4 | 0.91 | 0.08 |
| Thresholds | W7$1 | -1.80 | 0.14 |
| Thresholds | W7$2 | -0.99 | 0.09 |
| Thresholds | W7$3 | -0.15 | 0.07 |
| Thresholds | W7$4 | 1.28 | 0.10 |
| Thresholds | W8$1 | -1.40 | 0.10 |
| Thresholds | W8$2 | -0.53 | 0.08 |
| Thresholds | W8$3 | 0.15 | 0.07 |
| Thresholds | W8$4 | 1.26 | 0.10 |
| Thresholds | W9$1 | -0.96 | 0.09 |
| Thresholds | W9$2 | -0.27 | 0.07 |
| Thresholds | W9$3 | 0.37 | 0.07 |
| Thresholds | W9$4 | 1.24 | 0.10 |
| Thresholds | W10$1 | -0.85 | 0.08 |
| Thresholds | W10$2 | -0.10 | 0.07 |
| Thresholds | W10$3 | 0.52 | 0.08 |
| Thresholds | W10$4 | 1.47 | 0.11 |
| Thresholds | W12$1 | -1.57 | 0.11 |
| Thresholds | W12$2 | -0.42 | 0.07 |
| Thresholds | W12$3 | 0.35 | 0.07 |
| Thresholds | W12$4 | 1.36 | 0.10 |
| Thresholds | W13$1 | -1.42 | 0.11 |
| Thresholds | W13$2 | -0.50 | 0.08 |
| Thresholds | W13$3 | 0.35 | 0.07 |
| Thresholds | W13$4 | 1.44 | 0.11 |
| Thresholds | W14$1 | -1.73 | 0.13 |
| Thresholds | W14$2 | -0.78 | 0.08 |
| Thresholds | W14$3 | 0.06 | 0.07 |
| Thresholds | W14$4 | 1.32 | 0.10 |
| Thresholds | W15$1 | -1.13 | 0.09 |
| Thresholds | W15$2 | -0.23 | 0.07 |
| Thresholds | W15$3 | 0.40 | 0.07 |
| Thresholds | W15$4 | 1.47 | 0.11 |
| Thresholds | W16$1 | -0.89 | 0.08 |
| Thresholds | W16$2 | -0.11 | 0.07 |
| Thresholds | W16$3 | 0.67 | 0.08 |
| Thresholds | W16$4 | 1.69 | 0.12 |
| Thresholds | W17$1 | -0.86 | 0.08 |
| Thresholds | W17$2 | -0.06 | 0.07 |
| Thresholds | W17$3 | 0.53 | 0.08 |
| Thresholds | W17$4 | 1.76 | 0.13 |
| Thresholds | W18$1 | -1.30 | 0.10 |
| Thresholds | W18$2 | -0.55 | 0.08 |
| Thresholds | W18$3 | 0.37 | 0.07 |
| Thresholds | W18$4 | 1.60 | 0.12 |
| Thresholds | W19$1 | -1.06 | 0.09 |
| Thresholds | W19$2 | -0.23 | 0.07 |
| Thresholds | W19$3 | 0.40 | 0.07 |
| Thresholds | W19$4 | 1.57 | 0.11 |
| Thresholds | W20$1 | -0.84 | 0.08 |
| Thresholds | W20$2 | -0.20 | 0.07 |
| Thresholds | W20$3 | 0.60 | 0.08 |
| Thresholds | W20$4 | 1.63 | 0.12 |
| Thresholds | W21$1 | -1.13 | 0.09 |
| Thresholds | W21$2 | -0.54 | 0.08 |
| Thresholds | W21$3 | 0.33 | 0.07 |
| Thresholds | W21$4 | 1.66 | 0.12 |
| Thresholds | W23$1 | -1.57 | 0.11 |
| Thresholds | W23$2 | -0.55 | 0.08 |
| Thresholds | W23$3 | 0.32 | 0.07 |
| Thresholds | W23$4 | 1.49 | 0.11 |
| Thresholds | W24$1 | -0.71 | 0.08 |
| Thresholds | W24$2 | -0.13 | 0.07 |
| Thresholds | W24$3 | 0.50 | 0.08 |
| Thresholds | W24$4 | 1.73 | 0.13 |
| Thresholds | W27$1 | -0.64 | 0.08 |
| Thresholds | W27$2 | -0.04 | 0.07 |
| Thresholds | W27$3 | 0.59 | 0.08 |
| Thresholds | W27$4 | 1.69 | 0.12 |
| Thresholds | W28$1 | -1.36 | 0.10 |
| Thresholds | W28$2 | -0.45 | 0.07 |
| Thresholds | W28$3 | 0.29 | 0.07 |
| Thresholds | W28$4 | 1.26 | 0.10 |
| Thresholds | W29$1 | -1.54 | 0.11 |
| Thresholds | W29$2 | -0.57 | 0.08 |
| Thresholds | W29$3 | 0.27 | 0.07 |
| Thresholds | W29$4 | 1.23 | 0.10 |

*Note.* F1.BY is the loading on F1; F2.BY is the loading on F2.

F2.WITH is the correlation of F2 with the other factor(s).

SE=Standard Error.

**Table B3***The Three-factor Model for the CAQ-W with 25 Items*

| Parameter Type | Item/Factor | Estimate | SE |
| --- | --- | --- | --- |
| F1.BY | W9 | 0.77 | 0.02 |
| F1.BY | W10 | 0.83 | 0.02 |
| F1.BY | W17 | 0.83 | 0.02 |
| F1.BY | W15 | 0.84 | 0.02 |
| F1.BY | W1 | 0.77 | 0.02 |
| F1.BY | W2 | 0.69 | 0.03 |
| F1.BY | W12 | 0.82 | 0.02 |
| F1.BY | W16 | 0.72 | 0.03 |
| F1.BY | W19 | 0.84 | 0.02 |
| F1.BY | W20 | 0.76 | 0.03 |
| F1.BY | W23 | 0.78 | 0.02 |
| F1.BY | W24 | 0.81 | 0.02 |
| F1.BY | W27 | 0.80 | 0.02 |
| F2.BY | W5 | 0.84 | 0.02 |
| F2.BY | W8 | 0.85 | 0.02 |
| F2.BY | W13 | 0.86 | 0.02 |
| F2.BY | W29 | 0.83 | 0.02 |
| F2.BY | W18 | 0.83 | 0.02 |
| F2.BY | W4 | 0.80 | 0.02 |
| F2.BY | W7 | 0.76 | 0.03 |
| F2.BY | W21 | 0.83 | 0.02 |
| F2.BY | W28 | 0.75 | 0.03 |
| F3.BY | W6 | 0.76 | 0.03 |
| F3.BY | W3 | 0.83 | 0.03 |
| F3.BY | W14 | 0.84 | 0.03 |
| F2.WITH | F1 | 0.33 | 0.05 |
| F3.WITH | F1 | 0.68 | 0.03 |
| F3.WITH | F2 | 0.41 | 0.05 |
| Thresholds | W1$1 | -0.59 | 0.08 |
| Thresholds | W1$2 | 0.03 | 0.07 |
| Thresholds | W1$3 | 0.64 | 0.08 |
| Thresholds | W1$4 | 1.95 | 0.15 |
| Thresholds | W2$1 | -0.64 | 0.08 |
| Thresholds | W2$2 | 0.00 | 0.07 |
| Thresholds | W2$3 | 0.90 | 0.08 |
| Thresholds | W2$4 | 1.76 | 0.13 |
| Thresholds | W3$1 | -1.49 | 0.11 |
| Thresholds | W3$2 | -0.91 | 0.08 |
| Thresholds | W3$3 | -0.11 | 0.07 |
| Thresholds | W3$4 | 1.07 | 0.09 |
| Thresholds | W4$1 | -1.63 | 0.12 |
| Thresholds | W4$2 | -0.71 | 0.08 |
| Thresholds | W4$3 | -0.02 | 0.07 |
| Thresholds | W4$4 | 1.19 | 0.09 |
| Thresholds | W5$1 | -1.69 | 0.12 |
| Thresholds | W5$2 | -0.82 | 0.08 |
| Thresholds | W5$3 | -0.03 | 0.07 |
| Thresholds | W5$4 | 1.16 | 0.09 |
| Thresholds | W6$1 | -1.95 | 0.15 |
| Thresholds | W6$2 | -1.03 | 0.09 |
| Thresholds | W6$3 | -0.25 | 0.07 |
| Thresholds | W6$4 | 0.91 | 0.08 |
| Thresholds | W7$1 | -1.80 | 0.14 |
| Thresholds | W7$2 | -0.99 | 0.09 |
| Thresholds | W7$3 | -0.15 | 0.07 |
| Thresholds | W7$4 | 1.28 | 0.10 |
| Thresholds | W8$1 | -1.40 | 0.10 |
| Thresholds | W8$2 | -0.53 | 0.08 |
| Thresholds | W8$3 | 0.15 | 0.07 |
| Thresholds | W8$4 | 1.26 | 0.10 |
| Thresholds | W9$1 | -0.96 | 0.09 |
| Thresholds | W9$2 | -0.27 | 0.07 |
| Thresholds | W9$3 | 0.37 | 0.07 |
| Thresholds | W9$4 | 1.24 | 0.10 |
| Thresholds | W10$1 | -0.85 | 0.08 |
| Thresholds | W10$2 | -0.10 | 0.07 |
| Thresholds | W10$3 | 0.52 | 0.08 |
| Thresholds | W10$4 | 1.47 | 0.11 |
| Thresholds | W12$1 | -1.57 | 0.11 |
| Thresholds | W12$2 | -0.42 | 0.07 |
| Thresholds | W12$3 | 0.35 | 0.07 |
| Thresholds | W12$4 | 1.36 | 0.10 |
| Thresholds | W13$1 | -1.42 | 0.11 |
| Thresholds | W13$2 | -0.50 | 0.08 |
| Thresholds | W13$3 | 0.35 | 0.07 |
| Thresholds | W13$4 | 1.44 | 0.11 |
| Thresholds | W14$1 | -1.73 | 0.13 |
| Thresholds | W14$2 | -0.78 | 0.08 |
| Thresholds | W14$3 | 0.06 | 0.07 |
| Thresholds | W14$4 | 1.32 | 0.10 |
| Thresholds | W15$1 | -1.13 | 0.09 |
| Thresholds | W15$2 | -0.23 | 0.07 |
| Thresholds | W15$3 | 0.40 | 0.07 |
| Thresholds | W15$4 | 1.47 | 0.11 |
| Thresholds | W16$1 | -0.89 | 0.08 |
| Thresholds | W16$2 | -0.11 | 0.07 |
| Thresholds | W16$3 | 0.67 | 0.08 |
| Thresholds | W16$4 | 1.69 | 0.12 |
| Thresholds | W17$1 | -0.86 | 0.08 |
| Thresholds | W17$2 | -0.06 | 0.07 |
| Thresholds | W17$3 | 0.53 | 0.08 |
| Thresholds | W17$4 | 1.76 | 0.13 |
| Thresholds | W18$1 | -1.30 | 0.10 |
| Thresholds | W18$2 | -0.55 | 0.08 |
| Thresholds | W18$3 | 0.37 | 0.07 |
| Thresholds | W18$4 | 1.60 | 0.12 |
| Thresholds | W19$1 | -1.06 | 0.09 |
| Thresholds | W19$2 | -0.23 | 0.07 |
| Thresholds | W19$3 | 0.40 | 0.07 |
| Thresholds | W19$4 | 1.57 | 0.11 |
| Thresholds | W20$1 | -0.84 | 0.08 |
| Thresholds | W20$2 | -0.20 | 0.07 |
| Thresholds | W20$3 | 0.60 | 0.08 |
| Thresholds | W20$4 | 1.63 | 0.12 |
| Thresholds | W21$1 | -1.13 | 0.09 |
| Thresholds | W21$2 | -0.54 | 0.08 |
| Thresholds | W21$3 | 0.33 | 0.07 |
| Thresholds | W21$4 | 1.66 | 0.12 |
| Thresholds | W23$1 | -1.57 | 0.11 |
| Thresholds | W23$2 | -0.55 | 0.08 |
| Thresholds | W23$3 | 0.32 | 0.07 |
| Thresholds | W23$4 | 1.49 | 0.11 |
| Thresholds | W24$1 | -0.71 | 0.08 |
| Thresholds | W24$2 | -0.13 | 0.07 |
| Thresholds | W24$3 | 0.50 | 0.08 |
| Thresholds | W24$4 | 1.73 | 0.13 |
| Thresholds | W27$1 | -0.64 | 0.08 |
| Thresholds | W27$2 | -0.04 | 0.07 |
| Thresholds | W27$3 | 0.59 | 0.08 |
| Thresholds | W27$4 | 1.69 | 0.12 |
| Thresholds | W28$1 | -1.36 | 0.10 |
| Thresholds | W28$2 | -0.45 | 0.07 |
| Thresholds | W28$3 | 0.29 | 0.07 |
| Thresholds | W28$4 | 1.26 | 0.10 |
| Thresholds | W29$1 | -1.54 | 0.11 |
| Thresholds | W29$2 | -0.57 | 0.08 |
| Thresholds | W29$3 | 0.27 | 0.07 |
| Thresholds | W29$4 | 1.23 | 0.10 |

*Note.* F1.BY is the loading on F1; F2.BY is the loading on F2; F3.BY is the loading on F3.

F2.WITH is the correlation of F2 with the other factor(s); F3.WITH is the correlation of F3 with the other factor(s).

SE=Standard Error.

**Table B4***The Three-factor Model for the CAQ-W with 30 Items*

| Parameter Type | Item/Factor | Estimate | SE |
| --- | --- | --- | --- |
| F1.BY | W17 | 0.82 | 0.02 |
| F1.BY | W1 | 0.77 | 0.02 |
| F1.BY | W20 | 0.76 | 0.03 |
| F1.BY | W27 | 0.80 | 0.02 |
| F1.BY | W16 | 0.73 | 0.03 |
| F1.BY | W15 | 0.84 | 0.02 |
| F1.BY | W24 | 0.81 | 0.02 |
| F1.BY | W19 | 0.84 | 0.02 |
| F1.BY | W2 | 0.69 | 0.03 |
| F1.BY | W10 | 0.83 | 0.02 |
| F1.BY | W9 | 0.78 | 0.02 |
| F1.BY | W12 | 0.83 | 0.02 |
| F1.BY | W23 | 0.79 | 0.02 |
| F1.BY | W26 | 0.58 | 0.04 |
| F2.BY | W29 | 0.83 | 0.02 |
| F2.BY | W28 | 0.74 | 0.03 |
| F2.BY | W8 | 0.84 | 0.02 |
| F2.BY | W13 | 0.87 | 0.02 |
| F2.BY | W21 | 0.84 | 0.02 |
| F2.BY | W18 | 0.83 | 0.02 |
| F2.BY | W7 | 0.76 | 0.03 |
| F2.BY | W5 | 0.84 | 0.02 |
| F2.BY | W4 | 0.80 | 0.02 |
| F3.BY | W6 | 0.67 | 0.03 |
| F3.BY | W3 | 0.73 | 0.03 |
| F3.BY | W14 | 0.73 | 0.03 |
| F3.BY | W11 | 0.81 | 0.03 |
| F3.BY | W22 | 0.84 | 0.02 |
| F3.BY | W25 | 0.74 | 0.03 |
| F3.BY | W30 | 0.84 | 0.02 |
| F2WITH | F1 | 0.32 | 0.05 |
| F3WITH | F1 | 0.87 | 0.02 |
| F3 WITH | F2 | 0.40 | 0.05 |
| Thresholds | W1$1 | -0.59 | 0.08 |
| Thresholds | W1$2 | 0.03 | 0.07 |
| Thresholds | W1$3 | 0.64 | 0.08 |
| Thresholds | W1$4 | 1.95 | 0.15 |
| Thresholds | W2$1 | -0.64 | 0.08 |
| Thresholds | W2$2 | 0.00 | 0.07 |
| Thresholds | W2$3 | 0.90 | 0.08 |
| Thresholds | W2$4 | 1.76 | 0.13 |
| Thresholds | W3$1 | -1.49 | 0.11 |
| Thresholds | W3$2 | -0.91 | 0.08 |
| Thresholds | W3$3 | -0.11 | 0.07 |
| Thresholds | W3$4 | 1.07 | 0.09 |
| Thresholds | W4$1 | -1.63 | 0.12 |
| Thresholds | W4$2 | -0.71 | 0.08 |
| Thresholds | W4$3 | -0.02 | 0.07 |
| Thresholds | W4$4 | 1.19 | 0.09 |
| Thresholds | W5$1 | -1.69 | 0.12 |
| Thresholds | W5$2 | -0.82 | 0.08 |
| Thresholds | W5$3 | -0.03 | 0.07 |
| Thresholds | W5$4 | 1.16 | 0.09 |
| Thresholds | W6$1 | -1.95 | 0.15 |
| Thresholds | W6$2 | -1.03 | 0.09 |
| Thresholds | W6$3 | -0.25 | 0.07 |
| Thresholds | W6$4 | 0.91 | 0.08 |
| Thresholds | W7$1 | -1.80 | 0.14 |
| Thresholds | W7$2 | -0.99 | 0.09 |
| Thresholds | W7$3 | -0.15 | 0.07 |
| Thresholds | W7$4 | 1.28 | 0.10 |
| Thresholds | W8$1 | -1.40 | 0.10 |
| Thresholds | W8$2 | -0.53 | 0.08 |
| Thresholds | W8$3 | 0.15 | 0.07 |
| Thresholds | W8$4 | 1.26 | 0.10 |
| Thresholds | W9$1 | -0.96 | 0.09 |
| Thresholds | W9$2 | -0.27 | 0.07 |
| Thresholds | W9$3 | 0.37 | 0.07 |
| Thresholds | W9$4 | 1.24 | 0.10 |
| Thresholds | W10$1 | -0.85 | 0.08 |
| Thresholds | W10$2 | -0.10 | 0.07 |
| Thresholds | W10$3 | 0.52 | 0.08 |
| Thresholds | W10$4 | 1.47 | 0.11 |
| Thresholds | W11$1 | -1.57 | 0.11 |
| Thresholds | W11$2 | -0.70 | 0.08 |
| Thresholds | W11$3 | 0.09 | 0.07 |
| Thresholds | W11$4 | 1.23 | 0.10 |
| Thresholds | W12$1 | -1.57 | 0.11 |
| Thresholds | W12$2 | -0.42 | 0.07 |
| Thresholds | W12$3 | 0.35 | 0.07 |
| Thresholds | W12$4 | 1.36 | 0.10 |
| Thresholds | W13$1 | -1.42 | 0.11 |
| Thresholds | W13$2 | -0.50 | 0.08 |
| Thresholds | W13$3 | 0.35 | 0.07 |
| Thresholds | W13$4 | 1.44 | 0.11 |
| Thresholds | W14$1 | -1.73 | 0.13 |
| Thresholds | W14$2 | -0.78 | 0.08 |
| Thresholds | W14$3 | 0.06 | 0.07 |
| Thresholds | W14$4 | 1.32 | 0.10 |
| Thresholds | W15$1 | -1.13 | 0.09 |
| Thresholds | W15$2 | -0.23 | 0.07 |
| Thresholds | W15$3 | 0.40 | 0.07 |
| Thresholds | W15$4 | 1.47 | 0.11 |
| Thresholds | W16$1 | -0.89 | 0.08 |
| Thresholds | W16$2 | -0.11 | 0.07 |
| Thresholds | W16$3 | 0.67 | 0.08 |
| Thresholds | W16$4 | 1.69 | 0.12 |
| Thresholds | W17$1 | -0.86 | 0.08 |
| Thresholds | W17$2 | -0.06 | 0.07 |
| Thresholds | W17$3 | 0.53 | 0.08 |
| Thresholds | W17$4 | 1.76 | 0.13 |
| Thresholds | W18$1 | -1.30 | 0.10 |
| Thresholds | W18$2 | -0.55 | 0.08 |
| Thresholds | W18$3 | 0.37 | 0.07 |
| Thresholds | W18$4 | 1.60 | 0.12 |
| Thresholds | W19$1 | -1.06 | 0.09 |
| Thresholds | W19$2 | -0.23 | 0.07 |
| Thresholds | W19$3 | 0.40 | 0.07 |
| Thresholds | W19$4 | 1.57 | 0.11 |
| Thresholds | W20$1 | -0.84 | 0.08 |
| Thresholds | W20$2 | -0.20 | 0.07 |
| Thresholds | W20$3 | 0.60 | 0.08 |
| Thresholds | W20$4 | 1.63 | 0.12 |
| Thresholds | W21$1 | -1.13 | 0.09 |
| Thresholds | W21$2 | -0.54 | 0.08 |
| Thresholds | W21$3 | 0.33 | 0.07 |
| Thresholds | W21$4 | 1.66 | 0.12 |
| Thresholds | W22$1 | -1.24 | 0.10 |
| Thresholds | W22$2 | -0.47 | 0.07 |
| Thresholds | W22$3 | 0.32 | 0.07 |
| Thresholds | W22$4 | 1.36 | 0.10 |
| Thresholds | W23$1 | -1.57 | 0.11 |
| Thresholds | W23$2 | -0.55 | 0.08 |
| Thresholds | W23$3 | 0.32 | 0.07 |
| Thresholds | W23$4 | 1.49 | 0.11 |
| Thresholds | W24$1 | -0.71 | 0.08 |
| Thresholds | W24$2 | -0.13 | 0.07 |
| Thresholds | W24$3 | 0.50 | 0.08 |
| Thresholds | W24$4 | 1.73 | 0.13 |
| Thresholds | W25$1 | -1.36 | 0.10 |
| Thresholds | W25$2 | -0.49 | 0.08 |
| Thresholds | W25$3 | 0.35 | 0.07 |
| Thresholds | W25$4 | 1.34 | 0.10 |
| Thresholds | W26$1 | -0.94 | 0.08 |
| Thresholds | W26$2 | -0.21 | 0.07 |
| Thresholds | W26$3 | 0.54 | 0.08 |
| Thresholds | W26$4 | 1.76 | 0.13 |
| Thresholds | W27$1 | -0.64 | 0.08 |
| Thresholds | W27$2 | -0.04 | 0.07 |
| Thresholds | W27$3 | 0.59 | 0.08 |
| Thresholds | W27$4 | 1.69 | 0.12 |
| Thresholds | W28$1 | -1.36 | 0.10 |
| Thresholds | W28$2 | -0.45 | 0.07 |
| Thresholds | W28$3 | 0.29 | 0.07 |
| Thresholds | W28$4 | 1.26 | 0.10 |
| Thresholds | W29$1 | -1.54 | 0.11 |
| Thresholds | W29$2 | -0.57 | 0.08 |
| Thresholds | W29$3 | 0.27 | 0.07 |
| Thresholds | W29$4 | 1.23 | 0.10 |
| Thresholds | W30$1 | -0.95 | 0.08 |
| Thresholds | W30$2 | -0.33 | 0.07 |
| Thresholds | W30$3 | 0.49 | 0.07 |
| Thresholds | W30$4 | 1.40 | 0.10 |

*Note.* F1.BY is the loading on F1; F2.BY is the loading on F2; F3.BY is the loading on F3.

F2.WITH is the correlation of F2 with the other factor(s); F3.WITH is the correlation of F3 with the other factor(s).

SE=Standard Error.

**Table B5***The One-factor Model for the CAQ-GE with 24 Items*

| Parameter Type | Item/Factor | Estimate | SE |
| --- | --- | --- | --- |
| F1.BY | GE1 | 0.71 | 0.03 |
| F1.BY | GE2 | 0.72 | 0.03 |
| F1.BY | GE4 | 0.77 | 0.02 |
| F1.BY | GE5 | 0.86 | 0.02 |
| F1.BY | GE7 | 0.76 | 0.03 |
| F1.BY | GE8 | 0.85 | 0.02 |
| F1.BY | GE9 | 0.77 | 0.02 |
| F1.BY | GE10 | 0.74 | 0.03 |
| F1.BY | GE12 | 0.86 | 0.02 |
| F1.BY | GE16 | 0.74 | 0.02 |
| F1.BY | GE17 | 0.77 | 0.03 |
| F1.BY | GE18 | 0.84 | 0.02 |
| F1.BY | GE20 | 0.85 | 0.02 |
| F1.BY | GE21 | 0.84 | 0.02 |
| F1.BY | GE22 | 0.79 | 0.02 |
| F1.BY | GE24 | 0.82 | 0.02 |
| F1.BY | GE25 | 0.84 | 0.02 |
| F1.BY | GE3 | 0.70 | 0.03 |
| F1.BY | GE6 | 0.69 | 0.03 |
| F1.BY | GE11 | 0.68 | 0.03 |
| F1.BY | GE14 | 0.45 | 0.04 |
| F1.BY | GE15 | 0.74 | 0.03 |
| F1.BY | GE19 | 0.68 | 0.03 |
| F1.BY | GE23 | 0.66 | 0.03 |
| Thresholds | GE1$1 | -1.47 | 0.11 |
| Thresholds | GE1$2 | -0.43 | 0.07 |
| Thresholds | GE1$3 | 0.32 | 0.07 |
| Thresholds | GE1$4 | 1.63 | 0.12 |
| Thresholds | GE2$1 | -1.00 | 0.09 |
| Thresholds | GE2$2 | -0.31 | 0.07 |
| Thresholds | GE2$3 | 0.51 | 0.08 |
| Thresholds | GE2$4 | 1.34 | 0.10 |
| Thresholds | GE3$1 | -0.74 | 0.08 |
| Thresholds | GE3$2 | 0.15 | 0.07 |
| Thresholds | GE3$3 | 0.85 | 0.08 |
| Thresholds | GE3$4 | 1.89 | 0.14 |
| Thresholds | GE4$1 | -0.86 | 0.08 |
| Thresholds | GE4$2 | -0.12 | 0.07 |
| Thresholds | GE4$3 | 0.56 | 0.08 |
| Thresholds | GE4$4 | 1.57 | 0.11 |
| Thresholds | GE5$1 | -0.67 | 0.08 |
| Thresholds | GE5$2 | 0.06 | 0.07 |
| Thresholds | GE5$3 | 0.63 | 0.08 |
| Thresholds | GE5$4 | 1.63 | 0.12 |
| Thresholds | GE6$1 | -0.92 | 0.08 |
| Thresholds | GE6$2 | -0.30 | 0.07 |
| Thresholds | GE6$3 | 0.49 | 0.08 |
| Thresholds | GE6$4 | 1.38 | 0.10 |
| Thresholds | GE7$1 | -0.66 | 0.08 |
| Thresholds | GE7$2 | -0.04 | 0.07 |
| Thresholds | GE7$3 | 0.54 | 0.08 |
| Thresholds | GE7$4 | 1.47 | 0.11 |
| Thresholds | GE8$1 | -0.79 | 0.08 |
| Thresholds | GE8$2 | -0.08 | 0.07 |
| Thresholds | GE8$3 | 0.69 | 0.08 |
| Thresholds | GE8$4 | 1.63 | 0.12 |
| Thresholds | GE9$1 | -0.50 | 0.08 |
| Thresholds | GE9$2 | 0.22 | 0.07 |
| Thresholds | GE9$3 | 0.84 | 0.08 |
| Thresholds | GE9$4 | 1.66 | 0.12 |
| Thresholds | GE10$1 | -0.62 | 0.08 |
| Thresholds | GE10$2 | 0.05 | 0.07 |
| Thresholds | GE10$3 | 0.67 | 0.08 |
| Thresholds | GE10$4 | 1.63 | 0.12 |
| Thresholds | GE11$1 | -0.90 | 0.08 |
| Thresholds | GE11$2 | -0.13 | 0.07 |
| Thresholds | GE11$3 | 0.57 | 0.08 |
| Thresholds | GE11$4 | 1.60 | 0.12 |
| Thresholds | GE12$1 | -0.59 | 0.08 |
| Thresholds | GE12$2 | 0.14 | 0.07 |
| Thresholds | GE12$3 | 0.58 | 0.08 |
| Thresholds | GE12$4 | 1.54 | 0.11 |
| Thresholds | GE14$1 | -1.49 | 0.11 |
| Thresholds | GE14$2 | -0.72 | 0.08 |
| Thresholds | GE14$3 | 0.12 | 0.07 |
| Thresholds | GE14$4 | 1.24 | 0.10 |
| Thresholds | GE15$1 | -0.70 | 0.08 |
| Thresholds | GE15$2 | 0.14 | 0.07 |
| Thresholds | GE15$3 | 0.89 | 0.08 |
| Thresholds | GE15$4 | 1.66 | 0.12 |
| Thresholds | GE16$1 | -0.60 | 0.08 |
| Thresholds | GE16$2 | 0.15 | 0.07 |
| Thresholds | GE16$3 | 0.69 | 0.08 |
| Thresholds | GE16$4 | 1.89 | 0.14 |
| Thresholds | GE17$1 | -0.15 | 0.07 |
| Thresholds | GE17$2 | 0.31 | 0.07 |
| Thresholds | GE17$3 | 0.94 | 0.08 |
| Thresholds | GE17$4 | 1.63 | 0.12 |
| Thresholds | GE18$1 | -0.34 | 0.07 |
| Thresholds | GE18$2 | 0.24 | 0.07 |
| Thresholds | GE18$3 | 0.84 | 0.08 |
| Thresholds | GE18$4 | 1.69 | 0.12 |
| Thresholds | GE19$1 | -1.04 | 0.09 |
| Thresholds | GE19$2 | -0.08 | 0.07 |
| Thresholds | GE19$3 | 0.70 | 0.08 |
| Thresholds | GE19$4 | 1.63 | 0.12 |
| Thresholds | GE20$1 | -0.76 | 0.08 |
| Thresholds | GE20$2 | 0.09 | 0.07 |
| Thresholds | GE20$3 | 0.71 | 0.08 |
| Thresholds | GE20$4 | 1.76 | 0.13 |
| Thresholds | GE21$1 | -0.29 | 0.07 |
| Thresholds | GE21$2 | 0.33 | 0.07 |
| Thresholds | GE21$3 | 1.04 | 0.09 |
| Thresholds | GE21$4 | 1.80 | 0.14 |
| Thresholds | GE22$1 | -0.22 | 0.07 |
| Thresholds | GE22$2 | 0.38 | 0.07 |
| Thresholds | GE22$3 | 1.01 | 0.09 |
| Thresholds | GE22$4 | 1.89 | 0.14 |
| Thresholds | GE23$1 | -0.92 | 0.08 |
| Thresholds | GE23$2 | -0.08 | 0.07 |
| Thresholds | GE23$3 | 0.67 | 0.08 |
| Thresholds | GE23$4 | 1.66 | 0.12 |
| Thresholds | GE24$1 | -0.65 | 0.08 |
| Thresholds | GE24$2 | 0.24 | 0.07 |
| Thresholds | GE24$3 | 0.79 | 0.08 |
| Thresholds | GE24$4 | 1.66 | 0.12 |
| Thresholds | GE25$1 | -0.61 | 0.08 |
| Thresholds | GE25$2 | 0.09 | 0.07 |
| Thresholds | GE25$3 | 0.73 | 0.08 |
| Thresholds | GE25$4 | 1.52 | 0.11 |

*Note.* F1.BY is the loading on F1.

SE=Standard Error.

**Table B6***The Two-factor Model for the CAQ-GE with 24 Items*

| Parameter Type | Item/Factor | Estimate | SE |
| --- | --- | --- | --- |
| F1.BY | GE1 | 0.72 | 0.03 |
| F1.BY | GE2 | 0.74 | 0.03 |
| F1.BY | GE4 | 0.78 | 0.02 |
| F1.BY | GE5 | 0.87 | 0.02 |
| F1.BY | GE7 | 0.77 | 0.03 |
| F1.BY | GE8 | 0.86 | 0.02 |
| F1.BY | GE9 | 0.78 | 0.02 |
| F1.BY | GE10 | 0.75 | 0.03 |
| F1.BY | GE12 | 0.87 | 0.02 |
| F1.BY | GE16 | 0.75 | 0.02 |
| F1.BY | GE17 | 0.78 | 0.02 |
| F1.BY | GE18 | 0.85 | 0.02 |
| F1.BY | GE20 | 0.86 | 0.02 |
| F1.BY | GE21 | 0.85 | 0.02 |
| F1.BY | GE22 | 0.80 | 0.02 |
| F1.BY | GE24 | 0.83 | 0.02 |
| F1.BY | GE25 | 0.84 | 0.02 |
| F2.BY | GE3 | 0.82 | 0.03 |
| F2.BY | GE6 | 0.81 | 0.03 |
| F2.BY | GE11 | 0.80 | 0.03 |
| F2.BY | GE14 | 0.54 | 0.04 |
| F2.BY | GE15 | 0.87 | 0.02 |
| F2.BY | GE19 | 0.79 | 0.03 |
| F2.BY | GE23 | 0.77 | 0.03 |
| F2.WITH | F1 | 0.71 | 0.03 |
| Thresholds | GE1$1 | -1.47 | 0.11 |
| Thresholds | GE1$2 | -0.43 | 0.07 |
| Thresholds | GE1$3 | 0.32 | 0.07 |
| Thresholds | GE1$4 | 1.63 | 0.12 |
| Thresholds | GE2$1 | -1.00 | 0.09 |
| Thresholds | GE2$2 | -0.31 | 0.07 |
| Thresholds | GE2$3 | 0.51 | 0.08 |
| Thresholds | GE2$4 | 1.34 | 0.10 |
| Thresholds | GE3$1 | -0.74 | 0.08 |
| Thresholds | GE3$2 | 0.15 | 0.07 |
| Thresholds | GE3$3 | 0.85 | 0.08 |
| Thresholds | GE3$4 | 1.89 | 0.14 |
| Thresholds | GE4$1 | -0.86 | 0.08 |
| Thresholds | GE4$2 | -0.12 | 0.07 |
| Thresholds | GE4$3 | 0.56 | 0.08 |
| Thresholds | GE4$4 | 1.57 | 0.11 |
| Thresholds | GE5$1 | -0.67 | 0.08 |
| Thresholds | GE5$2 | 0.06 | 0.07 |
| Thresholds | GE5$3 | 0.63 | 0.08 |
| Thresholds | GE5$4 | 1.63 | 0.12 |
| Thresholds | GE6$1 | -0.92 | 0.08 |
| Thresholds | GE6$2 | -0.30 | 0.07 |
| Thresholds | GE6$3 | 0.49 | 0.08 |
| Thresholds | GE6$4 | 1.38 | 0.10 |
| Thresholds | GE7$1 | -0.66 | 0.08 |
| Thresholds | GE7$2 | -0.04 | 0.07 |
| Thresholds | GE7$3 | 0.54 | 0.08 |
| Thresholds | GE7$4 | 1.47 | 0.11 |
| Thresholds | GE8$1 | -0.79 | 0.08 |
| Thresholds | GE8$2 | -0.08 | 0.07 |
| Thresholds | GE8$3 | 0.69 | 0.08 |
| Thresholds | GE8$4 | 1.63 | 0.12 |
| Thresholds | GE9$1 | -0.50 | 0.08 |
| Thresholds | GE9$2 | 0.22 | 0.07 |
| Thresholds | GE9$3 | 0.84 | 0.08 |
| Thresholds | GE9$4 | 1.66 | 0.12 |
| Thresholds | GE10$1 | -0.62 | 0.08 |
| Thresholds | GE10$2 | 0.05 | 0.07 |
| Thresholds | GE10$3 | 0.67 | 0.08 |
| Thresholds | GE10$4 | 1.63 | 0.12 |
| Thresholds | GE11$1 | -0.90 | 0.08 |
| Thresholds | GE11$2 | -0.13 | 0.07 |
| Thresholds | GE11$3 | 0.57 | 0.08 |
| Thresholds | GE11$4 | 1.60 | 0.12 |
| Thresholds | GE12$1 | -0.59 | 0.08 |
| Thresholds | GE12$2 | 0.14 | 0.07 |
| Thresholds | GE12$3 | 0.58 | 0.08 |
| Thresholds | GE12$4 | 1.54 | 0.11 |
| Thresholds | GE14$1 | -1.49 | 0.11 |
| Thresholds | GE14$2 | -0.72 | 0.08 |
| Thresholds | GE14$3 | 0.12 | 0.07 |
| Thresholds | GE14$4 | 1.24 | 0.10 |
| Thresholds | GE15$1 | -0.70 | 0.08 |
| Thresholds | GE15$2 | 0.14 | 0.07 |
| Thresholds | GE15$3 | 0.89 | 0.08 |
| Thresholds | GE15$4 | 1.66 | 0.12 |
| Thresholds | GE16$1 | -0.60 | 0.08 |
| Thresholds | GE16$2 | 0.15 | 0.07 |
| Thresholds | GE16$3 | 0.69 | 0.08 |
| Thresholds | GE16$4 | 1.89 | 0.14 |
| Thresholds | GE17$1 | -0.15 | 0.07 |
| Thresholds | GE17$2 | 0.31 | 0.07 |
| Thresholds | GE17$3 | 0.94 | 0.08 |
| Thresholds | GE17$4 | 1.63 | 0.12 |
| Thresholds | GE18$1 | -0.34 | 0.07 |
| Thresholds | GE18$2 | 0.24 | 0.07 |
| Thresholds | GE18$3 | 0.84 | 0.08 |
| Thresholds | GE18$4 | 1.69 | 0.12 |
| Thresholds | GE19$1 | -1.04 | 0.09 |
| Thresholds | GE19$2 | -0.08 | 0.07 |
| Thresholds | GE19$3 | 0.70 | 0.08 |
| Thresholds | GE19$4 | 1.63 | 0.12 |
| Thresholds | GE20$1 | -0.76 | 0.08 |
| Thresholds | GE20$2 | 0.09 | 0.07 |
| Thresholds | GE20$3 | 0.71 | 0.08 |
| Thresholds | GE20$4 | 1.76 | 0.13 |
| Thresholds | GE21$1 | -0.29 | 0.07 |
| Thresholds | GE21$2 | 0.33 | 0.07 |
| Thresholds | GE21$3 | 1.04 | 0.09 |
| Thresholds | GE21$4 | 1.80 | 0.14 |
| Thresholds | GE22$1 | -0.22 | 0.07 |
| Thresholds | GE22$2 | 0.38 | 0.07 |
| Thresholds | GE22$3 | 1.01 | 0.09 |
| Thresholds | GE22$4 | 1.89 | 0.14 |
| Thresholds | GE23$1 | -0.92 | 0.08 |
| Thresholds | GE23$2 | -0.08 | 0.07 |
| Thresholds | GE23$3 | 0.67 | 0.08 |
| Thresholds | GE23$4 | 1.66 | 0.12 |
| Thresholds | GE24$1 | -0.65 | 0.08 |
| Thresholds | GE24$2 | 0.24 | 0.07 |
| Thresholds | GE24$3 | 0.79 | 0.08 |
| Thresholds | GE24$4 | 1.66 | 0.12 |
| Thresholds | GE25$1 | -0.61 | 0.08 |
| Thresholds | GE25$2 | 0.09 | 0.07 |
| Thresholds | GE25$3 | 0.73 | 0.08 |
| Thresholds | GE25$4 | 1.52 | 0.11 |

*Note.* F1.BY is the loading on F1; F2.BY is the loading on F2.

F2.WITH is the correlation of F2 with the other factor(s).

SE=Standard Error.

**Table B7***The Two-factor Model for the CAQ-GE with 25 Items*

| Parameter Type | Item/Factor | Estimate | SE |
| --- | --- | --- | --- |
| F1.BY | GE1 | 0.72 | 0.03 |
| F1.BY | GE2 | 0.74 | 0.03 |
| F1.BY | GE4 | 0.78 | 0.02 |
| F1.BY | GE5 | 0.87 | 0.02 |
| F1.BY | GE7 | 0.77 | 0.03 |
| F1.BY | GE8 | 0.86 | 0.02 |
| F1.BY | GE9 | 0.78 | 0.02 |
| F1.BY | GE10 | 0.76 | 0.03 |
| F1.BY | GE12 | 0.87 | 0.02 |
| F1.BY | GE16 | 0.75 | 0.03 |
| F1.BY | GE17 | 0.79 | 0.02 |
| F1.BY | GE18 | 0.84 | 0.02 |
| F1.BY | GE20 | 0.86 | 0.02 |
| F1.BY | GE21 | 0.85 | 0.02 |
| F1.BY | GE22 | 0.80 | 0.02 |
| F1.BY | GE24 | 0.83 | 0.02 |
| F1.BY | GE25 | 0.85 | 0.02 |
| F1.BY | GE13 | 0.64 | 0.03 |
| F2.BY | GE3 | 0.82 | 0.03 |
| F2.BY | GE6 | 0.81 | 0.03 |
| F2.BY | GE11 | 0.80 | 0.03 |
| F2.BY | GE14 | 0.54 | 0.04 |
| F2.BY | GE15 | 0.87 | 0.02 |
| F2.BY | GE19 | 0.79 | 0.03 |
| F2.BY | GE23 | 0.77 | 0.03 |
| F2.WITH | F1 | 0.71 | 0.03 |
| Thresholds | GE1$1 | -1.47 | 0.11 |
| Thresholds | GE1$2 | -0.43 | 0.07 |
| Thresholds | GE1$3 | 0.32 | 0.07 |
| Thresholds | GE1$4 | 1.63 | 0.12 |
| Thresholds | GE2$1 | -1.00 | 0.09 |
| Thresholds | GE2$2 | -0.31 | 0.07 |
| Thresholds | GE2$3 | 0.51 | 0.08 |
| Thresholds | GE2$4 | 1.34 | 0.10 |
| Thresholds | GE3$1 | -0.74 | 0.08 |
| Thresholds | GE3$2 | 0.15 | 0.07 |
| Thresholds | GE3$3 | 0.85 | 0.08 |
| Thresholds | GE3$4 | 1.89 | 0.14 |
| Thresholds | GE4$1 | -0.86 | 0.08 |
| Thresholds | GE4$2 | -0.12 | 0.07 |
| Thresholds | GE4$3 | 0.56 | 0.08 |
| Thresholds | GE4$4 | 1.57 | 0.11 |
| Thresholds | GE5$1 | -0.67 | 0.08 |
| Thresholds | GE5$2 | 0.06 | 0.07 |
| Thresholds | GE5$3 | 0.63 | 0.08 |
| Thresholds | GE5$4 | 1.63 | 0.12 |
| Thresholds | GE6$1 | -0.92 | 0.08 |
| Thresholds | GE6$2 | -0.30 | 0.07 |
| Thresholds | GE6$3 | 0.49 | 0.08 |
| Thresholds | GE6$4 | 1.38 | 0.10 |
| Thresholds | GE7$1 | -0.66 | 0.08 |
| Thresholds | GE7$2 | -0.04 | 0.07 |
| Thresholds | GE7$3 | 0.54 | 0.08 |
| Thresholds | GE7$4 | 1.47 | 0.11 |
| Thresholds | GE8$1 | -0.79 | 0.08 |
| Thresholds | GE8$2 | -0.08 | 0.07 |
| Thresholds | GE8$3 | 0.69 | 0.08 |
| Thresholds | GE8$4 | 1.63 | 0.12 |
| Thresholds | GE9$1 | -0.50 | 0.08 |
| Thresholds | GE9$2 | 0.22 | 0.07 |
| Thresholds | GE9$3 | 0.84 | 0.08 |
| Thresholds | GE9$4 | 1.66 | 0.12 |
| Thresholds | GE10$1 | -0.62 | 0.08 |
| Thresholds | GE10$2 | 0.05 | 0.07 |
| Thresholds | GE10$3 | 0.67 | 0.08 |
| Thresholds | GE10$4 | 1.63 | 0.12 |
| Thresholds | GE11$1 | -0.90 | 0.08 |
| Thresholds | GE11$2 | -0.13 | 0.07 |
| Thresholds | GE11$3 | 0.57 | 0.08 |
| Thresholds | GE11$4 | 1.60 | 0.12 |
| Thresholds | GE12$1 | -0.59 | 0.08 |
| Thresholds | GE12$2 | 0.14 | 0.07 |
| Thresholds | GE12$3 | 0.58 | 0.08 |
| Thresholds | GE12$4 | 1.54 | 0.11 |
| Thresholds | GE13$1 | -0.47 | 0.07 |
| Thresholds | GE13$2 | 0.16 | 0.07 |
| Thresholds | GE13$3 | 0.81 | 0.08 |
| Thresholds | GE13$4 | 1.85 | 0.14 |
| Thresholds | GE14$1 | -1.49 | 0.11 |
| Thresholds | GE14$2 | -0.72 | 0.08 |
| Thresholds | GE14$3 | 0.12 | 0.07 |
| Thresholds | GE14$4 | 1.24 | 0.10 |
| Thresholds | GE15$1 | -0.70 | 0.08 |
| Thresholds | GE15$2 | 0.14 | 0.07 |
| Thresholds | GE15$3 | 0.89 | 0.08 |
| Thresholds | GE15$4 | 1.66 | 0.12 |
| Thresholds | GE16$1 | -0.60 | 0.08 |
| Thresholds | GE16$2 | 0.15 | 0.07 |
| Thresholds | GE16$3 | 0.69 | 0.08 |
| Thresholds | GE16$4 | 1.89 | 0.14 |
| Thresholds | GE17$1 | -0.15 | 0.07 |
| Thresholds | GE17$2 | 0.31 | 0.07 |
| Thresholds | GE17$3 | 0.94 | 0.08 |
| Thresholds | GE17$4 | 1.63 | 0.12 |
| Thresholds | GE18$1 | -0.34 | 0.07 |
| Thresholds | GE18$2 | 0.24 | 0.07 |
| Thresholds | GE18$3 | 0.84 | 0.08 |
| Thresholds | GE18$4 | 1.69 | 0.12 |
| Thresholds | GE19$1 | -1.04 | 0.09 |
| Thresholds | GE19$2 | -0.08 | 0.07 |
| Thresholds | GE19$3 | 0.70 | 0.08 |
| Thresholds | GE19$4 | 1.63 | 0.12 |
| Thresholds | GE20$1 | -0.76 | 0.08 |
| Thresholds | GE20$2 | 0.09 | 0.07 |
| Thresholds | GE20$3 | 0.71 | 0.08 |
| Thresholds | GE20$4 | 1.76 | 0.13 |
| Thresholds | GE21$1 | -0.29 | 0.07 |
| Thresholds | GE21$2 | 0.33 | 0.07 |
| Thresholds | GE21$3 | 1.04 | 0.09 |
| Thresholds | GE21$4 | 1.80 | 0.14 |
| Thresholds | GE22$1 | -0.22 | 0.07 |
| Thresholds | GE22$2 | 0.38 | 0.07 |
| Thresholds | GE22$3 | 1.01 | 0.09 |
| Thresholds | GE22$4 | 1.89 | 0.14 |
| Thresholds | GE23$1 | -0.92 | 0.08 |
| Thresholds | GE23$2 | -0.08 | 0.07 |
| Thresholds | GE23$3 | 0.67 | 0.08 |
| Thresholds | GE23$4 | 1.66 | 0.12 |
| Thresholds | GE24$1 | -0.65 | 0.08 |
| Thresholds | GE24$2 | 0.24 | 0.07 |
| Thresholds | GE24$3 | 0.79 | 0.08 |
| Thresholds | GE24$4 | 1.66 | 0.12 |
| Thresholds | GE25$1 | -0.61 | 0.08 |
| Thresholds | GE25$2 | 0.09 | 0.07 |
| Thresholds | GE25$3 | 0.73 | 0.08 |
| Thresholds | GE25$4 | 1.52 | 0.11 |

*Note.* F1.BY is the loading on F1; F2.BY is the loading on F2.

F2.WITH is the correlation of F2 with the other factor(s).

SE=Standard Error.

**Supplementary Material C: Sensitivity Analysis of Confirmatory Factor Analysis**

**Table C1***Comparison of Measure Model*

| Measure | Model | S-Bχ^2^ | *df* | SRMR | RMSEA | CFI | TLI |
| --- | --- | --- | --- | --- | --- | --- | --- |
| CAQ-W | 1-factor | 2038.27 | 275 | 0.17 | 0.14 (0.14, 0.15) | 0.59 | 0.55 |
|  | 2-factor | 755.27 | 274 | 0.08 | 0.07 (0.07, 0.08) | 0.89 | 0.88 |
|  | **3-factor** | 616.83 | 272 | 0.07 | 0.06 (0.06, 0.07) | 0.92 | 0.91 |
|  | 3-factor (without deleting items) | 951.04 | 402 | 0.07 | 0.07 (0.06, 0.07) | 0.9 | 0.89 |
|  |  |  |  |  |  |  |  |
| CAQ-GE | 1-factor | 935.27 | 252 | 0.08 | 0.09 (0.09, 0.1) | 0.82 | 0.81 |
|  | **2-factor** | 560.18 | 251 | 0.05 | 0.06 (0.06, 0.07) | 0.92 | 0.91 |
|  | 2-factor (without deleting items) | 591.45 | 274 | 0.05 | 0.06 (0.05, 0.07) | 0.92 | 0.91 |

**Table C2***The One-factor Model for the CAQ-W with 25 Items*

| Parameter Type | Item/Factor | Estimate | SE |
| --- | --- | --- | --- |
| F1.BY | W9 | 0.73 | 0.03 |
| F1.BY | W10 | 0.79 | 0.02 |
| F1.BY | W17 | 0.79 | 0.02 |
| F1.BY | W15 | 0.79 | 0.03 |
| F1.BY | W1 | 0.73 | 0.03 |
| F1.BY | W2 | 0.62 | 0.05 |
| F1.BY | W12 | 0.77 | 0.03 |
| F1.BY | W16 | 0.67 | 0.04 |
| F1.BY | W19 | 0.80 | 0.03 |
| F1.BY | W20 | 0.71 | 0.04 |
| F1.BY | W23 | 0.74 | 0.03 |
| F1.BY | W24 | 0.77 | 0.03 |
| F1.BY | W27 | 0.75 | 0.03 |
| F1.BY | W6 | 0.49 | 0.05 |
| F1.BY | W3 | 0.57 | 0.04 |
| F1.BY | W14 | 0.58 | 0.04 |
| F1.BY | W5 | 0.32 | 0.08 |
| F1.BY | W8 | 0.33 | 0.08 |
| F1.BY | W13 | 0.52 | 0.06 |
| F1.BY | W29 | 0.28 | 0.08 |
| F1.BY | W18 | 0.45 | 0.07 |
| F1.BY | W4 | 0.35 | 0.07 |
| F1.BY | W7 | 0.33 | 0.07 |
| F1.BY | W21 | 0.45 | 0.07 |
| F1.BY | W28 | 0.23 | 0.07 |
| Intercepts | W1 | 2.09 | 0.07 |
| Intercepts | W2 | 2.16 | 0.08 |
| Intercepts | W3 | 3.18 | 0.15 |
| Intercepts | W4 | 3.12 | 0.13 |
| Intercepts | W5 | 3.26 | 0.14 |
| Intercepts | W6 | 3.58 | 0.16 |
| Intercepts | W7 | 3.62 | 0.18 |
| Intercepts | W8 | 2.82 | 0.11 |
| Intercepts | W9 | 2.32 | 0.09 |
| Intercepts | W10 | 2.23 | 0.08 |
| Intercepts | W12 | 2.87 | 0.11 |
| Intercepts | W13 | 2.88 | 0.12 |
| Intercepts | W14 | 3.30 | 0.14 |
| Intercepts | W15 | 2.49 | 0.09 |
| Intercepts | W16 | 2.34 | 0.09 |
| Intercepts | W17 | 2.29 | 0.08 |
| Intercepts | W18 | 2.88 | 0.12 |
| Intercepts | W19 | 2.46 | 0.09 |
| Intercepts | W20 | 2.32 | 0.09 |
| Intercepts | W21 | 2.74 | 0.12 |
| Intercepts | W23 | 3.04 | 0.12 |
| Intercepts | W24 | 2.18 | 0.08 |
| Intercepts | W27 | 2.10 | 0.07 |
| Intercepts | W28 | 2.72 | 0.11 |
| Intercepts | W29 | 2.92 | 0.11 |
| Variances | F1 | 1.00 | 0.00 |
| Residual.Variances | W1 | 0.47 | 0.05 |
| Residual.Variances | W2 | 0.61 | 0.06 |
| Residual.Variances | W3 | 0.67 | 0.05 |
| Residual.Variances | W4 | 0.88 | 0.05 |
| Residual.Variances | W5 | 0.90 | 0.05 |
| Residual.Variances | W6 | 0.76 | 0.05 |
| Residual.Variances | W7 | 0.89 | 0.05 |
| Residual.Variances | W8 | 0.89 | 0.05 |
| Residual.Variances | W9 | 0.47 | 0.04 |
| Residual.Variances | W10 | 0.38 | 0.04 |
| Residual.Variances | W12 | 0.41 | 0.04 |
| Residual.Variances | W13 | 0.74 | 0.06 |
| Residual.Variances | W14 | 0.67 | 0.05 |
| Residual.Variances | W15 | 0.37 | 0.04 |
| Residual.Variances | W16 | 0.55 | 0.05 |
| Residual.Variances | W17 | 0.37 | 0.04 |
| Residual.Variances | W18 | 0.80 | 0.06 |
| Residual.Variances | W19 | 0.37 | 0.04 |
| Residual.Variances | W20 | 0.50 | 0.06 |
| Residual.Variances | W21 | 0.80 | 0.06 |
| Residual.Variances | W23 | 0.46 | 0.04 |
| Residual.Variances | W24 | 0.41 | 0.04 |
| Residual.Variances | W27 | 0.44 | 0.04 |
| Residual.Variances | W28 | 0.95 | 0.03 |
| Residual.Variances | W29 | 0.92 | 0.04 |

*Note.* F1.BY is the loading on F1.

SE=Standard Error.

**Table C3***The Two-factor Model for the CAQ-W with 25 Items*

| Parameter Type | Item/Factor | Estimate | SE |
| --- | --- | --- | --- |
| F1.BY | W9 | 0.73 | 0.03 |
| F1.BY | W10 | 0.80 | 0.02 |
| F1.BY | W17 | 0.79 | 0.02 |
| F1.BY | W15 | 0.81 | 0.02 |
| F1.BY | W1 | 0.74 | 0.03 |
| F1.BY | W2 | 0.66 | 0.04 |
| F1.BY | W12 | 0.76 | 0.03 |
| F1.BY | W16 | 0.70 | 0.03 |
| F1.BY | W19 | 0.81 | 0.03 |
| F1.BY | W20 | 0.73 | 0.03 |
| F1.BY | W23 | 0.74 | 0.03 |
| F1.BY | W24 | 0.76 | 0.03 |
| F1.BY | W27 | 0.75 | 0.03 |
| F1.BY | W6 | 0.46 | 0.05 |
| F1.BY | W3 | 0.57 | 0.04 |
| F1.BY | W14 | 0.57 | 0.05 |
| F2.BY | W5 | 0.82 | 0.03 |
| F2.BY | W8 | 0.84 | 0.02 |
| F2.BY | W13 | 0.78 | 0.03 |
| F2.BY | W29 | 0.80 | 0.03 |
| F2.BY | W18 | 0.77 | 0.03 |
| F2.BY | W4 | 0.76 | 0.03 |
| F2.BY | W7 | 0.72 | 0.04 |
| F2.BY | W21 | 0.78 | 0.03 |
| F2.BY | W28 | 0.71 | 0.04 |
| F2.WITH | F1 | 0.34 | 0.06 |
| Intercepts | W1 | 2.09 | 0.07 |
| Intercepts | W2 | 2.16 | 0.08 |
| Intercepts | W3 | 3.18 | 0.15 |
| Intercepts | W4 | 3.12 | 0.13 |
| Intercepts | W5 | 3.26 | 0.14 |
| Intercepts | W6 | 3.58 | 0.16 |
| Intercepts | W7 | 3.62 | 0.18 |
| Intercepts | W8 | 2.82 | 0.11 |
| Intercepts | W9 | 2.32 | 0.09 |
| Intercepts | W10 | 2.23 | 0.08 |
| Intercepts | W12 | 2.87 | 0.11 |
| Intercepts | W13 | 2.88 | 0.12 |
| Intercepts | W14 | 3.30 | 0.14 |
| Intercepts | W15 | 2.49 | 0.09 |
| Intercepts | W16 | 2.34 | 0.09 |
| Intercepts | W17 | 2.29 | 0.08 |
| Intercepts | W18 | 2.88 | 0.12 |
| Intercepts | W19 | 2.46 | 0.09 |
| Intercepts | W20 | 2.32 | 0.09 |
| Intercepts | W21 | 2.74 | 0.12 |
| Intercepts | W23 | 3.04 | 0.12 |
| Intercepts | W24 | 2.18 | 0.08 |
| Intercepts | W27 | 2.10 | 0.07 |
| Intercepts | W28 | 2.72 | 0.11 |
| Intercepts | W29 | 2.92 | 0.11 |
| Variances | F1 | 1.00 | 0.00 |
| Variances | F2 | 1.00 | 0.00 |
| Residual.Variances | W1 | 0.46 | 0.05 |
| Residual.Variances | W2 | 0.56 | 0.05 |
| Residual.Variances | W3 | 0.68 | 0.05 |
| Residual.Variances | W4 | 0.42 | 0.05 |
| Residual.Variances | W5 | 0.34 | 0.04 |
| Residual.Variances | W6 | 0.79 | 0.04 |
| Residual.Variances | W7 | 0.48 | 0.05 |
| Residual.Variances | W8 | 0.30 | 0.03 |
| Residual.Variances | W9 | 0.46 | 0.04 |
| Residual.Variances | W10 | 0.37 | 0.04 |
| Residual.Variances | W12 | 0.42 | 0.04 |
| Residual.Variances | W13 | 0.39 | 0.05 |
| Residual.Variances | W14 | 0.68 | 0.05 |
| Residual.Variances | W15 | 0.35 | 0.04 |
| Residual.Variances | W16 | 0.52 | 0.05 |
| Residual.Variances | W17 | 0.37 | 0.04 |
| Residual.Variances | W18 | 0.40 | 0.04 |
| Residual.Variances | W19 | 0.35 | 0.04 |
| Residual.Variances | W20 | 0.47 | 0.05 |
| Residual.Variances | W21 | 0.40 | 0.05 |
| Residual.Variances | W23 | 0.46 | 0.04 |
| Residual.Variances | W24 | 0.42 | 0.04 |
| Residual.Variances | W27 | 0.43 | 0.04 |
| Residual.Variances | W28 | 0.49 | 0.05 |
| Residual.Variances | W29 | 0.35 | 0.04 |

*Note.* F1.BY is the loading on F1; F2.BY is the loading on F2.

F2.WITH is the correlation of F2 with the other factor(s); F3.WITH is the correlation of F3 with the other factor(s).

SE=Standard Error.

**Table C4***The Three-factor Model for the CAQ-W with 25 Items*

| Parameter Type | Item/Factor | Estimate | SE |
| --- | --- | --- | --- |
| F1.BY | W9 | 0.73 | 0.03 |
| F1.BY | W10 | 0.80 | 0.02 |
| F1.BY | W17 | 0.80 | 0.02 |
| F1.BY | W15 | 0.81 | 0.02 |
| F1.BY | W1 | 0.74 | 0.03 |
| F1.BY | W2 | 0.67 | 0.04 |
| F1.BY | W12 | 0.76 | 0.03 |
| F1.BY | W16 | 0.70 | 0.03 |
| F1.BY | W19 | 0.81 | 0.02 |
| F1.BY | W20 | 0.74 | 0.03 |
| F1.BY | W23 | 0.73 | 0.03 |
| F1.BY | W24 | 0.77 | 0.03 |
| F1.BY | W27 | 0.76 | 0.03 |
| F2.BY | W5 | 0.82 | 0.03 |
| F2.BY | W8 | 0.83 | 0.02 |
| F2.BY | W13 | 0.78 | 0.03 |
| F2.BY | W29 | 0.80 | 0.03 |
| F2.BY | W18 | 0.77 | 0.03 |
| F2.BY | W4 | 0.76 | 0.03 |
| F2.BY | W7 | 0.72 | 0.04 |
| F2.BY | W21 | 0.78 | 0.03 |
| F2.BY | W28 | 0.71 | 0.04 |
| F3.BY | W6 | 0.74 | 0.04 |
| F3.BY | W3 | 0.76 | 0.04 |
| F3.BY | W14 | 0.80 | 0.04 |
| F2.WITH | F1 | 0.33 | 0.06 |
| F3.WITH | F1 | 0.66 | 0.04 |
| F3.WITH | F2 | 0.38 | 0.07 |
| Intercepts | W1 | 2.09 | 0.07 |
| Intercepts | W2 | 2.16 | 0.08 |
| Intercepts | W3 | 3.18 | 0.15 |
| Intercepts | W4 | 3.12 | 0.13 |
| Intercepts | W5 | 3.26 | 0.14 |
| Intercepts | W6 | 3.58 | 0.16 |
| Intercepts | W7 | 3.62 | 0.18 |
| Intercepts | W8 | 2.82 | 0.11 |
| Intercepts | W9 | 2.32 | 0.09 |
| Intercepts | W10 | 2.23 | 0.08 |
| Intercepts | W12 | 2.87 | 0.11 |
| Intercepts | W13 | 2.88 | 0.12 |
| Intercepts | W14 | 3.30 | 0.14 |
| Intercepts | W15 | 2.49 | 0.09 |
| Intercepts | W16 | 2.34 | 0.09 |
| Intercepts | W17 | 2.29 | 0.08 |
| Intercepts | W18 | 2.88 | 0.12 |
| Intercepts | W19 | 2.46 | 0.09 |
| Intercepts | W20 | 2.32 | 0.09 |
| Intercepts | W21 | 2.74 | 0.12 |
| Intercepts | W23 | 3.04 | 0.12 |
| Intercepts | W24 | 2.18 | 0.08 |
| Intercepts | W27 | 2.10 | 0.07 |
| Intercepts | W28 | 2.72 | 0.11 |
| Intercepts | W29 | 2.92 | 0.11 |
| Variances | F1 | 1.00 | 0.00 |
| Variances | F2 | 1.00 | 0.00 |
| Variances | F3 | 1.00 | 0.00 |
| Residual.Variances | W1 | 0.45 | 0.05 |
| Residual.Variances | W2 | 0.55 | 0.05 |
| Residual.Variances | W3 | 0.43 | 0.06 |
| Residual.Variances | W4 | 0.42 | 0.05 |
| Residual.Variances | W5 | 0.34 | 0.04 |
| Residual.Variances | W6 | 0.45 | 0.06 |
| Residual.Variances | W7 | 0.48 | 0.05 |
| Residual.Variances | W8 | 0.31 | 0.03 |
| Residual.Variances | W9 | 0.47 | 0.04 |
| Residual.Variances | W10 | 0.36 | 0.04 |
| Residual.Variances | W12 | 0.43 | 0.04 |
| Residual.Variances | W13 | 0.39 | 0.05 |
| Residual.Variances | W14 | 0.36 | 0.06 |
| Residual.Variances | W15 | 0.35 | 0.04 |
| Residual.Variances | W16 | 0.51 | 0.05 |
| Residual.Variances | W17 | 0.36 | 0.04 |
| Residual.Variances | W18 | 0.40 | 0.04 |
| Residual.Variances | W19 | 0.35 | 0.04 |
| Residual.Variances | W20 | 0.46 | 0.05 |
| Residual.Variances | W21 | 0.40 | 0.05 |
| Residual.Variances | W23 | 0.46 | 0.05 |
| Residual.Variances | W24 | 0.41 | 0.04 |
| Residual.Variances | W27 | 0.43 | 0.04 |
| Residual.Variances | W28 | 0.50 | 0.05 |
| Residual.Variances | W29 | 0.35 | 0.04 |

*Note.* F1.BY is the loading on F1; F2.BY is the loading on F2.

F2.WITH is the correlation of F2 with the other factor(s).

SE=Standard Error.

**Table C5***The Three-factor Model for the CAQ-W with 30 Items*

| Parameter Type | Item/Factor | Estimate | SE |
| --- | --- | --- | --- |
| F1.BY | W17 | 0.79 | 0.02 |
| F1.BY | W1 | 0.74 | 0.03 |
| F1.BY | W20 | 0.74 | 0.03 |
| F1.BY | W27 | 0.76 | 0.03 |
| F1.BY | W16 | 0.71 | 0.03 |
| F1.BY | W15 | 0.81 | 0.02 |
| F1.BY | W24 | 0.77 | 0.03 |
| F1.BY | W19 | 0.81 | 0.02 |
| F1.BY | W2 | 0.67 | 0.04 |
| F1.BY | W10 | 0.79 | 0.02 |
| F1.BY | W9 | 0.73 | 0.03 |
| F1.BY | W12 | 0.76 | 0.03 |
| F1.BY | W23 | 0.74 | 0.03 |
| F1.BY | W26 | 0.56 | 0.05 |
| F2.BY | W29 | 0.80 | 0.03 |
| F2.BY | W28 | 0.71 | 0.04 |
| F2.BY | W8 | 0.83 | 0.02 |
| F2.BY | W13 | 0.78 | 0.03 |
| F2.BY | W21 | 0.78 | 0.03 |
| F2.BY | W18 | 0.77 | 0.03 |
| F2.BY | W7 | 0.72 | 0.04 |
| F2.BY | W5 | 0.82 | 0.03 |
| F2.BY | W4 | 0.76 | 0.03 |
| F3.BY | W6 | 0.63 | 0.04 |
| F3.BY | W3 | 0.70 | 0.03 |
| F3.BY | W14 | 0.70 | 0.04 |
| F3.BY | W11 | 0.75 | 0.03 |
| F3.BY | W22 | 0.78 | 0.03 |
| F3.BY | W25 | 0.71 | 0.04 |
| F3.BY | W30 | 0.77 | 0.03 |
| F2.WITH | F1 | 0.32 | 0.06 |
| F3.WITH | F1 | 0.86 | 0.02 |
| F3.WITH | F2 | 0.37 | 0.06 |
| Intercepts | W1 | 2.09 | 0.07 |
| Intercepts | W2 | 2.16 | 0.08 |
| Intercepts | W3 | 3.18 | 0.15 |
| Intercepts | W4 | 3.12 | 0.13 |
| Intercepts | W5 | 3.26 | 0.14 |
| Intercepts | W6 | 3.58 | 0.16 |
| Intercepts | W7 | 3.62 | 0.18 |
| Intercepts | W8 | 2.82 | 0.11 |
| Intercepts | W9 | 2.32 | 0.09 |
| Intercepts | W10 | 2.23 | 0.08 |
| Intercepts | W11 | 3.07 | 0.13 |
| Intercepts | W12 | 2.87 | 0.11 |
| Intercepts | W13 | 2.88 | 0.12 |
| Intercepts | W14 | 3.30 | 0.14 |
| Intercepts | W15 | 2.49 | 0.09 |
| Intercepts | W16 | 2.34 | 0.09 |
| Intercepts | W17 | 2.29 | 0.08 |
| Intercepts | W18 | 2.88 | 0.12 |
| Intercepts | W19 | 2.46 | 0.09 |
| Intercepts | W20 | 2.32 | 0.09 |
| Intercepts | W21 | 2.74 | 0.12 |
| Intercepts | W22 | 2.69 | 0.11 |
| Intercepts | W23 | 3.04 | 0.12 |
| Intercepts | W24 | 2.18 | 0.08 |
| Intercepts | W25 | 2.78 | 0.11 |
| Intercepts | W26 | 2.42 | 0.09 |
| Intercepts | W27 | 2.10 | 0.07 |
| Intercepts | W28 | 2.72 | 0.11 |
| Intercepts | W29 | 2.92 | 0.11 |
| Intercepts | W30 | 2.39 | 0.09 |
| Variances | F1 | 1.00 | 0.00 |
| Variances | F2IN | 1.00 | 0.00 |
| Variances | F3 | 1.00 | 0.00 |
| Residual.Variances | W1 | 0.45 | 0.04 |
| Residual.Variances | W2 | 0.55 | 0.05 |
| Residual.Variances | W3 | 0.52 | 0.05 |
| Residual.Variances | W4 | 0.42 | 0.05 |
| Residual.Variances | W5 | 0.34 | 0.04 |
| Residual.Variances | W6 | 0.60 | 0.05 |
| Residual.Variances | W7 | 0.48 | 0.05 |
| Residual.Variances | W8 | 0.31 | 0.03 |
| Residual.Variances | W9 | 0.46 | 0.04 |
| Residual.Variances | W10 | 0.37 | 0.04 |
| Residual.Variances | W11 | 0.43 | 0.05 |
| Residual.Variances | W12 | 0.42 | 0.04 |
| Residual.Variances | W13 | 0.39 | 0.05 |
| Residual.Variances | W14 | 0.51 | 0.05 |
| Residual.Variances | W15 | 0.35 | 0.04 |
| Residual.Variances | W16 | 0.50 | 0.05 |
| Residual.Variances | W17 | 0.38 | 0.04 |
| Residual.Variances | W18 | 0.40 | 0.04 |
| Residual.Variances | W19 | 0.34 | 0.04 |
| Residual.Variances | W20 | 0.46 | 0.05 |
| Residual.Variances | W21 | 0.40 | 0.05 |
| Residual.Variances | W22 | 0.40 | 0.05 |
| Residual.Variances | W23 | 0.46 | 0.04 |
| Residual.Variances | W24 | 0.41 | 0.04 |
| Residual.Variances | W25 | 0.50 | 0.05 |
| Residual.Variances | W26 | 0.69 | 0.05 |
| Residual.Variances | W27 | 0.42 | 0.04 |
| Residual.Variances | W28 | 0.50 | 0.05 |
| Residual.Variances | W29 | 0.35 | 0.04 |
| Residual.Variances | W30 | 0.41 | 0.05 |

*Note.* F1.BY is the loading on F1; F2.BY is the loading on F2; F3.BY is the loading on F3.

F2.WITH is the correlation of F2 with the other factor(s); F3.WITH is the correlation of F3 with the other factor(s).

SE=Standard Error.

**Table C6***The One-factor Model for the CAQ-GE with 24 Items*

| Parameter Type | Item/Factor | Estimate | SE |
| --- | --- | --- | --- |
| F1.BY | GE1 | 0.66 | 0.04 |
| F1.BY | GE2 | 0.66 | 0.04 |
| F1.BY | GE4 | 0.72 | 0.03 |
| F1.BY | GE5 | 0.81 | 0.02 |
| F1.BY | GE7 | 0.73 | 0.04 |
| F1.BY | GE8 | 0.82 | 0.02 |
| F1.BY | GE9 | 0.74 | 0.03 |
| F1.BY | GE10 | 0.72 | 0.03 |
| F1.BY | GE12 | 0.83 | 0.02 |
| F1.BY | GE16 | 0.72 | 0.04 |
| F1.BY | GE17 | 0.71 | 0.03 |
| F1.BY | GE18 | 0.79 | 0.03 |
| F1.BY | GE20 | 0.83 | 0.02 |
| F1.BY | GE21 | 0.79 | 0.03 |
| F1.BY | GE22 | 0.73 | 0.03 |
| F1.BY | GE24 | 0.78 | 0.03 |
| F1.BY | GE25 | 0.81 | 0.02 |
| F1.BY | GE3 | 0.62 | 0.04 |
| F1.BY | GE6 | 0.60 | 0.04 |
| F1.BY | GE11 | 0.59 | 0.04 |
| F1.BY | GE14 | 0.38 | 0.05 |
| F1.BY | GE15 | 0.63 | 0.05 |
| F1.BY | GE19 | 0.55 | 0.05 |
| F1.BY | GE23 | 0.56 | 0.04 |
| Intercepts | GE1 | 2.91 | 0.11 |
| Intercepts | GE2 | 2.41 | 0.09 |
| Intercepts | GE3 | 2.21 | 0.07 |
| Intercepts | GE4 | 2.28 | 0.08 |
| Intercepts | GE5 | 2.08 | 0.07 |
| Intercepts | GE6 | 2.35 | 0.09 |
| Intercepts | GE7 | 2.06 | 0.07 |
| Intercepts | GE8 | 2.24 | 0.08 |
| Intercepts | GE9 | 1.96 | 0.06 |
| Intercepts | GE10 | 2.05 | 0.07 |
| Intercepts | GE11 | 2.32 | 0.08 |
| Intercepts | GE12 | 1.97 | 0.06 |
| Intercepts | GE14 | 3.04 | 0.13 |
| Intercepts | GE15 | 2.14 | 0.07 |
| Intercepts | GE16 | 2.06 | 0.07 |
| Intercepts | GE17 | 1.73 | 0.05 |
| Intercepts | GE18 | 1.85 | 0.06 |
| Intercepts | GE19 | 2.43 | 0.09 |
| Intercepts | GE20 | 2.18 | 0.07 |
| Intercepts | GE21 | 1.87 | 0.06 |
| Intercepts | GE22 | 1.82 | 0.05 |
| Intercepts | GE23 | 2.34 | 0.08 |
| Intercepts | GE24 | 2.05 | 0.07 |
| Intercepts | GE25 | 2.02 | 0.07 |
| Variances | F1 | 1.00 | 0.00 |
| Residual.Variances | GE1 | 0.57 | 0.05 |
| Residual.Variances | GE2 | 0.56 | 0.05 |
| Residual.Variances | GE3 | 0.62 | 0.05 |
| Residual.Variances | GE4 | 0.49 | 0.04 |
| Residual.Variances | GE5 | 0.34 | 0.04 |
| Residual.Variances | GE6 | 0.64 | 0.05 |
| Residual.Variances | GE7 | 0.47 | 0.05 |
| Residual.Variances | GE8 | 0.33 | 0.03 |
| Residual.Variances | GE9 | 0.46 | 0.04 |
| Residual.Variances | GE10 | 0.49 | 0.05 |
| Residual.Variances | GE11 | 0.65 | 0.05 |
| Residual.Variances | GE12 | 0.31 | 0.03 |
| Residual.Variances | GE14 | 0.85 | 0.04 |
| Residual.Variances | GE15 | 0.61 | 0.06 |
| Residual.Variances | GE16 | 0.49 | 0.05 |
| Residual.Variances | GE17 | 0.49 | 0.04 |
| Residual.Variances | GE18 | 0.38 | 0.05 |
| Residual.Variances | GE19 | 0.69 | 0.05 |
| Residual.Variances | GE20 | 0.32 | 0.03 |
| Residual.Variances | GE21 | 0.37 | 0.04 |
| Residual.Variances | GE22 | 0.46 | 0.04 |
| Residual.Variances | GE23 | 0.68 | 0.05 |
| Residual.Variances | GE24 | 0.39 | 0.04 |
| Residual.Variances | GE25 | 0.35 | 0.04 |

*Note.* F1.BY is the loading on F1.

SE=Standard Error.

**Table C7***The Two-factor Model for the CAQ-GE with 24 Items*

| Parameter Type | Item/Factor | Estimate | SE |
| --- | --- | --- | --- |
| F1.BY | GE1 | 0.65 | 0.04 |
| F1.BY | GE2 | 0.67 | 0.04 |
| F1.BY | GE4 | 0.72 | 0.03 |
| F1.BY | GE5 | 0.83 | 0.02 |
| F1.BY | GE7 | 0.74 | 0.03 |
| F1.BY | GE8 | 0.82 | 0.02 |
| F1.BY | GE9 | 0.74 | 0.03 |
| F1.BY | GE10 | 0.71 | 0.04 |
| F1.BY | GE12 | 0.84 | 0.02 |
| F1.BY | GE16 | 0.72 | 0.04 |
| F1.BY | GE17 | 0.72 | 0.03 |
| F1.BY | GE18 | 0.81 | 0.03 |
| F1.BY | GE20 | 0.83 | 0.02 |
| F1.BY | GE21 | 0.79 | 0.03 |
| F1.BY | GE22 | 0.74 | 0.03 |
| F1.BY | GE24 | 0.78 | 0.03 |
| F1.BY | GE25 | 0.81 | 0.03 |
| F2.BY | GE3 | 0.75 | 0.03 |
| F2.BY | GE6 | 0.74 | 0.03 |
| F2.BY | GE11 | 0.76 | 0.03 |
| F2.BY | GE14 | 0.54 | 0.05 |
| F2.BY | GE15 | 0.84 | 0.02 |
| F2.BY | GE19 | 0.78 | 0.03 |
| F2.BY | GE23 | 0.74 | 0.03 |
| F2.WITH | F1 | 0.69 | 0.04 |
| Intercepts | GE1 | 2.91 | 0.11 |
| Intercepts | GE2 | 2.41 | 0.09 |
| Intercepts | GE3 | 2.21 | 0.07 |
| Intercepts | GE4 | 2.28 | 0.08 |
| Intercepts | GE5 | 2.08 | 0.07 |
| Intercepts | GE6 | 2.35 | 0.09 |
| Intercepts | GE7 | 2.06 | 0.07 |
| Intercepts | GE8 | 2.24 | 0.08 |
| Intercepts | GE9 | 1.96 | 0.06 |
| Intercepts | GE10 | 2.05 | 0.07 |
| Intercepts | GE11 | 2.32 | 0.08 |
| Intercepts | GE12 | 1.97 | 0.06 |
| Intercepts | GE14 | 3.04 | 0.13 |
| Intercepts | GE15 | 2.14 | 0.07 |
| Intercepts | GE16 | 2.06 | 0.07 |
| Intercepts | GE17 | 1.73 | 0.05 |
| Intercepts | GE18 | 1.85 | 0.06 |
| Intercepts | GE19 | 2.43 | 0.09 |
| Intercepts | GE20 | 2.18 | 0.07 |
| Intercepts | GE21 | 1.87 | 0.06 |
| Intercepts | GE22 | 1.82 | 0.05 |
| Intercepts | GE23 | 2.34 | 0.08 |
| Intercepts | GE24 | 2.05 | 0.07 |
| Intercepts | GE25 | 2.02 | 0.07 |
| Variances | F1 | 1.00 | 0.00 |
| Variances | F2 | 1.00 | 0.00 |
| Residual.Variances | GE1 | 0.57 | 0.05 |
| Residual.Variances | GE2 | 0.55 | 0.05 |
| Residual.Variances | GE3 | 0.45 | 0.05 |
| Residual.Variances | GE4 | 0.48 | 0.05 |
| Residual.Variances | GE5 | 0.31 | 0.04 |
| Residual.Variances | GE6 | 0.45 | 0.05 |
| Residual.Variances | GE7 | 0.46 | 0.05 |
| Residual.Variances | GE8 | 0.33 | 0.03 |
| Residual.Variances | GE9 | 0.46 | 0.04 |
| Residual.Variances | GE10 | 0.50 | 0.05 |
| Residual.Variances | GE11 | 0.42 | 0.05 |
| Residual.Variances | GE12 | 0.30 | 0.03 |
| Residual.Variances | GE14 | 0.71 | 0.05 |
| Residual.Variances | GE15 | 0.30 | 0.03 |
| Residual.Variances | GE16 | 0.48 | 0.05 |
| Residual.Variances | GE17 | 0.48 | 0.04 |
| Residual.Variances | GE18 | 0.35 | 0.04 |
| Residual.Variances | GE19 | 0.39 | 0.04 |
| Residual.Variances | GE20 | 0.31 | 0.03 |
| Residual.Variances | GE21 | 0.38 | 0.04 |
| Residual.Variances | GE22 | 0.46 | 0.04 |
| Residual.Variances | GE23 | 0.46 | 0.05 |
| Residual.Variances | GE24 | 0.39 | 0.04 |
| Residual.Variances | GE25 | 0.34 | 0.04 |

*Note.* F1.BY is the loading on F1; F2.BY is the loading on F2.

F2.WITH is the correlation of F2 with the other factor(s).

SE=Standard Error.

**Table C8***The Two-factor Model for the CAQ-GE with 25 Items*

| Parameter Type | Item/Factor | Estimate | SE |
| --- | --- | --- | --- |
| F1.BY | GE1 | 0.65 | 0.04 |
| F1.BY | GE2 | 0.67 | 0.04 |
| F1.BY | GE4 | 0.72 | 0.03 |
| F1.BY | GE5 | 0.83 | 0.02 |
| F1.BY | GE7 | 0.74 | 0.03 |
| F1.BY | GE8 | 0.82 | 0.02 |
| F1.BY | GE9 | 0.74 | 0.03 |
| F1.BY | GE10 | 0.71 | 0.04 |
| F1.BY | GE12 | 0.84 | 0.02 |
| F1.BY | GE16 | 0.72 | 0.04 |
| F1.BY | GE17 | 0.73 | 0.03 |
| F1.BY | GE18 | 0.80 | 0.03 |
| F1.BY | GE20 | 0.83 | 0.02 |
| F1.BY | GE21 | 0.79 | 0.03 |
| F1.BY | GE22 | 0.74 | 0.03 |
| F1.BY | GE24 | 0.78 | 0.03 |
| F1.BY | GE25 | 0.81 | 0.02 |
| F1.BY | GE13 | 0.59 | 0.04 |
| F2.BY | GE3 | 0.75 | 0.03 |
| F2.BY | GE6 | 0.74 | 0.03 |
| F2.BY | GE11 | 0.76 | 0.03 |
| F2.BY | GE14 | 0.54 | 0.05 |
| F2.BY | GE15 | 0.84 | 0.02 |
| F2.BY | GE19 | 0.78 | 0.03 |
| F2.BY | GE23 | 0.74 | 0.03 |
| F2.WITH | F1 | 0.69 | 0.04 |
| Intercepts | GE1 | 2.91 | 0.11 |
| Intercepts | GE2 | 2.41 | 0.09 |
| Intercepts | GE3 | 2.21 | 0.07 |
| Intercepts | GE4 | 2.28 | 0.08 |
| Intercepts | GE5 | 2.08 | 0.07 |
| Intercepts | GE6 | 2.35 | 0.09 |
| Intercepts | GE7 | 2.06 | 0.07 |
| Intercepts | GE8 | 2.24 | 0.08 |
| Intercepts | GE9 | 1.96 | 0.06 |
| Intercepts | GE10 | 2.05 | 0.07 |
| Intercepts | GE11 | 2.32 | 0.08 |
| Intercepts | GE12 | 1.97 | 0.06 |
| Intercepts | GE13 | 1.98 | 0.06 |
| Intercepts | GE14 | 3.04 | 0.13 |
| Intercepts | GE15 | 2.14 | 0.07 |
| Intercepts | GE16 | 2.06 | 0.07 |
| Intercepts | GE17 | 1.73 | 0.05 |
| Intercepts | GE18 | 1.85 | 0.06 |
| Intercepts | GE19 | 2.43 | 0.09 |
| Intercepts | GE20 | 2.18 | 0.07 |
| Intercepts | GE21 | 1.87 | 0.06 |
| Intercepts | GE22 | 1.82 | 0.05 |
| Intercepts | GE23 | 2.34 | 0.08 |
| Intercepts | GE24 | 2.05 | 0.07 |
| Intercepts | GE25 | 2.02 | 0.07 |
| Variances | F1 | 1.00 | 0.00 |
| Variances | F2 | 1.00 | 0.00 |
| Residual.Variances | GE1 | 0.58 | 0.05 |
| Residual.Variances | GE2 | 0.55 | 0.05 |
| Residual.Variances | GE3 | 0.45 | 0.05 |
| Residual.Variances | GE4 | 0.48 | 0.05 |
| Residual.Variances | GE5 | 0.31 | 0.04 |
| Residual.Variances | GE6 | 0.45 | 0.05 |
| Residual.Variances | GE7 | 0.46 | 0.05 |
| Residual.Variances | GE8 | 0.33 | 0.03 |
| Residual.Variances | GE9 | 0.46 | 0.04 |
| Residual.Variances | GE10 | 0.50 | 0.05 |
| Residual.Variances | GE11 | 0.42 | 0.05 |
| Residual.Variances | GE12 | 0.30 | 0.03 |
| Residual.Variances | GE13 | 0.65 | 0.05 |
| Residual.Variances | GE14 | 0.71 | 0.05 |
| Residual.Variances | GE15 | 0.30 | 0.03 |
| Residual.Variances | GE16 | 0.48 | 0.05 |
| Residual.Variances | GE17 | 0.47 | 0.04 |
| Residual.Variances | GE18 | 0.35 | 0.04 |
| Residual.Variances | GE19 | 0.39 | 0.04 |
| Residual.Variances | GE20 | 0.31 | 0.03 |
| Residual.Variances | GE21 | 0.38 | 0.04 |
| Residual.Variances | GE22 | 0.46 | 0.04 |
| Residual.Variances | GE23 | 0.46 | 0.05 |
| Residual.Variances | GE24 | 0.39 | 0.04 |
| Residual.Variances | GE25 | 0.34 | 0.04 |

*Note.* F1.BY is the loading on F1; F2.BY is the loading on F2;.

F2.WITH is the correlation of F2 with the other factor(s). SE=Standard Error.

**Supplementary Material D: Items of CAQ Scale**

Instruction [指导语] : In daily life, we often experience “a bolt from the blue.” How would you typically respond in such situations? Please read the following descriptions carefully and answer according to your actual experience. If some items appear similar, there is no need to compare them; please respond based on your first impression. [生活中我们经常会经历“晴天霹雳”，您在这种情况下会作何反应？请您认真阅读下列描述，并按照自己的实际情况作答。如果出现部分题目相似的情况，无需对比，请根据您的第一感觉做出选择。]

Scoring: A total score can be calculated for each dimension. Items marked with an asterisk (*) are those recommended for deletion based on the factor analysis in the current study.

| **CAQ-W Items** | | Response | | | | | | | |
| --- | --- | --- | --- | --- | --- | --- | --- | --- | --- |
|  |  | 1不符合  [Not at all] | 2 有点符合[Slightly] | 3 比较符合[Moderately] | | 4非常符合 [Very much] | | 5完全符合 [Completely] | |
| **F1: Worry to Avoid Negative Emotional Shifts** | | | | | | | | |  |
| 17 | 我对事情感到担忧，因为允许自己感到快乐最终会容易让自己感觉糟糕。  [I worry about things because allowing myself to feel happy leaves me vulnerable to feeling terrible in the end.] |  |  |  |  | |  | |  |
| 1 | 因为坏事随时都可能发生，所以我觉得担忧会让我更舒服。  [Because bad things could happen at any time, I find it more comfortable to be worried.] |  |  |  |  | |  | |  |
| 20 | 我觉得比起放松的时候，我在担忧的时候更能控制局面。  [I feel like I have more control over the situation when I’m worried than when I am relaxed.] |  |  |  |  | |  | |  |
| 15 | 我担忧是为了防止自己的情绪突然变糟糕。  [I worry to prevent my emotions from dropping suddenly.] |  |  |  |  | |  | |  |
| 16 | 当我担忧时，我感觉我的情绪就不那么脆弱了。  [I feel less emotionally vulnerable when I worry.] |  |  |  |  | |  | |  |
| 27 | 有时候我更喜欢担忧，因为当我感觉良好时，我发现自己会等待坏事发生。  [A part of me prefers to be worried, because when I feel good, I find myself waiting for the other shoe to drop.] |  |  |  |  | |  | |  |
| 24 | 我宁愿担忧而不是感到乐观，因为我知道负面事件会随时带走我的快乐。  [I prefer to worry rather than feel optimistic, because I know that at any moment a negative event could take my happiness away.] |  |  |  |  | |  | |  |
| 2 | 我觉得我在担忧时更能控制自己的情绪。  [When I’m worrying, I feel like I’m more in control of my emotions.] |  |  |  |  | |  | |  |
| 19 | 与其让外部事件左右自己的情绪起伏，我宁愿感到担忧以控制自己的情绪。  [I worry to control my own emotions rather than have external events control my ups and downs.] |  |  |  |  | |  | |  |
| 10 | 我担忧是为了防止情绪的突然变化。  [I worry to guard against sudden shifts in my mood.] |  |  |  |  | |  | |  |
| 9 | 我宁愿让自己担忧以保持稳定的情绪状态，也不愿让自己高兴后又心情变差。  [I worry to maintain a steady emotional state, rather than let myself be happy and later feel bad.] |  |  |  |  | |  | |  |
| 12 | 忧虑比感觉良好后又被消极的事情弄得措手不及要好。  [Worrying is better than feeling good and then being thrown off by a negative event.] |  |  |  |  | |  | |  |
| 23 | 如果我感到担忧，我就能在我需要的时候随时做好有坏情绪到来的心理准备。  [If I worry, I can stay emotionally prepared for as long as I need to.] |  |  |  |  | |  | |  |
| 26^*^ | 在担忧时，我能保持情绪的稳定。  [When I worry I can hold my emotions in a steady state.] |  |  |  |  | |  | |  |
| **Factor2:WorryCreatesandSustainsNegativeEmotion** | | | | | | | | |  |
| 29 | 担忧会增加我的负面情绪。  [Worrying increases my bad feelings.] |  |  |  |  | |  | |  |
| 28 | 担忧对我来说是不愉快的体验。  [Worrying is an unpleasant experience for me.] |  |  |  |  | |  | |  |
| 8 | 担忧会使我感觉很糟糕。  [Worrying makes me feel terrible.] |  |  |  |  | |  | |  |
| 13 | 在我忧虑时，我会一直感到紧张与不安。  [When I worry, I constantly feel on edge.] |  |  |  |  | |  | |  |
| 21 | 当我担忧时，我最终会变得精神紧张。  [When I worry, I end up feeling worked up.] |  |  |  |  | |  | |  |
| 18 | 在我开始担忧时，我会感到更加焦虑不安。  [I feel more agitated when I start worrying.] |  |  |  |  | |  | |  |
| 7 | 当我担忧时，我感到有压力。  [When I am worrying, I feel stressed.] |  |  |  |  | |  | |  |
| 5 | 忧虑使我心情不好。  [Worry keeps me in a bad mood.] |  |  |  |  | |  | |  |
| 4 | 担忧会增加我的焦虑。  [Worrying increases my anxiety.] |  |  |  |  | |  | |  |
| **F3: Worry to Create Positive Contrast** | | | | | | | | |  |
| 6 | 如果我担心最坏的结果，而当结果是好的话，我会更加感激。  [If I worry about the worst outcome, I will appreciate it more when it turns out okay.] |  |  |  |  | |  | |  |
| 3 | 当我担忧一件事会失败但最终却成功时，我更能享受它的成功。  [I enjoy success the most when I worried about failure.] |  |  |  |  | |  | |  |
| 14 | 如果我事先为结果担忧，我会更感激好事发生。  [I am more appreciative of the good things that come if I worried about the outcome beforehand.] |  |  |  |  | |  | |  |
| 11^*^ | 如果我事先担忧，当事情最后结果还好时，我会比事先没有担忧的情况感觉好很多  [If I worry, I feel so much better when things turn out okay than if I had not been worried.] |  |  |  |  | |  | |  |
| 30^*^ | 与其一直期待最好的结果，不如在经历担忧之后再得到最好的结果  [It is better to have worried first and then get the best outcome than to expect the best all along.] |  |  |  |  | |  | |  |
| 22^*^ | 我宁愿在担忧后为好事的发生感到惊喜，也不愿先感觉良好后又为坏事情的发生而感到苦恼。  [I would rather worry and be surprised if something good happens, than feel good and be distressed if something bad happens.] |  |  |  |  | |  | |  |
| 25^*^ | 我发现，如果事情最后的结果是好的，那么这个过程中的担忧是最值得的。  [I find worrying most rewarding when something good happens in the end.] |  |  |  |  | |  | |  |

| **CAQ-GE Items** | | Response | | | | |
| --- | --- | --- | --- | --- | --- | --- |
|  |  | 1不符合  [Not at all] | 2 有点符合[Slightly] | 3 比较符合[Moderately] | 4非常符合 [Very much] | 5完全符合 [Completely] |
| **F1: Creating and Sustaining Negative Emotion to Avoid Negative Contrasts** | | | | | | |
| 18 | 我保持着消极的心境，因为这让我在坏事发生时更容易应对。  [I maintain a negative mood because it makes it easier to cope when bad things happen.] |  |  |  |  |  |
| 5 | 因为坏事随时可能发生，所以提前处于一个沮丧的心情会令我感到更舒服。  [Because bad things could happen at any time, it’s more comfortable to already be in a gloomy mood.] |  |  |  |  |  |
| 7 | 我更倾向于保持悲观，这样当好事发生时我就会感到惊喜。  [I prefer to have a pessimistic outlook, so that I can be pleasantly surprised if something good happens.] |  |  |  |  |  |
| 8 | 我倾向于预测事情会失败，因为我不喜欢对那些万一会落空的事情怀有期待。  [I tend to predict failure because I don’t like to look forward to something in case it doesn’t happen.] |  |  |  |  |  |
| 17 | 我不期待好事的发生，这样一切都会让人感到惊喜。  [I don’t anticipate that anything good will happen so that everything will feel like a pleasant surprise.] |  |  |  |  |  |
| 20 | 我会关注消极的方面，因为这样至少我知道不会有太多让我感觉更糟的事情发生。  [I focus on the negative because at least I know not much can happen that could make me feel worse.] |  |  |  |  |  |
| 9 | 若留意到自己高兴，我常常会立马提醒自己所有可能发生的坏事。  [If I notice I’m feeling happy, I tend to immediately remind myself of all the bad things that could happen.] |  |  |  |  |  |
| 2 | 我倾向于期待最坏的结果，这样我就不会在情绪上感到措手不及。  [I tend to expect the worst outcome so that I am not emotionally caught off guard.] |  |  |  |  |  |
| 12 | 我宁愿现在感觉不好，以免之后需要忍受失去快乐的感觉。  [I prefer to feel bad now so I don’t have to endure losing my happiness later.] |  |  |  |  |  |
| 24 | 我试图把注意力放在可能发生的坏事上，因为这能防止我感到情绪上的脆弱。  [I try to stay focused on the bad things that could happen, because it prevents me from feeling emotionally vulnerable.] |  |  |  |  |  |
| 10 | 我从不抱太大希望，这样我就不会失望。  [I never get my hopes up so that I am not disappointed.] |  |  |  |  |  |
| 25 | 我有时宁愿现在就感觉糟糕，而不是等着看事情发展至最终的结果。  [Sometimes I would rather just feel bad now, instead of having to wait and see how things are going to turn out.] |  |  |  |  |  |
| 16 | 在放松或平静的时候，我会关注负面的事情，以免让我的心情因坏事的发生而突然变化。  [When I am relaxed or calm, I focus on the negative as a way to avoid a sudden shift in my mood if something bad happens.] |  |  |  |  |  |
| 4 | 我宁愿现在感觉糟糕，因为这样我起码不会在坏事发生时体验像过山车式起伏波动的情绪。  [I would rather feel bad now, because at least I won’t experience an emotional rollercoaster if terrible things happen.] |  |  |  |  |  |
| 22 | 允许自己感到快乐最终会容易让自己感觉糟糕。  [Allowing myself to feel happy leaves me vulnerable to feeling terrible in the end.] |  |  |  |  |  |
| 21 | 我宁愿感到情绪低落，也不愿经历起起落落的生活。  [I would rather feel down than have to go through life experiencing ups and downs.] |  |  |  |  |  |
| 1 | 我关注事情消极的方面，因为万一糟糕的事情发生，我想在心理上有所准备。  [I focus on the negative because I want to be emotionally prepared in case something terrible happens.] |  |  |  |  |  |
| 13^*^ | 在心情本来就不好时，我更容易忍受坏消息。  [When I have already been in a bad mood, it has been easier to endure bad news.] |  |  |  |  |  |
| **F2: Discomfort with Emotional Shifts** | | | | | | |
| 19 | 情绪的起伏会让我感到不适。  [When my emotions go up and down, it makes me uncomfortable.] |  |  |  |  |  |
| 11 | 突如其来的坏情绪让我感到十分措手不及。  [It really throws me off when I suddenly feel very bad.] |  |  |  |  |  |
| 23 | 强烈的情绪波动对我来说特别不舒服。  [Strongly fluctuating emotions are particularly unpleasant for me.] |  |  |  |  |  |
| 15 | 情绪的波动会让我感到失控。  [When my emotions fluctuate it makes me feel out of control.] |  |  |  |  |  |
| 6 | 情绪急剧向负面转变会令我感到特别不安。  [I am particularly uneasy with sharp shifts in my negative emotion.] |  |  |  |  |  |
| 3 | 我对自己的情绪变化感到不安。  [I feel uneasy with emotional changes.] |  |  |  |  |  |
| 14 | 我不喜欢自己的情绪受到外部事件的控制而大起大落。  [I don’t like it when external events control my ups and downs.] |  |  |  |  |  |

**Supplementary Material E: Validity Analysis**

**Table E1***Item Redundancy of the CAQ-W*

| Item | AIIC | ITC | 1 | 2 | 3 | 4 | 5 | 6 | 7 | 8 | 9 | 10 | 12 | 13 | 14 | 15 | 16 | 17 | 18 | 19 | 20 | 21 | 23 | 24 | 27 | 28 |
| --- | --- | --- | --- | --- | --- | --- | --- | --- | --- | --- | --- | --- | --- | --- | --- | --- | --- | --- | --- | --- | --- | --- | --- | --- | --- | --- |
| 1 | .62 | .73 |  |  |  |  |  |  |  |  |  |  |  |  |  |  |  |  |  |  |  |  |  |  |  |  |
| 2 | .47 | .54 | .16 |  |  |  |  |  |  |  |  |  |  |  |  |  |  |  |  |  |  |  |  |  |  |  |
| 3 | .63 | .65 | .02 | .05 |  |  |  |  |  |  |  |  |  |  |  |  |  |  |  |  |  |  |  |  |  |  |
| 4 | .70 | .79 | -.02 | -.14 | .05 |  |  |  |  |  |  |  |  |  |  |  |  |  |  |  |  |  |  |  |  |  |
| 5 | .72 | .82 | -.09 | **-.26** | -.13 | .06 |  |  |  |  |  |  |  |  |  |  |  |  |  |  |  |  |  |  |  |  |
| 6 | .69 | .73 | -.10 | -.07 | .00 | .12 | .08 |  |  |  |  |  |  |  |  |  |  |  |  |  |  |  |  |  |  |  |
| 7 | .67 | .75 | -.06 | -.12 | .06 | .02 | .01 | .14 |  |  |  |  |  |  |  |  |  |  |  |  |  |  |  |  |  |  |
| 8 | .72 | .81 | -.06 | **-.21** | -.10 | .01 | .05 | .01 | .05 |  |  |  |  |  |  |  |  |  |  |  |  |  |  |  |  |  |
| 9 | .59 | .71 | -.03 | .00 | .11 | .03 | -.06 | .03 | .04 | -.04 |  |  |  |  |  |  |  |  |  |  |  |  |  |  |  |  |
| 10 | .61 | .75 | -.02 | -.03 | -.01 | .02 | -.02 | -.10 | -.07 | -.03 | -.01 |  |  |  |  |  |  |  |  |  |  |  |  |  |  |  |
| 12 | .57 | .69 | -.04 | -.10 | .07 | .03 | -.01 | .04 | .02 | .00 | .08 | -.02 |  |  |  |  |  |  |  |  |  |  |  |  |  |  |
| 13 | .66 | .73 | .11 | -.09 | .03 | -.07 | -.09 | .11 | .04 | -.02 | .15 | .13 | **.21** |  |  |  |  |  |  |  |  |  |  |  |  |  |
| 14 | .64 | .67 | -.06 | -.04 | -.04 | .02 | -.08 | .04 | .08 | -.09 | .05 | .00 | .07 | .06 |  |  |  |  |  |  |  |  |  |  |  |  |
| 15 | .63 | .77 | -.01 | -.03 | .09 | -.06 | -.09 | -.10 | -.06 | -.04 | -.01 | .09 | .03 | .11 | -.01 |  |  |  |  |  |  |  |  |  |  |  |
| 16 | .62 | .75 | .06 | .13 | .01 | -.10 | -.17 | -.09 | -.08 | -.11 | -.04 | -.04 | .03 | .04 | -.01 | .02 |  |  |  |  |  |  |  |  |  |  |
| 17 | .60 | .71 | -.01 | .02 | -.06 | .02 | .02 | -.04 | .03 | .03 | -.03 | -.03 | -.06 | **.20** | -.06 | -.04 | .01 |  |  |  |  |  |  |  |  |  |
| 18 | .68 | .77 | .05 | -.12 | .00 | -.01 | -.05 | .09 | -.05 | -.01 | .04 | .08 | .16 | .00 | .03 | .08 | -.02 | .09 |  |  |  |  |  |  |  |  |
| 19 | .63 | .78 | -.06 | -.02 | .01 | .03 | -.05 | -.05 | -.03 | -.03 | .00 | -.01 | -.03 | .10 | .04 | .00 | .01 | .01 | .10 |  |  |  |  |  |  |  |
| 20 | .58 | .70 | -.01 | .10 | -.07 | -.07 | -.10 | -.09 | -.09 | -.12 | -.10 | .04 | -.06 | .03 | -.02 | -.03 | .02 | .00 | .02 | .08 |  |  |  |  |  |  |
| 21 | .66 | .74 | .12 | -.14 | .02 | -.03 | -.03 | .02 | -.01 | -.05 | .05 | .08 | .12 | .02 | -.02 | .03 | -.07 | .11 | .01 | .08 | -.02 |  |  |  |  |  |
| 23 | .57 | .70 | -.03 | -.09 | .07 | .07 | -.04 | .07 | -.01 | -.05 | .03 | -.06 | .05 | .14 | .04 | -.05 | -.01 | -.02 | .07 | .01 | -.01 | .14 |  |  |  |  |
| 24 | .62 | .73 | .03 | -.03 | -.06 | .00 | .03 | -.05 | -.01 | .00 | -.01 | -.02 | -.07 | **.20** | .01 | -.02 | -.05 | .02 | .15 | -.02 | -.04 | .18 | .01 |  |  |  |
| 27 | .60 | .71 | .04 | .05 | -.04 | -.02 | .00 | -.07 | .03 | -.02 | -.09 | -.03 | -.13 | .11 | -.01 | -.07 | .02 | .06 | .09 | -.03 | .06 | .12 | -.03 | .04 |  |  |
| 28 | .64 | .71 | -.13 | **-.24** | **-.22** | -.05 | .05 | -.08 | -.07 | .01 | -.07 | -.08 | -.05 | -.10 | -.16 | -.15 | -.19 | .03 | -.05 | -.07 | -.10 | -.06 | -.05 | .01 | -.05 |  |
| 29 | .70 | .80 | -.14 | **-.29** | -.11 | -.03 | .00 | .01 | -.06 | .02 | -.11 | -.09 | -.03 | -.08 | -.04 | -.14 | -.16 | -.03 | -.02 | -.07 | -.15 | -.01 | -.04 | .02 | -.07 | .17 |

*Note.* AIIC=Average inter-item correlation. ITC=Item-total correlations.

Values in the matrix are standardized residual correlations between items, with the eight largest values shown in bold.

**Table E2***Item Redundancy of the CAQ-GE*

| Item | AIIC | ITC | 1 | 2 | 3 | 4 | 5 | 6 | 7 | 8 | 9 | 10 | 11 | 12 | 14 | 15 | 16 | 17 | 18 | 19 | 20 | 21 | 22 | 23 | 24 |
| --- | --- | --- | --- | --- | --- | --- | --- | --- | --- | --- | --- | --- | --- | --- | --- | --- | --- | --- | --- | --- | --- | --- | --- | --- | --- |
| 1 | .41 | .52 |  |  |  |  |  |  |  |  |  |  |  |  |  |  |  |  |  |  |  |  |  |  |  |
| 2 | .47 | .60 | **.18** |  |  |  |  |  |  |  |  |  |  |  |  |  |  |  |  |  |  |  |  |  |  |
| 3 | .52 | .61 | .07 | -.02 |  |  |  |  |  |  |  |  |  |  |  |  |  |  |  |  |  |  |  |  |  |
| 4 | .46 | .57 | .09 | .14 | .00 |  |  |  |  |  |  |  |  |  |  |  |  |  |  |  |  |  |  |  |  |
| 5 | .61 | .76 | .03 | .06 | -.02 | .04 |  |  |  |  |  |  |  |  |  |  |  |  |  |  |  |  |  |  |  |
| 6 | .59 | .71 | .08 | .05 | -.08 | .06 | -.04 |  |  |  |  |  |  |  |  |  |  |  |  |  |  |  |  |  |  |
| 7 | .58 | .73 | -.05 | .02 | -.02 | .04 | .06 | -.04 |  |  |  |  |  |  |  |  |  |  |  |  |  |  |  |  |  |
| 8 | .56 | .72 | .04 | .04 | .04 | .03 | .03 | .04 | .05 |  |  |  |  |  |  |  |  |  |  |  |  |  |  |  |  |
| 9 | .53 | .66 | -.07 | -.03 | .00 | -.09 | -.04 | .01 | -.03 | -.05 |  |  |  |  |  |  |  |  |  |  |  |  |  |  |  |
| 10 | .53 | .65 | .00 | -.05 | .10 | -.02 | -.08 | .07 | -.01 | .05 | -.03 |  |  |  |  |  |  |  |  |  |  |  |  |  |  |
| 11 | .60 | .73 | .03 | -.05 | -.04 | -.03 | **-.13** | .03 | .03 | .01 | .02 | .05 |  |  |  |  |  |  |  |  |  |  |  |  |  |
| 12 | .64 | .80 | -.04 | -.06 | -.04 | .01 | .05 | .05 | .01 | .00 | .04 | .02 | -.02 |  |  |  |  |  |  |  |  |  |  |  |  |
| 14 | .39 | .44 | .03 | -.01 | -.08 | -.01 | -.10 | .06 | -.04 | .02 | .02 | .04 | .04 | .00 |  |  |  |  |  |  |  |  |  |  |  |
| 15 | .60 | .72 | .03 | -.08 | .01 | -.10 | -.09 | -.05 | -.07 | -.01 | .03 | .11 | -.01 | .00 | .00 |  |  |  |  |  |  |  |  |  |  |
| 16 | .57 | .71 | -.06 | -.08 | .06 | -.12 | .00 | -.05 | -.06 | -.08 | .07 | -.05 | .01 | -.04 | -.04 | -.03 |  |  |  |  |  |  |  |  |  |
| 17 | .54 | .61 | **-.17** | -.08 | -.02 | -.09 | -.06 | -.06 | -.01 | -.08 | .02 | .05 | -.01 | -.04 | -.11 | -.01 | .12 |  |  |  |  |  |  |  |  |
| 18 | .61 | .75 | -.10 | -.09 | -.01 | -.05 | .02 | **-.13** | .02 | -.02 | .02 | -.04 | -.03 | -.01 | **-.13** | -.05 | .08 | .08 |  |  |  |  |  |  |  |
| 19 | .64 | .79 | -.03 | -.12 | -.05 | -.08 | **-.14** | -.01 | -.03 | -.02 | .01 | .09 | .02 | .00 | .08 | .05 | -.02 | -.07 | **-.14** |  |  |  |  |  |  |
| 20 | .63 | .80 | .02 | -.06 | .01 | -.05 | -.02 | .00 | -.03 | .00 | .00 | -.02 | -.02 | .04 | .02 | -.01 | .02 | .02 | -.03 | .00 |  |  |  |  |  |
| 21 | .54 | .64 | -.12 | -.09 | .05 | -.10 | -.03 | .07 | -.01 | -.03 | .02 | .00 | .04 | -.03 | -.08 | .08 | -.03 | .03 | .00 | .04 | .00 |  |  |  |  |
| 22 | .51 | .59 | -.11 | **-.14** | .08 | -.11 | -.01 | -.04 | -.05 | -.08 | .04 | -.08 | .03 | -.05 | -.11 | -.03 | .05 | .07 | .02 | -.04 | .03 | .06 |  |  |  |
| 23 | .57 | .69 | .00 | -.07 | -.04 | -.10 | -.09 | -.05 | -.07 | -.03 | .00 | .04 | -.05 | -.03 | .06 | .01 | .00 | -.05 | -.06 | .07 | .03 | .10 | .05 |  |  |
| 24 | .61 | .78 | -.07 | -.07 | .08 | -.01 | -.06 | .00 | -.03 | -.07 | .01 | -.09 | .04 | -.03 | -.01 | .06 | .06 | .00 | .05 | .00 | .01 | .00 | .01 | .07 |  |
| 25 | .51 | .63 | -.02 | -.02 | .05 | .02 | -.02 | .07 | .00 | .01 | -.03 | -.01 | .01 | -.02 | .00 | -.01 | -.04 | -.03 | .02 | -.03 | -.01 | .01 | .02 | .03 | .03 |

*Note.* AIIC=Average inter-item correlation. ITC=Item-total correlations.

Values in the matrix are standardized residual correlations between items, with the seven largest values shown in bold.

**Table E3**
*CAQ Scales and Subscales: GAD and Non-Anxious Participant Means, t-scores, and Effect Sizes*

| Scale | GAD | | Non-Anxious | | *t*-score (*df*) | Cohen’s d |
| --- | --- | --- | --- | --- | --- | --- |
|  | (*N*=37) | | (*N*=37) | |  |  |
|  | *M SD* | | *M SD* | |  |  |
| CAQ-W Total | 3.28 | 0.69 | 1.97 | 0.69 | 8.18 (72) | 1.9 |
| CAQ-W F1 | 2.55 | 0.94 | 1.6 | 0.79 | 4.68 (70) | 1.09 |
| CAQ-W F2 | 4.13 | 0.82 | 2.28 | 0.96 | 8.92 (70.2) | 2.07 |
| CAQ-W F3 | 3.94 | 1.06 | 2.67 | 1.36 | 4.48 (67.8) | 1.04 |
| CAQ-GE Total | 3.02 | 0.83 | 1.82 | 0.64 | 6.93 (67.5) | 1.61 |
| CAQ-GE F1 | 2.7 | 1.01 | 1.72 | 0.72 | 4.8 (64.9) | 1.12 |
| CAQ-GE F2 | 3.77 | 0.89 | 2.05 | 0.79 | 8.8 (70.9) | 2.05 |

*Note*. All tests were significant at *p* < .001.

**Table E4**
*Classification Accuracy for CAQ-W and CAQ-GE Cut Scores*

| Scale Score | Sensitivity | Specificity | Correctly  Classified  (%) | False  Positive  Rate | False  Negative  Rate | PPV | NPV | PPV  (20% assumed) ^a^ | NPV  (20% assumed) ^a^ |
| --- | --- | --- | --- | --- | --- | --- | --- | --- | --- |
| **CAQ-W** | | | | | | | |  |  |
| 2.34 | .95 | .74 | .82 | .26 | .05 | .69 | .96 | .47 | .98 |
| 2.38 | .92 | .75 | .82 | .25 | .08 | .69 | .94 | .48 | .97 |
| 2.42 | .92 | .77 | .83 | .23 | .08 | .71 | .94 | .50 | .97 |
| 2.46 | .92 | .80 | .85 | .20 | .08 | .74 | .94 | .54 | .98 |
| **2.50** | **.92** | **.82** | **.86** | **.18** | **.08** | **.76** | **.94** | **.56** | **.98** |
| 2.56 | .86 | .82 | .84 | .18 | .14 | .74 | .91 | .55 | .96 |
| 2.62 | .81 | .82 | .82 | .18 | .19 | .73 | .88 | .53 | .95 |
| 2.66 | .78 | .82 | .81 | .18 | .22 | .73 | .86 | .52 | .94 |
| 2.70 | .78 | .84 | .82 | .16 | .22 | .74 | .86 | .54 | .94 |
| **CAQ-GE** | | | | | | | |  |  |
| 1.81 | .95 | .59 | .72 | .41 | .05 | .58 | .95 | .37 | .98 |
| 1.85 | .92 | .61 | .72 | .39 | .08 | .59 | .93 | .37 | .97 |
| 1.90 | .86 | .64 | .72 | .36 | .14 | .59 | .89 | .37 | .95 |
| 1.94 | .86 | .67 | .74 | .33 | .14 | .62 | .89 | .40 | .95 |
| 2.00 | .86 | .69 | .76 | .31 | .14 | .63 | .89 | .41 | .95 |
| 2.06 | .86 | .72 | .78 | .28 | .14 | .65 | .90 | .44 | .96 |
| 2.13 | .86 | .74 | .79 | .26 | .14 | .67 | .90 | .45 | .96 |
| 2.19 | .84 | .74 | .78 | .26 | .16 | .66 | .88 | .44 | .95 |
| 2.25 | .78 | .79 | .79 | .21 | .22 | .69 | .86 | .48 | .94 |
| **2.31** | **.78** | **.84** | **.82** | **.16** | **.22** | **.74** | **.86** | **.54** | **.94** |
| 2.35 | .76 | .84 | .81 | .16 | .24 | .74 | .85 | .54 | .93 |
| 2.40 | .73 | .85 | .81 | .15 | .27 | .75 | .84 | .55 | .93 |
| 2.44 | .73 | .87 | .82 | .13 | .27 | .77 | .84 | .58 | .93 |
| 2.50 | .70 | .87 | .81 | .13 | .30 | .76 | .83 | .57 | .92 |
| 2.58 | .65 | .87 | .79 | .13 | .35 | .75 | .80 | .55 | .91 |

*Note.* Number in boldface represents the cut-off point for maximally balanced sensitivity and specificity levels. PPV = positive predictive value; NPV = negative predictive value.

^a^ PPV and NPV were calculated assuming a prevalence of 20%.

**Figure E1***The ROC Curve for the CAQ-W*

*
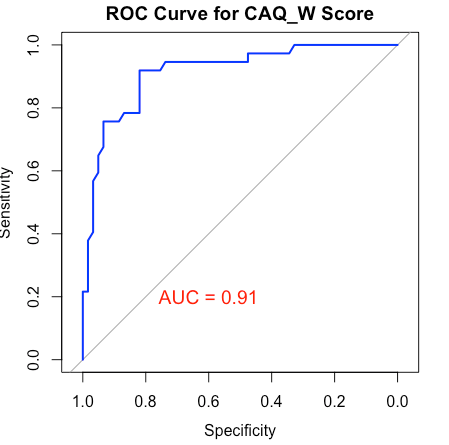
*

**Figure E2***The ROC Curve for the CAQ-GE*

*
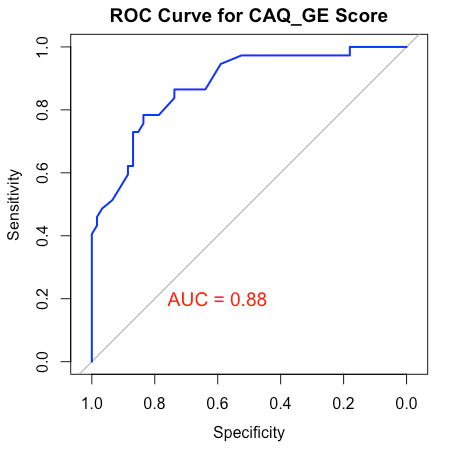
*
